# Supplementary material for: Dialects of Madagascar
Source: PLoS One. 2020 Oct 2;15(10):e0240170. doi: 10.1371/journal.pone.0240170 (PMC7531839; doi:10.1371/journal.pone.0240170)
Supplement: S1 Dataset — The complete dataset of 207 items Swadesh lists for 60 Malagasy variants in text format. The entire 12,420 items dataset which can be consulted here can be freely used, as long as its origin is quoted. (PDF) [file pone.0240170.s001.pdf]

Malagasy Swadesh lists

collected by Maurizio Serva

elaborated by Michele Pasquini

November 2019 update

(click on the dialect name to get it)

1 - Sakalava (Ambanja)
2 - Sihanaka (Ambatondrazaka)
3 - Antankarana (Ambilobe)
4 - Antandroy (Ambovombe)
5 - Mahafaly (Ampanihy)
6 - Mikea (Ampoakafo)
7 - Betsimisaraka (Antalaha)
8 - Merina (Antananarivo)
9 - Bara (Betroka)
10 - Zafisoro (Farafangana)
11 - Betsimisaraka (Fenoarivo-Est)
12 - Betsileo (Fianarantsoa)
13 - Betsimisaraka (Mahanoro)
14 - Sakalava (Maintirano)
15 - Sakalava (Mahajanga)
16 - Antaimoro (Manakara)
17 - Antambohoaka (Mananjary)
18 - Tsimihety (Mandritsara)
19 - Masikoro (Miary)
20 - Sakalava (Morondava)
21 - Antanosy (Tolagnaro)
22 - Vezo (Toliara)
23 - Antaisaka (Vangaindrano)
24 - Antankarana (Vohemar)
25 - Betsileo (Ambositra)
26 - Betsileo (Ambalavao)
27 - Antanalana (Itampolo)
28 - Vezo (Morombe)
29 - Antanosy (Bezaha)
30 - Tanala (Ifanadiana)

0 - English reference
31 - Bara (Ranohira)
32 - Antanalana (Manorofify)
33 - Antandroy (Toliara)
34 - Antanalana (Anakao)
35 - Betsimisaraka (Marolambo)
36 - Betsimisaraka (Antsiranana)
37 - Betsimisaraka (Brickaville)
38 - Betsimisaraka (Toamasina)
39 - Betsimisaraka (Mananara)
40 - Tsimihety (Mampikony)
41 - Tsimihety (Andapa)
42 - Nosy Boraha (Ambodifotatra)
43 - Bara (Beroroha)
44 - Sakalava (Miandrivazo)
45 - Vezo (Morondava)
46 - Bara (Ihosy)
47 - Tsimihety (Antsohihy)
48 - Merina (Maevatanana)
49 - Sakalava (Besalampy)
50 - Betsimisaraka (Tanambao Manampotsy)
51 - Sihanaka (Morarano Chrome)
52 - Betsileo (Ambohimahasoa)
53 - Betsimisaraka (Maroantsetra)
54 - Merina (Analavory)
55 - Betsimisaraka (Sahavato)
56 - Mahafaly (Ejeda)
57 - Antanosy (Belamoty)
58 - Sihanaka (Andilamena)
59 - Antandroy (Tsihombe)
60 - Sakalava (Belon'i Tsiribihina)

English reference							
1	I	53	stick	105	to smell (perceive)	157	sand
2	you (singular)	54	fruit	106	to fear	158	dust
3	he	55	seed (semen)	107	to sleep	159	earth
4	we	56	leaf	108	to live	160	cloud
5	you (plural)	57	root	109	to die	161	fog
6	they	58	bark (of a tree)	110	to kill	162	sky
7	this	59	flower	111	to fight	163	wind
8	that	60	grass	112	to hunt	164	snow
9	here	61	rope	113	to hit	165	ice
10	there	62	skin	114	to cut	166	smoke
11	who	63	meat	115	to split	167	fire
12	what	64	blood	116	to stab	168	ash
13	where	65	bone	117	to scratch	169	to burn
14	when	66	fat (noun)	118	to dig	170	road
15	how	67	egg	119	to swim	171	mountain
16	not	68	horn	120	to fly	172	red
17	all	69	tail	121	to walk (to go)	173	green
18	many	70	feather	122	to come	174	yellow
19	some	71	hair (on the head)	123	to lie (state, as in a bed)	175	white
20	few	72	head	124	to sit (state)	176	black
21	other	73	ear	125	to stand (state)	177	night
22	one	74	eye	126	to turn (intransitive)	178	day
23	two	75	nose	127	to fall	179	year
24	three	76	mouth	128	to give	180	warm
25	four	77	tooth	129	to hold	181	cold
26	five	78	tongue	130	to squeeze	182	full
27	big	79	fingernail	131	to rub	183	new
28	long	80	foot	132	to wash	184	old
29	wide	81	leg	133	to wipe	185	good
30	thick	82	knee	134	to pull	186	bad
31	heavy	83	hand	135	to push	187	rotten
32	small	84	wing	136	to throw	188	dirty
33	short	85	belly	137	to tie	189	straight
34	narrow	86	guts	138	to sew	190	round
35	thin	87	neck	139	to count	191	sharp (as a knife)
36	woman	88	back (of a person)	140	to say	192	dull (as a knife)
37	man (adult male)	89	breast	141	to sing	193	smooth
38	man (human being)	90	heart	142	to play	194	wet
39	child	91	liver	143	to float	195	dry
40	wife	92	to drink	144	to flow	196	correct (just, right)
41	husband	93	to eat	145	to freeze	197	near
42	mother	94	to bite	146	to swell	198	far
43	father	95	to suck	147	sun	199	right (adj.)
44	animal	96	to spit	148	moon	200	left (adj.)
45	fish	97	to vomit	149	star	201	at
46	bird	98	to blow	150	water	202	in
47	dog	99	to breathe	151	rain	203	with
48	louse	100	to laugh	152	river	204	and
49	snake	101	to see	153	lake	205	if
50	worm	102	to hear	154	sea	206	because
51	tree	103	to know (facts)	155	salt	207	name
52	forest	104	to think	156	stone		

1 - Sakalava (Ambanja)

1	zaho	53	kobay	105	maharegny	157	jia
2	anao	54	voankazo	106	mavozo	158	laposiara
3	izy	55	ambeo	107	mandry	159	fotaka
4	atsika	56	ravigny	108	velogno	160	rondro
5	anareo	57	vahatra	109	maty	161	zavogno
6	iro	58	hodikakazo	110	mamono	162	lagnitry
7	ty	59	felany	111	miady	163	tsiko
8	zegny	60	ahitry	112	mihaza	164	laneige
9	eto	61	tady	113	mamopoko	165	lagilasy
10	ry	62	hoditra	114	manapaka	166	setroko
11	azovy	63	hena	115	mamaky	167	motro
12	ino	64	lio	116	manomboko meso	168	jofo
13	aia	65	taholagna	117	mikiky	169	magnoro
14	mbiagna	66	jabora	118	mangady	170	lalagna
15	karakory	67	antody	119	milomagno	171	bongo
16	tsy	68	ampondo	120	magnembana	172	mena
17	ziaby	69	ohy	121	mandeha	173	maitso
18	maro	70	volomborogno	122	navy	174	fondragna
19	helihely	71	fagneva	123	mandry	175	malandy
20	hely	72	loha	124	mipetraka	176	joby
21	hafa	73	sofigny	125	mitsangagna	177	aligny
22	araiky	74	maso	126	mihodigny	178	andra
23	aroe	75	orogno	127	lavo	179	taogno
24	telo	76	vava	128	magnamia	180	mafana
25	aifatra	77	nify	129	mitagna	181	manintsy
26	dimy	78	lela	130	mamiaka	182	feno
27	maventy	79	angofo	131	mandrokotro	183	vao
28	lava	80	vity	132	manasa	184	matoe
29	mivelatra	81	peky	133	mamitry	185	tsara
30	matevigny	82	lohalitry	134	mitifitry	186	ratsy
31	mavesatra	83	tagnana	135	miposy	187	montraka
32	hely	84	elatra	136	mitoraka	188	maloto
33	fohiky	85	kibo	137	manohy	189	mahitsy
34	maletry	86	tsontsory	138	manjaitry	190	boribory
35	matify	87	vozogno	139	magnisaka	191	maragnitry
36	magnangy	88	tahezagna	140	mivolagna	192	magofogno
37	lelahy	89	tratra	141	mihira	193	malamatra
38	olo	90	fo	142	misoma	194	leny
39	tsaiky	91	aty	143	miefogno	195	maiky
40	vady	92	migiaka	144	mivalagna	196	marigny
41	vady	93	mihina	145	mandry	197	marikitry
42	nindry	94	magnekitry	146	mivonto	198	lavitry
43	baba	95	minono	147	masoandro	199	ankahery
44	biby	96	mihaka	148	fanjava	200	ankavia
45	laoko	97	mandoa	149	lakintagna	201	agny
46	vorogno	98	mitsitro	150	rano	202	agnatiny
47	fandroaka	99	miaigny	151	mahaleny	203	miaraka
48	hao	100	mitokiky	152	tegnandrano	204	ndraiky
49	bibilava	101	mahita	153	matsabory	205	izy koa
50	hankagna	102	mitandregny	154	ranomasigny	206	fotony
51	kakazo	103	mahay	155	sira	207	agnaragna
52	atiala	104	mandiniky	156	vato		

2 - Sihanaka (Ambatondrazaka)							
1	za	53	tapakazo	105	mifofona	157	fasika
2	ianao	54	voankazo	106	mataotra	158	vovoka
3	izy	55	voa	107	matory	159	tany
4	antsika	56	ravina	108	velona	160	abakabaka
5	ianareo	57	faka	109	maty	161	zavona
6	ry zareo	58	hodikitay	110	mamono	162	lanitra
7	ty	59	vonikazo	111	miady	163	rivotra
8	izany	60	ahitra	112	mihaza	164	anvandra
9	eto	61	tady	113	mandaroka	165	glasy
10	akao	62	oditra	114	manapaka	166	setroka
11	iza	63	hena	115	mamaky	167	afo
12	inona	64	lio	116	mitoboka antsy	168	lavenona
13	akaiza	65	taolagna	117	maniky	169	mandoro
14	ovina	66	matavy	118	mitombana	170	lalana
15	ahoana	67	atody	119	milomano	171	vohitra
16	tsy	68	tandroka	120	magnembana	172	mena
17	izy daholo	69	rambo	121	mandeha	173	maitso
18	betsaka	70	volomborona	122	avy	174	mavo
19	sasisasy	71	volo	123	matory	175	fotsy
20	kitika	72	loha	124	mipetraka	176	mainty
21	hafa	73	sofina	125	mijaridina	177	alina
22	raika	74	maso	126	mihodina	178	andro
23	roa	75	orona	127	mikarapoka	179	taona
24	telo	76	vava	128	manome	180	mafana
25	efatra	77	nify	129	mitantana	181	mangatsiaka
26	dimy	78	lela	130	mifinjy	182	feno
27	ngenda	79	angofo	131	manakisina	183	vaovao
28	lava	80	tongotra	132	manasa	184	antitra
29	malalaka	81	ranjo	133	mamafa	185	tsara
30	mivalampatra	82	lohalika	134	mikendry	186	ratsy
31	mavesatra	83	tanana	135	manosika	187	masiso
32	bilitika	84	elatra	136	manoraka	188	maloto
33	pota	85	kibo	137	mamatotra	189	mahitsy
34	tery	86	tsinay	138	manjaitra	190	boribory
35	manify	87	tenda	139	manisa	191	matsiko
36	vehivavy	88	lakambo	140	miteny	192	dombo
37	lelahy	89	tratra	141	mihira	193	malama
38	olona	90	fo	142	midaola	194	mando
39	ankizy	91	aty	143	mitsangevana	195	maina
40	vady	92	misotro	144	mikoriana	196	mety
41	vady	93	inana	145	mivaingana	197	akaiky
42	neny	94	manaikitra	146	mibontsina	198	alavitra
43	dada	95	mitsetsitra	147	masova	199	ankavanana
44	biby	96	mandrora	148	volana	200	ankavia
45	lapia	97	mandoa	149	kintana	201	any
46	vorona	98	mitsoka	150	rano	202	anaty
47	amboa	99	miaina	151	orana	203	amin ny
48	hao	100	mivanitika	152	renirano	204	sy
49	bibilava	101	mitazana	153	farihy	205	raha
50	felika	102	mihaino	154	ranomasina	206	satria
51	kakazo	103	mahay	155	sira	207	anarana
52	ala	104	mieritreritra	156	vato		

3 - Antankarana (Ambilobe)							
1	zaho	53	kibay	105	maharegny	157	jia
2	anao	54	voankazo	106	mavozo	158	laposiera
3	izy	55	ambio	107	matoro	159	tany
4	atsika	56	ravigny	108	velogno	160	rondro
5	anaro	57	fototro	109	maty	161	zavogno
6	iro	58	hodikakazo	110	mamono	162	lagnitry
7	ity	59	folera	111	miady	163	tsiko
8	izegny	60	ahitry	112	mihaza	164	lanezy
9	eto	61	tady	113	mamopoko	165	ranomandry
10	iry	62	hoditry	114	mandidy	166	setroko
11	zovy	63	hena	115	mamaky	167	motro
12	ino	64	lio	116	mitomboko meso	168	jofo
13	aia	65	taolagna	117	magnihikihy	169	magnoro
14	ombiagna	66	vondraka	118	mangady	170	lalabe
15	akory	67	atody	119	milomagno	171	bongo
16	tsy	68	ampondo	120	mitiligny	172	mena
17	jiaby	69	ohy	121	mandeha	173	maitso
18	maro	70	volovolomborogno	122	navy	174	vogny
19	vitsy	71	fagneva	123	mandry	175	malandy
20	hely	72	loha	124	mipetraka	176	joby
21	hafa	73	sofigny	125	mitsangana	177	aligny
22	araiky	74	fagnenty	126	mierigny	178	andra
23	aroe	75	orogno	127	lavo	179	taogno
24	telo	76	vava	128	magnomia	180	mafana
25	efatra	77	nify	129	mitagna	181	manintsy
26	dimy	78	lela	130	misery	182	feno
27	maventy	79	angofo	131	magnisiky	183	vao
28	lava	80	vity	132	manasa	184	matoe
29	malalaka	81	vavitsy	133	mamotro	185	tsara
30	matevigny	82	lohalitry	134	mitifitry	186	ratsy
31	mavesatra	83	tagnana	135	mandronjy	187	motraka
32	hely	84	elatra	136	magnatsipy	188	maloto
33	foehiky	85	troko	137	mamehy	189	mahitsy
34	maletry	86	tsontsory	138	manjaitry	190	boribory
35	matify	87	vozogno	139	magnisa	191	maragnitry
36	magnangy	88	tahezagna	140	mivolagna	192	magofogno
37	lelahy	89	tratra	141	mihira	193	madoso
38	olo	90	fo	142	misoma	194	mando
39	tsaiky	91	aty	143	milailay	195	maiky
40	vady	92	migiaka	144	mivalagna	196	mety
41	vady	93	mihina	145	mandry	197	marikitry
42	indry	94	magnekitry	146	mivonto	198	lavitry
43	baba	95	minono	147	masoandra	199	ankahery
44	kaka	96	mikahaka	148	fanjava	200	ankavia
45	laoko	97	mandoa	149	lakinta	201	agny
46	vorogno	98	mitsotro	150	rano	202	agnaty
47	fandroaka	99	miaigny	151	mahalegny	203	amin ny
48	hao	100	mitokiky	152	tegnandrano	204	ndraiky
49	biby	101	mizaha	153	matsabory	205	izy koa
50	hankagna	102	mitandregny	154	ranomasigny	206	fotony
51	kakazo	103	mahay	155	sira	207	agnaragna
52	atiala	104	mieritreritry	156	vato		

4 - Antandroy (Ambovombe)

1	raho	53	kobay	105	magnatso	157	faseke
2	irehe	54	voankazo	106	votro	158	deboke
3	ireke	55	voa	107	mirotse	159	tane
4	itika	56	rave e	108	velogne	160	rahogne
5	nareo	57	foto e	109	vilasy	161	zavogne
6	iereo	58	holinkatay	110	mamono	162	lagnitse
7	itoy	59	voninkazo	111	mialy	163	tioke
8	igne	60	akata	112	mikodebe	164	ranomivongagne
9	etoagne	61	taly	113	mijera	165	ranomivongagne
10	ao	62	holitse	114	manankape	166	setroke
11	ia	63	hena	115	mandrara	167	afo
12	inogne	64	lio	116	mitomboke mesa	168	lavenoke
13	aia	65	taolagne	117	mikihy	169	magnoro
14	ombia	66	vondrake	118	magnaly	170	lalagne
15	akore	67	atoly	119	milaogne	171	vohitse
16	tsy	68	tsifa	120	mitiligne	172	mena
17	iaby	69	ohy	121	magnavelo	173	maintso
18	maro	70	volomborogne	122	avy	174	mavogne
19	kelekele	71	maroy	123	matory	175	foty
20	kede	72	agnabony	124	mitoboke	176	mainte
21	hafa	73	ravembia	125	mitsangagne	177	hariva
22	raike	74	fihaino	126	mihodigne	178	andro
23	roe	75	fagnatsogne	127	tonta	179	taogne
24	telo	76	falie	128	manolotse	180	matsovoke
25	efatre	77	famotse	129	mitagne	181	manitsy
26	dime	78	fameleke	130	mamihigne	182	feno
27	bey	79	hoho	131	midraso	183	vaovao
28	lava	80	fandia	132	manasa	184	antetre
29	mahalagne	81	ranjo	133	mamafa	185	soa
30	matevegne	82	ongotse	134	mitifitse	186	raty
31	mavesatse	83	fita	135	manoseke	187	logne
32	kedekede	84	elatse	136	mitorake	188	maloto
33	bory	85	troke	137	mamehe	189	mahintsy
34	tere	86	tsinay	138	manjaitse	190	boribory
35	manify	87	vozogne	139	magnisake	191	maragnetse
36	ampela	88	lambosy	140	misaontsy	192	domoke
37	lahilahy	89	aragna	141	mibeko	193	malama
38	ndaty	90	agnarofo	142	mihisa	194	legne
39	ajaja	91	ate	143	mihafogne	195	maike
40	valy	92	minogne	144	mirarake	196	mete
41	valy	93	mikama	145	mivongagne	197	marine
42	nene	94	manifatse	146	mibotignake	198	lavitse
43	rae	95	mitsintsike	147	masoandro	199	havanagne
44	biby	96	mandrehoke	148	volagne	200	havia
45	fiagne	97	mandoa	149	kintagne	201	agne
46	vorogne	98	mifioke	150	rano	202	agnate e
47	amboa	99	miaigne	151	oragne	203	amin ny
48	hao	100	lagnake	152	renerano	204	sy
49	meregne	101	mahavazoho	153	sihanake	205	naho
50	soko	102	mijanjigne	154	riake	206	satria
51	hatay	103	mahafohigne	155	sira	207	tahina
52	ala	104	mieretreretre	156	vato		

5 - Mahafaly (Ampanihy)

1	iaho	53	kobay	105	magnimbo	157	faseke
2	iha	54	voanketae	106	mavaka	158	deboke
3	ireke	55	voae	107	miroro	159	tane
4	itika	56	ravine	108	velo	160	rahongne
5	iareo	57	vahae	109	mate	161	mika
6	ireo	58	holinkatae	110	mamono	162	lagnitse
7	itike	59	folera	111	mialy	163	tioke
8	iroke	60	ahetse	112	mihaza	164	havandra
9	etoa	61	taly	113	mamango	165	ranomandre
10	ao	62	holine	114	mandily	166	setroke
11	ia	63	hena	115	mamaky	167	afo
12	ino	64	lio	116	mitomboke meso	168	lavenoke
13	aia	65	taola	117	magnihy	169	magnoro
14	ombia	66	vondrake	118	mihaly	170	lala
15	talilio	67	atoly	119	milagno	171	vohitse
16	tsie	68	tsifa	120	mitily	172	mena
17	iaby	69	ramboe	121	mandeha	173	maitso
18	maro	70	volomboro	122	avy	174	vogne
19	tsy ampe	71	volo	123	mandre	175	foty
20	kele	72	loha	124	mitoboke	176	mainte
21	hafa	73	sofy	125	miongaha	177	haleke
22	raike	74	fijere	126	mihodike	178	andro
23	roe	75	oro	127	lavo	179	tao
24	telo	76	fitava	128	magnomey	180	mae
25	efatse	77	nife	129	mifampitanjake	181	manintsy
26	lime	78	lela	130	mipiritse	182	feno
27	bey	79	hoho	131	mapikasoke	183	vao
28	lava	80	tomboke	132	manasa	184	antetse
29	malalake	81	ranjo	133	mipio	185	soa
30	mateve	82	ongotse	134	mitifitse	186	raty
31	mavesatse	83	tagna	135	manoseke	187	taratavohe
32	masay	84	elae	136	mitorake	188	maloto
33	boribory	85	troke	137	mandrohy	189	mahity
34	titse	86	tinay	138	mitrebeke	190	boribory
35	matify	87	vozo	139	magnisake	191	maragnitse
36	ampisafe	88	lambosy	140	misafa	192	dobo
37	zaranjaka	89	tratra	141	miantsa	193	malama
38	ndaty	90	fo	142	mihisa	194	mando
39	ajaja	91	ate	143	mihafo	195	maike
40	valy	92	mino	144	mandeha	196	ie
41	valy	93	mitava	145	mandreke	197	marine
42	nene	94	mitifatse	146	mibokinake	198	lavitse
43	baba	95	mamiake	147	andro	199	havagna
44	biby	96	mandrehoke	148	vola	200	havia
45	fia	97	mandoa	149	kita	201	angne
46	voro	98	mitioke	150	rano	202	ampoe
47	amboa	99	miay	151	ora	203	amine
48	hao	100	homehe	152	ranobey	204	naho
49	mere	101	mahisake	153	sihanaky	205	laha
50	soko	102	mitsano	154	riake	206	safe
51	kata	103	mahay	155	sira	207	tahina
52	ala	104	mandineke	156	vato		

6 - Mikea (Ampoakafo)

1	zaho	53	kobay	105	mahare	157	tane
2	iha	54	voanikazo	106	matahotse	158	lemboke
3	ie	55	voane	107	mierotre	159	tane
4	tsika	56	ravine	108	velo	160	hiboke
5	nareo	57	vakany	109	mate	161	ando
6	roze	58	faninkazo	110	mamono	162	monto
7	toy	59	folera	111	mialy	163	tsioke
8	eroy	60	akata	112	mangoro	164	ranovory
9	etoa	61	hosy	113	mamango	165	mangatsakatsake
10	ao	62	holitse	114	mandidy	166	setroke
11	ia	63	hena	115	mamatsike	167	bolo
12	ino	64	fere	116	mitomboke	168	lavenoke
13	aia	65	taola	117	manaotse	169	manoro
14	mbia	66	vondrake	118	mihale	170	lala
15	magnino	67	atoly	119	milagno	171	anabo
16	tsie	68	tsifa	120	tomily	172	mena
17	iabe	69	hohy	121	mandeha	173	maitso
18	maro	70	volo	122	havy	174	vogne
19	tsyampeampe	71	volondoa	123	miroro	175	foty
20	kelekele	72	kabeso	124	mipetsake	176	jobe
21	hata	73	sofy	125	mitsanga	177	ale
22	raike	74	maso	126	mivalike	178	andro
23	roe	75	oro	127	lavo	179	tao
24	telo	76	vava	128	magnome	180	mafana
25	efatse	77	hy	129	manja	181	manintsy
26	lime	78	lela	130	mandrambe	182	atsiky
27	be	79	hoho	131	mazaotse	183	vao
28	lava	80	tomboke	132	manasa	184	antitse
29	malalake	81	taola maike	133	mamafa	185	soa
30	mateve	82	pokopoko	134	mitititse	186	raty
31	mavesatse	83	tagna	135	mandronje	187	mavay
32	kelekele	84	helatse	136	mitorake	188	maloto
33	boriborike	85	tsoke	137	mamehe	189	mahity
34	tsiombe	86	tinay	138	mitsebeke	190	laro
35	matife	87	vozo	139	magnisake	191	masio
36	ampela	88	lambosy	140	miola	192	madomoke
37	johary	89	tratra	141	miantsa	193	madoso
38	olo	90	fo	142	misa	194	le
39	aja	91	ate	143	mihafo	195	maike
40	valy	92	mihino	144	mikororosy	196	mete
41	valy	93	homa	145	mandre	197	marineke
42	nene	94	magnehitse	146	mitsoke	198	lavitse
43	baba	95	minono	147	masoandro	199	ankavana
44	tsiboko	96	mandrehoke	148	zavavola	200	ankavia
45	fia	97	mandoa	149	basia	201	agne
46	voro	98	mitsoke	150	rano	202	anaty
47	alika	99	miai	151	ora	203	misondry
48	hao	100	mihehe	152	vavarano	204	miakake
49	bibilava	101	mahita	153	ranovory	205	raha
50	soko	102	mijanjy	154	ranosira	206	bosatra
51	hazo	103	mahay	155	manovarano	207	agnara
52	ala	104	mandrineke	156	vato		

7 - Betsimisaraka (Antalaha)

1	zaho	53	kobay	105	maharegny	157	alagnana
2	anao	54	voankazo	106	matahotro	158	laposiera
3	izy	55	voany	107	mandry	159	tany
4	atsika	56	raviny	108	velogno	160	rondro
5	andre	57	vahany	109	maty	161	zavogno
6	zareo	58	hoditrykakazo	110	mamono	162	lagnitry
7	ity	59	folera	111	miady	163	rivotro
8	izegny	60	ahitry	112	mangorogno	164	lanaizy
9	aketo	61	tady	113	mamopoko	165	gilasy
10	ary	62	hoditry	114	mandidy	166	hemboko
11	izovy	63	haina	115	mamaky	167	afo
12	ino	64	lio	116	manombo kiso	168	jofo
13	akaiza	65	taholagna	117	magnisiky	169	magnoro
14	afiriagna	66	vondraka	118	mangady	170	lalagna
15	manankory	67	atody	119	milomagno	171	tanety
16	aza	68	tandroko	120	magnembagna	172	mena
17	jiaby	69	rambo	121	mandeha	173	mahitso
18	maro	70	volovolo	122	avy	174	hasaka
19	vitsivitsy	71	vorondoha	123	mandry	175	fotsy
20	hely	72	loha	124	mantotry	176	mahintigny
21	hafa	73	tadigny	125	mitsangagna	177	aligny
22	araiky	74	maso	126	mihodigny	178	andro
23	aroa	75	orogno	127	lavo	179	taogno
24	telo	76	vava	128	magnamia	180	mafana
25	efatra	77	nify	129	mitagna	181	manintsy
26	dimy	78	lela	130	mangala	182	feno
27	maventy	79	angofo	131	mandrokotro	183	vaovao
28	lava	80	tongotro	132	manasa	184	antitry
29	malalaka	81	peky	133	mamitry	185	tsara
30	matevigny	82	lohalitry	134	mitifitry	186	ratsy
31	mavesatra	83	tagnana	135	manosiky	187	lo
32	hely	84	elatra	136	mitoraka	188	maloto
33	fohy	85	votraka	137	manohy	189	mahitsy
34	mahety	86	tsinay	138	manjaitry	190	boribory
35	matify	87	vozogno	139	mikonty	191	marangitry
36	viavy	88	tahezagna	140	mivolagna	192	dofoko
37	lalahy	89	tratra	141	mihira	193	malambadambatra
38	olo	90	fo	142	misoma	194	legny
39	zaza	91	aty	143	mitsilay	195	maigny
40	vady	92	migiaka	144	mivalagna	196	mety
41	vady	93	mihinagna	145	mivaingagna	197	marikitry
42	reny	94	magnekitry	146	mibontaka	198	lavitry
43	baba	95	minono	147	masova	199	ankavanagna
44	biby	96	mihaka	148	davolagna	200	ankavia
45	laoko	97	mandoa	149	lakintagna	201	agny
46	vorogno	98	mifiko	150	rano	202	agnaty
47	amboa	99	miaigny	151	oragna	203	miaraka
48	hao	100	mimoehy	152	tegnarano	204	ndraiky
49	bibilava	101	mahita	153	matsabory	205	izy ka
50	hankagna	102	mitandregny	154	ranomasigny	206	satria
51	kakazo	103	mahay	155	sira	207	agnaragna
52	atiala	104	mieritreritry	156	vato		

8 - Merina (Antananarivo)							
1	izaho	53	kibay	105	manimbolo	157	fasika
2	ianao	54	voankazo	106	matahotra	158	vovoka
3	izy	55	voa	107	matory	159	tany
4	isika	56	ravina	108	velona	160	rahona
5	ianareo	57	faka	109	maty	161	zavona
6	izy ireo	58	hodikazo	110	mamono	162	lanitra
7	ity	59	voninkazo	111	miady	163	rivotra
8	izany	60	ahitra	112	mihaza	164	ranomandry
9	eto	61	tady	113	mikapoka	165	ranomandry
10	ao	62	hoditra	114	mandidy	166	setroka
11	iza	63	hena	115	mamaky	167	afo
12	inona	64	ra	116	manatsatoka antsy	168	lavenona
13	aiza	65	taolana	117	mikiky	169	mandoro
14	oviana	66	taviny	118	mangady	170	lalana
15	ahoana	67	atody	119	milomano	171	tendrombohitra
16	tsy	68	tandroka	120	manidina	172	mena
17	rehetra	69	rambo	121	mandeha	173	maitso
18	betsaka	70	volomborona	122	avy	174	mavo
19	vitsivitsy	71	volo	123	mandry	175	fotsy
20	kely	72	loha	124	mipetraka	176	mainty
21	hafa	73	sofina	125	mitsangana	177	alina
22	iray	74	maso	126	mihodina	178	andro
23	roa	75	orona	127	mianjera	179	taona
24	telo	76	vava	128	manome	180	mafana
25	efatra	77	nify	129	mitantana	181	mangatsiaka
26	dimy	78	lela	130	manery	182	feno
27	lehibe	79	hoho	131	manakasoka	183	vaovao
28	lava	80	tongotra	132	manasa	184	antitra
29	malalaka	81	ranjo	133	mamafa	185	tsara
30	matevina	82	lohalika	134	mitifitra	186	ratsy
31	mavesatra	83	tanana	135	manosika	187	lo
32	kely	84	elatra	136	manoraka	188	maloto
33	fohy	85	kibo	137	mamehy	189	mahitsy
34	tery	86	tsinay	138	manjaitra	190	boribory
35	manify	87	vozona	139	manisa	191	maranitra
36	vehivavy	88	lamosina	140	milaza	192	dombo
37	lehilahy	89	tratra	141	mihira	193	malama
38	olona	90	fo	142	milalao	194	mando
39	zaza	91	aty	143	mitsinkafona	195	maina
40	vady	92	misotro	144	mikoriana	196	mety
41	vady	93	mihinana	145	mandry	197	akaiky
42	reny	94	manaikitra	146	mibontsina	198	lavitra
43	ray	95	mifiaka	147	masoandro	199	havanana
44	biby	96	mandrehoka	148	volana	200	havia
45	trondro	97	mandoa	149	kintana	201	any
46	vorona	98	mitsoka	150	rano	202	anaty
47	alika	99	miaina	151	orana	203	amin ny
48	hao	100	mihomehy	152	renirano	204	sy
49	bibilava	101	mahita	153	farihy	205	raha
50	kankana	102	mihaino	154	ranomasina	206	satria
51	hazo	103	mahay	155	sira	207	anarana
52	ala	104	mieritreritra	156	vato		

9 - Bara (Betroka)							
1	iaho	53	kobay	105	magnembo	157	fasy
2	henao	54	voakazo	106	matahotsy	158	bo
3	izy	55	voa	107	mandry	159	tany
4	itsika	56	ravy	108	velo	160	raho
5	henareo	57	faka	109	maty	161	zavo
6	reo	58	hodikazo	110	mamono	162	lagnitsy
7	ity	59	vonikazo	111	miady	163	tsioky
8	zay	60	akata	112	miremby	164	zavo
9	etoa	61	tady	113	mamofoky	165	ranomandry
10	ao	62	hoditry	114	manapaky	166	setroky
11	ia	63	hena	115	manilaky	167	afo
12	ino	64	lio	116	mitomboky mesa	168	lakevo
13	aia	65	tola	117	mikihy	169	magnoro
14	ombia	66	sabora	118	mihady	170	lala
15	akory	67	atody	119	milagno	171	vohitsy
16	tsy	68	tsifa	120	magnembo	172	mena
17	aby	69	ohiny	121	mandeha	173	metso
18	lako	70	volomboro	122	avy	174	vogny
19	kidikidy	71	volo	123	mandry	175	fotsy
20	kidy	72	loha	124	mitoboky	176	mety
21	hafa	73	sofy	125	mitsanga	177	hariva
22	raiky	74	maso	126	mitodiky	178	andro
23	roy	75	oro	127	lavo	179	tao
24	telo	76	vava	128	magnome	180	mafana
25	efatsy	77	nify	129	mita	181	manitsy
26	dimy	78	lela	130	mamiaky	182	atsiky
27	foloay	79	vazakoho	131	mampikasoky	183	vao
28	abo	80	tomboky	132	manasa	184	antitsy
29	malalaky	81	randro	133	mamafa	185	soa
30	matevy	82	pokopoko	134	mitifitsy	186	ratsy
31	mavesatry	83	tagna	135	mandrosy	187	lo
32	kidy	84	elatsy	136	manoraky	188	maloto
33	bory	85	troky	137	mamatotsy	189	mahitsy
34	tery	86	tsinay	138	mitrebiky	190	boribory
35	matify	87	vozo	139	magnisaky	191	maragnitsy
36	apela	88	lambosy	140	miroho	192	do
37	lilahy	89	tratra	141	miantsa	193	malama
38	olo	90	fo	142	mihira	194	mando
39	zaza	91	aty	143	mihefo	195	maiky
40	vady	92	mino	144	mandeha	196	mety
41	vady	93	homa	145	mandry	197	mariny
42	iendry	94	magnehitsy	146	mivonto	198	lavitsy
43	iaba	95	mitsetsiky	147	masoandro	199	havana
44	biby	96	mandrora	148	vola	200	havia
45	fia	97	mandoa	149	vasia	201	agny
46	voro	98	mitsioky	150	rano	202	agnaty
47	amboa	99	miay	151	ora	203	amin ny
48	hao	100	homehy	152	vavarano	204	da
49	bibilava	101	mahita	153	farihy	205	laha
50	haka	102	mahare	154	riaky	206	satria
51	hazo	103	mahay	155	sira	207	agnara
52	ala	104	mieritseritsy	156	vato		

10 - Zafisoro (Farafangana)

1	iao	53	kaboda	105	mifofogny	157	fasiky
2	anao	54	vokazo	106	matahotry	158	bo
3	izy	55	isiny	107	matory	159	tany
4	itsika	56	ravy	108	velogny	160	mika
5	anareo	57	vatry	109	afak aigny	161	zavogny
6	izy aby	58	hoditrazo	110	mamono	162	lagnitry
7	itiky	59	voninkazo	111	miady	163	rivotry
8	iza	60	hasitry	112	miremby	164	ranomandry
9	etoa	61	tady	113	mandaboky	165	ranomandry
10	amignao	62	hoditry	114	manapaky	166	teroky
11	ia	63	hena	115	mamaky	167	afo
12	ino	64	ra	116	mapitraoaky amesa	168	lakevo
13	aia	65	tola	117	mikikitry	169	magnoro
14	ovia	66	saborany	118	mangady	170	lalagny
15	magnino	67	atody	119	milomagno	171	vohitry
16	tsy	68	tandroky	120	magnembogny	172	mena
17	aby	69	ohy	121	mandeha	173	metso
18	maro	70	volomboro	122	avy	174	makamaka
19	kidikidy	71	volo	123	matory	175	fotsy
20	pitiky	72	loha	124	midoboky	176	mity
21	hafa	73	sofy	125	mitsanga	177	ariva
22	raiky	74	maso	126	mihodigny	178	andro
23	roy	75	vatagnoro	127	latsaky	179	taogny
24	telo	76	vava	128	magnome	180	may
25	efatra	77	nify	129	mitagny	181	mangatseky
26	dimy	78	lela	130	mipitsa	182	feno
27	zakabe	79	vazakoho	131	mikosiky	183	vao
28	lava	80	tomboky	132	manasa	184	mavozo
29	malalaky	81	randro	133	mamafa	185	tsara
30	matevy	82	laditry	134	mitifitry	186	ratsy
31	mavesatry	83	tagna	135	manolaky	187	mavao
32	pitiky	84	elatry	136	mitoraky	188	maloto
33	botry	85	troky	137	mamatotry	189	mahitsy
34	pitsa	86	oliky	138	manjaitry	190	boribory
35	manify	87	vozo	139	magnisa	191	maragnitry
36	viavy	88	lamosy	140	mizaka	192	bofoky
37	lela	89	tratra	141	mihira	193	malama
38	olo	90	fo	142	mihira	194	lendegny
39	marao	91	aty	143	mitsingevaheva	195	maigny
40	vady	92	mindrano	144	mandeha	196	izay
41	vady	93	minagny	145	mandry	197	akeky
42	endry	94	magnekitry	146	mivonto	198	lavitra
43	aba	95	mameky	147	masoandro	199	akavana
44	biby	96	mandoha rehoky	148	volagny	200	akavia
45	laokandrano	97	mandoa	149	vasia	201	agny
46	voro	98	misotry	150	rano	202	agnaty
47	amboa	99	miaigny	151	erikandro	203	amin ny
48	hao	100	mihehy	152	renirano	204	sy
49	bibilava	101	mahita	153	ranodobo	205	ra
50	aka	102	miteno	154	ranomasy	206	satria
51	hazo	103	mahalala	155	sira	207	agnara
52	ala	104	mandiniky	156	vato		

11 - Betsimisaraka (Fenoarivo-Est)							
1	zaho	53	vely	105	mioroko	157	fasiky
2	ana	54	vihindraha	106	matahotro	158	vovoko
3	izy	55	ambeko	107	mandry	159	tany
4	antsika	56	ravindraha	108	velogno	160	rahogno
5	anare	57	vahiny	109	maty	161	zavogno
6	zare	58	hodikakazo	110	mamono	162	lagnitry
7	ito	59	folera	111	miady	163	rivotro
8	irohony	60	ahitry	112	mihaza	164	gilasy
9	aketo	61	afotro	113	mamely	165	gilasy
10	aka	62	hoditry	114	manapaka	166	emboko
11	izovy	63	ambomaty	115	mamaky	167	afo
12	ino	64	ra	116	manombo kiso	168	jofo
13	aiza	65	tolagna	117	magnisiky	169	magnoro
14	oviagna	66	matavy	118	mangady	170	lalagna
15	karakory	67	atody	119	milomagno	171	tanety
16	tsy	68	tandroko	120	magnembana	172	mena
17	jiaby	69	volombody	121	mandeha	173	maitso
18	fontry	70	volovolomborogno	122	havy	174	mavo
19	vitsivitsy	71	vorondoha	123	mandry	175	fotsy
20	hely	72	loha	124	mantotry	176	maintigny
21	hafa	73	tadigny	125	mitsangagna	177	androaligny
22	araiky	74	maso	126	mihodigny	178	andro
23	aro	75	orogno	127	lavo	179	tono
24	telo	76	vava	128	magnamia	180	mafana
25	efatra	77	nify	129	mitantagna	181	mangatsiaka
26	dimy	78	lela	130	malaka	182	feno
27	maventy	79	angofo	131	mampikasiky	183	vaovao
28	lava	80	hongotro	132	manasa	184	antitry
29	malalaka	81	vavitsy	133	mamafa	185	tsara
30	matevigny	82	lohalitry	134	mitifitry	186	ratsy
31	mavesatra	83	tagnagna	135	manosiky	187	lo
32	hely	84	elatra	136	manoraka	188	maloto
33	fohy	85	kibo	137	mamehy	189	mahitsy
34	mahety	86	tsinay	138	manjaitry	190	boribory
35	manify	87	tenda	139	magnisa	191	marangitry
36	viavy	88	tahezagna	140	mivolagna	192	malomogno
37	lalahy	89	tratra	141	mihira	193	malamatra
38	ologno	90	fo	142	midola	194	boka
39	zaza	91	aty	143	mitsingevagna	195	maigny
40	vady	92	mino	144	magnoriky	196	manjary
41	vady	93	homagna	145	mandry	197	mariny
42	niny	94	magnekitry	146	mibontsigny	198	lavitry
43	baba	95	minono	147	masoandro	199	ankavanagna
44	biby	96	mihaka	148	davolagna	200	ankavia
45	loko	97	mandoa	149	kintagna	201	agny
46	vorogno	98	mitsotro	150	rano	202	agnatiny
47	kiva	99	miaigny	151	oragnandro	203	miaraka
48	ho	100	mimoehy	152	renirano	204	sy
49	bibilava	101	mahita	153	dobo	205	raha
50	hankagna	102	mitandregny	154	ranomasigny	206	satria
51	kakazo	103	mahe	155	sira	207	agnaragna
52	atiala	104	mieritreritry	156	vato		

12 - Betsileo (Fianarantsoa)							
1	aho	53	tehigna	105	mifofona	157	fasika
2	agnao	54	voankazo	106	matahotsa	158	vovoka
3	i	55	voa	107	matory	159	tany
4	tsika	56	ravina	108	velogna	160	rahogna
5	agnareo	57	fakany	109	maty	161	zavona
6	reo	58	hodikazo	110	mamono	162	lagnitsa
7	itito	59	voninkazo	111	miady	163	rivotsa
8	zany	60	ahitsa	112	miagnala	164	ranomandry
9	eto	61	tady	113	mikapoka	165	ranomandry
10	ao	62	hoditsa	114	mandidy	166	setroka
11	ia	63	hena	115	mamaky	167	afo
12	ino	64	ra	116	manatsatoka antsy	168	lavenona
13	aia	65	taolagna	117	mikiky	169	magnoro
14	oviana	66	taviny	118	mangady	170	lalagna
15	ahoana	67	atody	119	milomagno	171	tendrobohitsa
16	tsa	68	tandroka	120	mitsidina	172	mena
17	aby	69	rambo	121	mandeha	173	maintso
18	be	70	volomborona	122	avy	174	mavo
19	mahalagna	71	volondoha	123	mandrimandry	175	fotsy
20	kely	72	loha	124	mitoatsa	176	mainty
21	hafa	73	sofina	125	mitsangana	177	aligna
22	raika	74	maso	126	mihodina	178	andro
23	roa	75	orogna	127	migetraka	179	taolagna
24	telo	76	vava	128	manome	180	mafana
25	efatsa	77	nify	129	mitantagna	181	manara
26	dimy	78	lela	130	mitery	182	feno
27	ngeza	79	hoho	131	mikiky	183	vaovao
28	lava	80	tongotsa	132	manasa	184	antitsa
29	malalaka	81	ranjo	133	mamafa	185	soa
30	matevigna	82	lohalika	134	mitifitsa	186	ratsy
31	mavesatsa	83	tagnana	135	manosika	187	lo
32	kely	84	elatsa	136	mitoraka	188	maloto
33	bory	85	troka	137	mamehy	189	mahitsy
34	tery	86	tsinay	138	manjaitra	190	boribory
35	manify	87	vozogna	139	magnisa	191	maragnitsa
36	ampela	88	lamosigna	140	mitarogna	192	dombo
37	lehilahy	89	tratra	141	mihira	193	malama
38	olo	90	fo	142	miregnina	194	mando
39	kilonga	91	aty	143	mitsinkafona	195	maigna
40	vady	92	misotro	144	mitete	196	mety
41	vady	93	mihinana	145	mandry	197	mariny
42	reny	94	magnehitsa	146	mibontsina	198	lavitsa
43	ray	95	minono	147	masoandro	199	havana
44	biby	96	mandrehoka	148	volagna	200	havia
45	trondro	97	mandoa	149	kintagna	201	agny
46	vorogna	98	mitsoka	150	rano	202	agnaty
47	amboa	99	miaigna	151	oragna	203	amin ny
48	hao	100	mihehy	152	renirano	204	sy
49	bibilava	101	mahita	153	farihy	205	lehe
50	kankana	102	mitaino	154	ranomasina	206	satria
51	hazo	103	mahay	155	sira	207	agnarana
52	ala	104	mieritseritsa	156	vato		

13 - Betsimisaraka (Mahanoro)							
1	zaho	53	fioka	105	miorogna	157	fasika
2	ano	54	vonkanzo	106	vaka	158	vovoka
3	izy	55	vihy	107	mandry	159	fotaka
4	antsena	56	ravina	108	velogno	160	rahona
5	andreo	57	vahatra	109	diso	161	zavona
6	zareo	58	oditra kakazo	110	mamono	162	lagnitra
7	itogna	59	folera	111	miady	163	rivotra
8	indrogna	60	ahitra	112	miaza	164	avandra
9	aketo	61	tady	113	mamango	165	ranomandry
10	ako	62	oditra	114	manapaka	166	mifoka
11	zovy	63	hena	115	mizara	167	afo
12	ino	64	ra	116	manomboka antsy	168	lavenona
13	akeza	65	tolagna	117	mandoditra	169	mandoro
14	oviana	66	menagny	118	angady	170	lalana
15	karakory	67	atody	119	mandagno	171	tavirana
16	tsy	68	tandroka	120	mirigna	172	mena
17	ziaby	69	volombody	121	mandeha	173	meso
18	fatatra	70	volomborogna	122	tonga	174	mavo
19	visivisy	71	randrana	123	mandry	175	fosy
20	bitika	72	loha	124	mipetraka	176	mintina
21	afa	73	tadigny	125	mitsangana	177	alina
22	reka	74	maso	126	miodigna	178	andro
23	roa	75	orona	127	lavo	179	tona
24	telo	76	vava	128	magnomia	180	mafana
25	efatra	77	nify	129	mitantana	181	mangasiaka
26	dimy	78	lela	130	mamia	182	feno
27	maventy	79	angofo	131	mikosoka	183	vo
28	lava	80	tongotra	132	manasa	184	antitra
29	matahitra	81	vovisy	133	mamafa	185	sara
30	matevina	82	lohalitra	134	mitifitra	186	rasy
31	mavesatra	83	tagnana	135	manosika	187	lo
32	bitika	84	elatra	136	mambalavala	188	maloto
33	fohika	85	kibo	137	mamatotra	189	mahisy
34	tery	86	tsine	138	manjetra	190	boribory
35	manify	87	ambozogna	139	magnisa	191	marangitra
36	viavy	88	tombokoka	140	mizaka	192	malombona
37	lilahy	89	tratra	141	mihira	193	malama
38	olona	90	fo	142	milalo	194	mando
39	zamadinika	91	aty	143	miempo	195	megna
40	vady	92	midroka	144	mandeha	196	aminanzy
41	vady	93	mihinana	145	mandry	197	marivo
42	mama	94	magnekitra	146	mivonto	198	lavitra
43	baba	95	minono	147	masoandro	199	ankavanana
44	biby	96	mandreoka	148	volana	200	avia
45	laoka	97	mandoa	149	kintana	201	agny
46	vorogna	98	misotra	150	rano	202	agnaty
47	kiva	99	miegna	151	oranandro	203	miaraka amin ny
48	ao	100	mimehy	152	siragnana	204	ombanin ny
49	bibilava	101	mahita	153	farihy	205	raha
50	viky	102	miteno	154	ranomasigna	206	satria
51	kakazo	103	mahe	155	sira	207	agnaragna
52	atiala	104	mieritreritra	156	vato		

14 - Sakalava (Maintirano)

1	zaho	53	kobay	105	magnimbo	157	fasiky
2	iha	54	voankazo	106	matahotsy	158	lemboky
3	i	55	masomboly	107	miroro	159	tany
4	tsika	56	ravy	108	velo	160	raho
5	nareo	57	vahitry	109	maty	161	zavo
6	reo	58	holikazo	110	mamono	162	lagnitry
7	ity	59	folera	111	mialy	163	tsioky
8	iriky	60	akata	112	mihaza	164	lagilasy
9	eto	61	taly	113	mamango	165	glasy
10	ao	62	holitry	114	mandily	166	setroky
11	ia	63	hena	115	mamaky	167	motro
12	ino	64	lio	116	mitomboky meso	168	lavenoky
13	aia	65	taola	117	mihaotsy	169	magnoro
14	ombia	66	vondraky	118	mangaly	170	lala
15	nahoa	67	atoly	119	milagno	171	vohitse
16	tsy	68	tandroka	120	magnidina	172	mena
17	iaby	69	hohy	121	mandeha	173	maitso
18	maro	70	volomboro	122	bakagny	174	vogny
19	kelikely	71	volo	123	miroro	175	foty
20	kely	72	loha	124	mipetraka	176	mainty
21	hafa	73	sofy	125	mitsotaky	177	aliky
22	raiky	74	maso	126	mioliky	178	andro
23	roy	75	oro	127	lavo	179	tao
24	telo	76	vava	128	magnome	180	may
25	efatsy	77	ihy	129	mita	181	magnitsy
26	dimy	78	lela	130	mandray	182	feno
27	bevata	79	angofo	131	mitakosiky	183	vao
28	habo	80	tongotsy	132	manasa	184	antitsy
29	malalaky	81	randro	133	mamasa	185	soa
30	matevy	82	lohaliky	134	mitifitsy	186	raty
31	masatsy	83	tagna	135	mandonjy	187	mavay
32	kely	84	elatsy	136	mitoraky	188	maloto
33	boribory	85	kibo	137	mamatotsy	189	mahity
34	maletsy	86	tinay	138	manjaitsy	190	boriboriky
35	manify	87	akaiky	139	magnisa	191	maragnitsy
36	ampela	88	lambosy	140	mivola	192	madomo
37	lelahy	89	tratra	141	miantsa	193	madojo
38	olo	90	fo	142	miisa	194	boboky
39	aja	91	aty	143	mihafo	195	maiky
40	valy	92	mino	144	mikorisa	196	sabona
41	valy	93	mihina	145	mandriky	197	akaiky
42	neny	94	manaikitsy	146	mivonto	198	lavitsy
43	baba	95	mitsintsiky	147	masoandro	199	kavana
44	biby	96	mandreoky	148	boara	200	havia
45	fia	97	mandoa	149	basia	201	agny
46	voro	98	mitsoky	150	rano	202	agnaty
47	amboa	99	miay	151	ora	203	amin ny
48	hao	100	mihehy	152	ranomandeha	204	sy
49	bibilava	101	mijilo	153	ranovory	205	raha
50	hanka	102	mijanjy	154	ranomasy	206	satria
51	hazo	103	mahay	155	sira	207	agnara
52	ala	104	mieritreritry	156	vato		

15 - Sakalava (Mahajanga)							
1	zaho	53	kibay	105	mahatsapa	157	fasika
2	anao	54	voankakazo	106	mavozo	158	vovoka
3	izy	55	voa	107	matory	159	tany
4	tsika	56	feliky	108	velo	160	rahona
5	ndreo	57	fotony	109	naty	161	zavona
6	reo	58	hodinkakazo	110	mamono	162	lagnitra
7	ndreto	59	folera	111	miady	163	rivotra
8	igny	60	katsakatsa	112	mitsakaraka	164	ranomandry
9	eto	61	tady	113	mamango	165	laglasy
10	ao	62	hoditra	114	manapaka	166	setroka
11	iha	63	hena	115	mamaky	167	motro
12	karakory	64	lio	116	manatsatoka meso	168	lavenona
13	ahia	65	taolana	117	mikiky	169	may
14	ombia	66	jabora	118	mangady	170	lalagna
15	karakory	67	atody	119	milomano	171	bongo
16	tsy	68	vombony	120	mitily	172	mena
17	jiaby	69	ohy	121	mandeha	173	maitso
18	maro	70	volomboro	122	boaka	174	mavo
19	sasatsasany	71	fagneva	123	mandry	175	fotsy
20	bitika	72	loha	124	mipetraka	176	manity
21	hafa	73	sofy	125	mitsangana	177	matonaly
22	raiky	74	maso	126	mihodina	178	andro
23	roa	75	orona	127	lavo	179	taona
24	telo	76	vava	128	manome	180	mafana
25	efatra	77	nify	129	mitazona	181	manintsy
26	dimy	78	lela	130	mipositra	182	feno
27	maventy	79	hangofo	131	mikasoka	183	kabary
28	lava	80	tongotra	132	manasa	184	matohy
29	malalaka	81	ranjo	133	mamafa	185	tsara
30	matevina	82	lohalika	134	mitifitra	186	ratsy
31	mavesatra	83	tanana	135	manosika	187	lozitra
32	balitika	84	elatra	136	manoraka	188	maloto
33	fohy	85	kibo	137	mamatotra	189	mahitsy
34	tery	86	marombo	138	mitrebiky	190	boribory
35	mahia	87	vozona	139	mikonty	191	maranitra
36	manangy	88	lambosy	140	mivola	192	dombo
37	lehilahy	89	tratra	141	mihira	193	malamatra
38	olo	90	fo	142	misoma	194	lena
39	tsaiky	91	aty	143	mitsingevana	195	maiky
40	vady	92	migiaka	144	misosa	196	mety
41	vady	93	mihina	145	mandrika	197	marikitra
42	mama	94	manaikitry	146	mibontsina	198	lavitra
43	baba	95	misintogna	147	zova	199	havanagna
44	biby	96	mandrehoka	148	volana	200	havia
45	filao	97	mandoa	149	kintana	201	a
46	vorona	98	milefa	150	rano	202	anatiny
47	amboa	99	mifoka	151	orana	203	miaraka
48	hao	100	mitokiky	152	renirano	204	ndreky
49	bibilava	101	mizaha	153	matsabory	205	izy koa
50	kankana	102	mitandregny	154	ranosira	206	satria
51	kakazo	103	mahay	155	sira	207	anarana
52	ala	104	mieritreritra	156	vato		

16 - Antaimoro (Manakara)

1	iaho	53	salohy	105	mifofona	157	fasika
2	anao	54	vokazo	106	matahotra	158	vovoka
3	izy	55	voa	107	mandry	159	tany
4	isikana	56	ravigna	108	velogna	160	rahogna
5	indreo	57	fakany	109	maty	161	zavogna
6	indreo aby	58	hodikazo	110	mamono	162	lagnitra
7	ity	59	vonikazo	111	miady	163	rivotry
8	iry	60	ahitra	112	miremby	164	ranomangatseka
9	eto	61	tady	113	mamango	165	ranomandry
10	ao	62	hoditra	114	manampaka	166	setroka
11	iza	63	hena	115	magnilaky	167	afo
12	ino	64	ra	116	mamira antsy	168	lakevogna
13	aia	65	tolagna	117	mikisiky	169	magnoro
14	ovena	66	vondraky	118	mangady	170	lalagna
15	ahoa	67	atody	119	milomagno	171	vohitry
16	tsy	68	tandroka	120	magnembogna	172	mena
17	aby	69	rambognany	121	mandeha	173	metso
18	maro	70	volomborogny	122	avy	174	mavo
19	kidikidiky	71	volo	123	mandry	175	fotsy
20	madinika	72	loha	124	midoboky	176	menty
21	hafa	73	tadigny	125	mitsanga	177	aligny
22	raiky	74	maso	126	mihodigna	178	andro
23	roy	75	orogny	127	lavo	179	taogna
24	telo	76	vava	128	magnome	180	mafana
25	efatra	77	nify	129	mitagna	181	mangatseky
26	dimy	78	lela	130	mipitsa	182	feno
27	zakabe	79	vazakoho	131	mikasika	183	vao
28	lava	80	tongotra	132	manasa	184	antitry
29	belapaka	81	vovitsy	133	mamafa	185	tsara
30	matevigna	82	sipoko	134	mitifitry	186	ratsy
31	mavesatra	83	tagnana	135	manosiky	187	lo
32	madinika	84	elatry	136	magnary	188	maloto
33	fohika	85	kibo	137	mamatotra	189	mahitsy
34	tery	86	oliky	138	manjaitra	190	bory
35	manify	87	vozogny	139	magnisa	191	maragnitra
36	viavy	88	lamosigny	140	mizaka	192	bofogna
37	lelahy	89	tratra	141	mihira	193	malama
38	olo	90	fo	142	mihira	194	mando
39	zaza	91	aty	143	miempo	195	maigna
40	vady	92	minogny	144	milentika	196	aminazy
41	vady	93	mihinagna	145	mivengagny	197	akeky
42	iendry	94	magnekitra	146	mibotsigna	198	lavitra
43	iaba	95	misesitra	147	masoandro	199	havanana
44	biby	96	mandrehoka	148	volagna	200	havia
45	laokandrano	97	mandoa	149	kitagna	201	agny
46	vorogna	98	mitsioka	150	rano	202	agnatiny
47	amboa	99	miaigna	151	oronkandro	203	amin ny
48	hao	100	mihehy	152	renirano	204	da
49	bibilava	101	mahita	153	takebo	205	raha
50	hankana	102	miteno	154	ranomasigna	206	satria
51	hazo	103	mahay	155	sira	207	agnara
52	ala	104	mandignika	156	vato		

17 - Antambohoaka (Mananjary)

1	iao	53	kaboda	105	mifofona	157	fasina
2	anao	54	voanakakazo	106	mataotra	158	vovoka
3	izy	55	vo	107	mandry	159	tany
4	antsena	56	ravina	108	velogna	160	rahogna
5	anareo	57	vahany	109	maty	161	zavogna
6	arizareo	58	hodinakakazo	110	mamono	162	lagnitra
7	ity	59	folera	111	miady	163	agnina
8	iry	60	ahitra	112	mambely	164	ranomandry
9	eto	61	tady	113	mandraboka	165	ranomandry
10	ao	62	hoditra	114	manapaky	166	zavogna
11	iza	63	hena	115	mamaky	167	afo
12	ino	64	ra	116	manitroboka kiso	168	lavenona
13	aza	65	tolagna	117	mikiky	169	magnoro
14	ovena	66	matavy	118	mangady	170	lalana
15	akory	67	atody	119	mandagno	171	vohitra
16	tsy	68	tandroka	120	miborigna	172	mena
17	izy marobe	69	volombody	121	mandeha	173	metso
18	maro	70	volona vorogna	122	avy	174	mavo
19	vitsivitsy	71	randrana	123	mandry	175	fotsy
20	bitaka	72	loha	124	mipetraka	176	minty
21	hafa	73	tadigny	125	mitsangana	177	hariva
22	raika	74	maso	126	mihodigna	178	andro
23	roa	75	orogna	127	lavo	179	taona
24	telo	76	vava	128	magnome	180	mafana
25	efatra	77	nify	129	mitana	181	mangatsika
26	dimy	78	lela	130	mamoritra	182	feno
27	agnana be	79	vanjakoho	131	mampikasika	183	vao
28	lava	80	tongotra	132	manasa	184	antitra
29	malalaky	81	randro	133	mamafa	185	tsara
30	matevina	82	lohalitra	134	mitifitra	186	ratsy
31	mavesatra	83	tagnana	135	manosika	187	bobongolo
32	bitika	84	elatra	136	mamalavala	188	maloto
33	fohika	85	kibo	137	mamatotra	189	mahitsy
34	tery	86	tsikolika	138	manjaitra	190	boribory
35	manify	87	vozogna	139	magnisa	191	maranitra
36	viavy	88	lamosina	140	mizaka	192	dombo
37	lilahy	89	tratra	141	mihira	193	malama
38	olona	90	fo	142	milalao	194	mando
39	zaza	91	aty	143	mitsingevaheva	195	maigna
40	vady	92	misotro	144	mikorisa	196	mety
41	vady	93	mihinana	145	mandry	197	marivo
42	endry	94	magnekitra	146	mivonto	198	lavitra
43	iaba	95	mitsisitra	147	masoandro	199	havanana
44	biby	96	mandrehoka	148	vola	200	havia
45	laoka	97	mandoa	149	kintana	201	agny
46	vorogna	98	mitsotra	150	rano	202	agnaty
47	amboa	99	miaigna	151	orana	203	amin ny
48	hao	100	mihomehy	152	renirano	204	de
49	bibilava	101	mahita	153	kanaly	205	raha
50	viky	102	miteno	154	ranomasina	206	satria
51	kakazo	103	mahay	155	sira	207	agnarana
52	atiala	104	mieritreritra	156	vato		

18 - Tsimihety (Mandritsara)							
1	zaho	53	koboay	105	maharegny	157	alagnagna
2	anao	54	voankazo	106	matahotro	158	jofompotaka
3	izy	55	ambe	107	mandry	159	tany
4	atsika	56	raviny	108	velogno	160	rondro
5	areo	57	vahany	109	maty	161	zavogno
6	zareo	58	hodikakazo	110	mamono	162	lagnitry
7	toy	59	felagna	111	miady	163	rivotro
8	zany	60	ahitry	112	mangorogno	164	fanala
9	aketo	61	tady	113	mamboko	165	lagilasy
10	aroy	62	hoditry	114	mandidy	166	tsemboko
11	azovy	63	hena	115	mamaky	167	motro
12	ino	64	lio	116	mitomboko	168	jofo
13	aiza	65	taholagna	117	magniky	169	magnoro
14	afiriagna	66	vondraka	118	mangady	170	lalagna
15	manankory	67	atody	119	milomagno	171	tanety
16	aza	68	ampondo	120	magnembagna	172	mena
17	jiaby	69	ohy	121	mandeha	173	mahitso
18	maro	70	volomborogno	122	havy	174	fondragna
19	tsy fontry	71	maramaragna	123	mandry	175	fotsy
20	hely	72	loha	124	mantotry	176	mahintigny
21	hafa	73	sofigny	125	mitsangagna	177	haligny
22	araiky	74	maso	126	mihodigny	178	andro
23	aroa	75	horogno	127	lavo	179	tagno
24	telo	76	vava	128	magname	180	mafana
25	aifatra	77	nify	129	mitagna	181	manintsy
26	dimy	78	lela	130	mangoeky	182	feno
27	geda	79	angofo	131	mandrokotro	183	vaovao
28	lava	80	hongotro	132	manasa	184	antitry
29	malalaka	81	vavitsy	133	mamafa	185	tsara
30	matevigny	82	lohalina	134	mitifitry	186	ratsy
31	mavesatra	83	tagnagna	135	manidigny	187	lo
32	hely	84	embagna	136	manopy	188	maloto
33	fohy	85	boko	137	mamehy	189	mahitsy
34	mahety	86	tsinay	138	manjaitry	190	boribory
35	matify	87	ambozogno	139	magnisa	191	maragnitry
36	vaiavy	88	tahezagna	140	mivolagna	192	malomogno
37	lalahy	89	tratra	141	mihira	193	malamatra
38	ologno	90	fo	142	misoma	194	lendegny
39	zaza	91	aty	143	mitsilay	195	maigny
40	vady	92	migiaka	144	mivalagna	196	manjary
41	vady	93	homagna	145	mivongagna	197	mariny
42	niny	94	magnekitry	146	mivonto	198	lavitry
43	baba	95	minono	147	masova	199	ankahery
44	biby	96	mihaka	148	davolagna	200	ankavia
45	laoko	97	mandoa	149	lakintagna	201	akagny
46	vorogno	98	mitsotro	150	rano	202	agnatiny
47	amboa	99	miaigny	151	mahalegny	203	amin ny
48	hao	100	mimoehy	152	tegnarano	204	sindraiky
49	bibilava	101	mahita	153	matsabory	205	raohatra
50	hankagna	102	mitandregny	154	ranomasigny	206	satria
51	kakazo	103	mahay	155	sira	207	agnaragna
52	atiala	104	mieritreritry	156	vato		

19 - Masikoro (Miary)

1	zaho	53	kobay	105	magnibo	157	fasy
2	iha	54	voankazo	106	matahotsy	158	lemboky
3	ie	55	vihy	107	miroro	159	tany
4	tsika	56	ravi	108	velo	160	hiboky
5	nareo	57	fototsy	109	maty	161	zavo
6	rozy	58	holinkazo	110	mamono	162	lagnitsy
7	toy	59	folera	111	mialy	163	tsioky
8	eroy	60	akata	112	maminta	164	ranomihandro
9	etoa	61	taly	113	mamango	165	vongandrano
10	ao	62	holitsy	114	manapaky	166	setroky
11	ia	63	hena	115	mamaky	167	bolo
12	ino	64	lio	116	mitomboky meso	168	lavenoky
13	aia	65	taola	117	magnihy	169	magnoro
14	mbia	66	vondraky	118	mihaly	170	lala
15	manao ahoa	67	atoly	119	milagno	171	vohitsy
16	tsy	68	tsifa	120	mitily	172	mena
17	iaby	69	ohy	121	mandeha	173	maintso
18	maro	70	volomboro	122	avy	174	vogny
19	tsy ampiampy	71	volo	123	miroro	175	foty
20	kelikely	72	kabeso	124	mipetraky	176	mainty
21	hafa	73	sofy	125	mitsanga	177	haliky
22	raiky	74	maso	126	miodiky	178	anjo
23	roe	75	oro	127	lavo	179	tao
24	telo	76	vava	128	magnome	180	mafana
25	efatse	77	hy	129	manday	181	manintsy
26	limy	78	lela	130	mandrambe	182	atsiky
27	bevata	79	hoho	131	mikosoke	183	vao
28	lava	80	tomboky	132	manasa	184	antitsy
29	malalaky	81	ranjo	133	mamafa	185	soa
30	matevy	82	ongotsy	134	mitifitsy	186	raty
31	mavesatsy	83	tagna	135	mandrosy	187	mantsy
32	maliniky	84	helatsy	136	mitoraky	188	maloto
33	boribory	85	troky	137	mamehy	189	mahity
34	maifitsy	86	tinay	138	mitsebiky	190	boribory
35	matify	87	vozo	139	magnisaky	191	maragnitsy
36	ampela	88	lambosy	140	mivola	192	madomoky
37	johary	89	tratra	141	miantsa	193	madoso
38	olo	90	fo	142	misa	194	lende
39	aja	91	aty	143	mitsangevaheva	195	maiky
40	valy	92	mino	144	mitsororoky	196	mety
41	valy	93	mihina	145	mandriky	197	mariniky
42	reny	94	magnehitsy	146	mibongo	198	lavitsy
43	baba	95	mintsintsiky	147	masoandro	199	ankavana
44	biby	96	mandrehoky	148	vola	200	ankavia
45	fia	97	mandoa	149	basia	201	agny
46	voro	98	mitsioky	150	rano	202	agnaty
47	alika	99	miay	151	ora	203	amin ny
48	hao	100	mihehy	152	vavarano	204	sy
49	bibilava	101	mahita	153	kitoboke	205	laha
50	soko	102	mijanjy	154	riake	206	satria
51	hazo	103	mahay	155	sira	207	agnara
52	ala	104	mandiniky	156	vato		

20 - Sakalava (Morondava)

1	zaho	53	kobay	105	magnimbo	157	fasiky
2	iha	54	vihinkazo	106	mavozo	158	vovoky
3	ie	55	vihiny	107	miroro	159	tany
4	tsika	56	raviny	108	velo	160	hiboky
5	reo	57	vahany	109	maty	161	zavo
6	rozy	58	holinkazo	110	mamono	162	lagnitsy
7	toy	59	folera	111	mialy	163	tsioky
8	zay	60	akata	112	mihaza	164	havandra
9	eto	61	hosy	113	mamango	165	havandra
10	ao	62	holitsy	114	manapaky	166	setroky
11	ia	63	hena	115	mizara	167	motro
12	ino	64	lio	116	mitomboky meso	168	lavenoky
13	aia	65	taola	117	magnihy	169	magnoro
14	ombia	66	vondraky	118	mihaly	170	lala
15	akory	67	atoly	119	milagno	171	vohitsy
16	tsy	68	tandroky	120	mitily	172	mena
17	iaby	69	hohy	121	mandeha	173	maitso
18	maro	70	volomboro	122	avy	174	vogny
19	tsiampiampy	71	volo	123	miroro	175	foty
20	tsiampy	72	loha	124	mitoboky	176	mainty
21	hafa	73	sofy	125	mitsanga	177	matognaly
22	raiky	74	maso	126	mihodiky	178	andro
23	roe	75	oro	127	miantonta	179	tao
24	telo	76	vava	128	magnome	180	may
25	efatsy	77	hy	129	mitazo	181	manintsy
26	dimy	78	lela	130	mipiritsy	182	atsiky
27	bevata	79	hoho	131	mikosiky	183	vao
28	lava	80	tomboky	132	manasa	184	antitsy
29	malalaky	81	ranjo	133	mamotsy	185	soa
30	matevy	82	lohaliky	134	mitifitsy	186	raty
31	mavesatsy	83	tagna	135	manosiky	187	mantsy
32	kelikely	84	elatsy	136	mitoraky	188	maloto
33	boribory	85	sarotro	137	mamehy	189	mahity
34	maifitsy	86	tinay	138	mitrebiky	190	boriboriky
35	matify	87	vozo	139	magnisaky	191	maragnitsy
36	ampela	88	lambosy	140	magnambara	192	dombo
37	johary	89	tratra	141	miantsa	193	madoso
38	olo	90	fo	142	misa	194	le
39	aja	91	aty	143	mihafo	195	maiky
40	valy	92	mino	144	mivavatsy	196	mahity
41	valy	93	homa	145	mandrike	197	mariniky
42	neny	94	magnehitse	146	mivonto	198	lavitsy
43	baba	95	minono	147	masoandro	199	havana
44	biby	96	mandrehoky	148	vola	200	havia
45	fia	97	mandoa	149	basia	201	agny
46	voro	98	mifioky	150	rano	202	agnaty
47	amboa	99	miay	151	ora	203	amin ny
48	hao	100	mihehy	152	vavarano	204	noho
49	bibilava	101	manenty	153	dobo	205	laha
50	hanka	102	mijanjy	154	ranomasy	206	satria
51	hazo	103	mahay	155	sira	207	agnara
52	ala	104	mametsivetsy	156	vato		

21 - Antanosy (Tolagnaro)							
1	izaho	53	angira	105	magnatsona	157	fasy
2	hanao	54	voankazo	106	matahosy	158	lemboky
3	izy	55	vihy	107	matory	159	tany
4	sika	56	raviny	108	velo	160	raho
5	handreo	57	vahatsy	109	maty	161	zavo
6	lahireo	58	holikazo	110	mamono	162	lagnisy
7	ty	59	voninkazo	111	miady	163	rivosy
8	iriho	60	ahitsy	112	mamita	164	fanala
9	etaho	61	tady	113	manjera	165	ranomivonga
10	ao	62	hoditry	114	manapaky	166	setroky
11	ia	63	hena	115	mizara	167	afo
12	ino	64	lio	116	mitomboky mesa	168	lavenoky
13	aia	65	taola	117	mikihy	169	magnoro
14	ombia	66	vondraky	118	mihaly	170	lala
15	miahy akory	67	atody	119	milomagno	171	vohisy
16	tsy	68	tsifa	120	mitily	172	mena
17	iereo aby	69	ohiny	121	mandeha	173	menso
18	maro	70	volomboro	122	avy	174	vogny
19	kidikidy	71	volo	123	mandry	175	fosy
20	kidy	72	loha	124	mitoboky	176	menty
21	hafa	73	sofy	125	misanga	177	hariva
22	raiky	74	maso	126	mihodiky	178	andro
23	ro	75	oro	127	lavo	179	tao
24	telo	76	vava	128	magnome	180	mafana
25	efatry	77	nify	129	mita	181	manara
26	dimy	78	lela	130	mandramby	182	feno
27	foloay	79	hoho	131	migosa	183	vaovao
28	lava	80	tomboky	132	manasa	184	antisy
29	malaladalaky	81	ranjo	133	mamafa	185	soa
30	matevy	82	pokopoko	134	mitifisy	186	rasy
31	mavesatry	83	tagna	135	manosiky	187	mavay
32	kidy	84	elatsy	136	manoraky	188	maloto
33	fohy	85	troky	137	mamatosy	189	mahisy
34	tery	86	sinay	138	manzaisy	190	boribory
35	mahia	87	vozo	139	magnisaky	191	maragnisy
36	ampela	88	lambosy	140	magnambara	192	domoky
37	lelahy	89	tratra	141	miantsa	193	malama
38	olo	90	fo	142	mihira	194	mando
39	zaza	91	aty	143	mitsinkafo	195	maiky
40	vady	92	mino	144	mikororoky	196	mety
41	vady	93	mihina	145	mandry	197	mariny
42	endry	94	manifasy	146	mibonsy	198	lavisy
43	ra	95	mitsintsiky	147	masoandro	199	havana
44	biby	96	mandrehoky	148	vola	200	havia
45	fia	97	mandoa	149	kinta	201	agny
46	voro	98	mifioky	150	rano	202	agnatiny
47	amboa	99	miay	151	ora	203	amin gny
48	hao	100	mihehy	152	renirano	204	da
49	bibilava	101	mahita	153	farihy	205	no
50	soko	102	miheno	154	riaky	206	satria
51	hazo	103	mahay	155	sira	207	agnara
52	ala	104	mieriserisy	156	vato		

22 - Vezo (Toliara)

1	zaho	53	kobay	105	manimbo	157	fasy
2	iha	54	voankazo	106	matahotse	158	lemboke
3	ie	55	voa	107	miroro	159	tany
4	tsika	56	ravy	108	velo	160	hiboke
5	nareo	57	fototse	109	mate	161	zavo
6	rozy	58	holinkazo	110	mamono	162	lanitse
7	toy	59	folera	111	mialy	163	tsioke
8	zay	60	akata	112	mihaza	164	ranomivonga
9	etoa	61	taly	113	mamango	165	vongandrano
10	ao	62	holitse	114	manapake	166	setroke
11	ia	63	hena	115	mamake	167	afo
12	ino	64	lio	116	mitomboke meso	168	lavenoke
13	aia	65	taola	117	mikihy	169	tsofora
14	ombia	66	vonjake	118	mihaly	170	lala
15	akory	67	atoly	119	milano	171	vohitse
16	tsy	68	tsifa	120	mitily	172	mena
17	iaby	69	hohy	121	mandeha	173	maintso
18	maro	70	volomboro	122	avy	174	mavo
19	tsiampeampe	71	volo	123	miroro	175	foty
20	tsiampe	72	loha	124	mitoboke	176	mainte
21	hafa	73	sofy	125	mitsanga	177	haly
22	raike	74	maso	126	mihodike	178	andro
23	roe	75	oro	127	tonta	179	tao
24	telo	76	vava	128	manome	180	mafana
25	efatse	77	hy	129	mifampitanjake	181	manintsy
26	lime	78	lela	130	mipiritsy	182	feno
27	bevata	79	hoho	131	mampikasoke	183	vaovao
28	lava	80	tomboke	132	manasa	184	antitse
29	malalake	81	ranjo	133	mamafa	185	soa
30	mateve	82	ongotse	134	mitifitse	186	raty
31	mavesatse	83	tana	135	manosike	187	mavay
32	malinike	84	elatse	136	manary	188	maloto
33	boribory	85	sarotso	137	mamehe	189	mahity
34	maifitse	86	tinay	138	mitsebike	190	boribory
35	matify	87	vozo	139	manisake	191	maranitse
36	ampela	88	lambosy	140	mivola	192	madomoke
37	lelahy	89	tsatsa	141	mihira	193	madoso
38	olo	90	fo	142	mihisa	194	le
39	anakaja	91	aty	143	mihafo	195	maike
40	valy	92	mino	144	mitsororoke	196	mete
41	valy	93	mihina	145	mandrike	197	marine
42	nene	94	manehitse	146	mivonto	198	lavitse
43	baba	95	mitsitsike	147	masoandro	199	ankavana
44	biby	96	mandrehoke	148	vola	200	ankavia
45	fia	97	mandoa	149	basia	201	ane
46	voro	98	mitsoke	150	rano	202	anaty
47	alika	99	miay	151	ora	203	amin ny
48	hao	100	mihehy	152	vavarano	204	sy
49	bibilava	101	mahita	153	kitoboke	205	laha
50	soko	102	mijanjy	154	riake	206	satsia
51	hazo	103	mahay	155	sira	207	anara
52	ala	104	mandinike	156	vato		

23 - Antaisaka (Vangaindrano)							
1	iaho	53	angira	105	magnorogny	157	fasiky
2	anao	54	vokazo	106	matatry	158	bo
3	izy	55	isikiky	107	mandry	159	tany
4	isika	56	raviny	108	velo	160	mika
5	indreo	57	vahitry	109	maty	161	zavo
6	anareo	58	oditrazo	110	mamono	162	lagnitry
7	itiky	59	vonikazo	111	miady	163	rivotry
8	zany	60	atry	112	mihaza	164	ranomandry
9	etoa	61	tady	113	mifiky	165	glatsy
10	ao	62	daro	114	manapaky	166	setroky
11	ia	63	hena	115	mamaky	167	afo
12	ino	64	ra	116	mitomboky antsy	168	lakevo
13	aia	65	tola	117	mifiky	169	magnoro
14	ovia	66	sabora	118	mihady	170	lala
15	akory	67	atody	119	milagno	171	vohitry
16	tsy	68	tandroky	120	mandrembo	172	mena
17	iaby	69	hoiny	121	mandeha	173	metso
18	maro	70	volomboro	122	avy	174	manamotamo
19	kidikidy	71	volo	123	mandry	175	fotsy
20	kidy	72	loha	124	mitoboky	176	mity
21	hafa	73	sofy	125	mitsanga	177	hariva
22	raiky	74	maso	126	mihody	178	andro
23	roy	75	oro	127	lavo	179	tao
24	telo	76	vava	128	magnome	180	mafana
25	efatry	77	nify	129	mitagna	181	manara
26	dimy	78	lela	130	mitery	182	feno
27	zakabe	79	vazakoho	131	magnakasoky	183	vao
28	abo	80	tomboky	132	manasa	184	atitry
29	malalaky	81	randro	133	mamafa	185	tsara
30	matevy	82	pokopoko	134	mitifitry	186	ratsy
31	mavesatry	83	tagna	135	manosiky	187	lo
32	kidy	84	elatry	136	mitoraky	188	maloto
33	fohy	85	troky	137	mamehy	189	mahitsy
34	pitsa	86	oliky	138	manjaitry	190	boribory
35	manify	87	vozo	139	magnisa	191	maragnitry
36	viavy	88	lamosy	140	mizaka	192	bofogna
37	lehilay	89	tratra	141	mihira	193	malama
38	olo	90	fo	142	mihira	194	le
39	zaza	91	aty	143	miepo	195	mainy
40	vady	92	mindrano	144	magnidina	196	marigny
41	vady	93	mihina	145	mandry	197	akeky
42	endry	94	magnifatry	146	mivonto	198	lavitry
43	aba	95	mitsetsitry	147	masoandro	199	akavana
44	biby	96	mandreoky	148	vola	200	akavia
45	vily	97	mandoa	149	vasia	201	agny
46	voro	98	misioky	150	rano	202	agnaty
47	amboa	99	miaigny	151	rikandro	203	ame
48	hao	100	mihehy	152	tegnarano	204	sy
49	bibilava	101	maita	153	dobondrano	205	ra
50	haka	102	miteno	154	ranomasy	206	satria
51	hazo	103	mahay	155	sira	207	agnara
52	ala	104	mandiniky	156	vato		

24 - Antankarana (Vohemar)							
1	zaho	53	kibay	105	maharegny	157	jia
2	anao	54	voankazo	106	mavozo	158	laposiera
3	izy	55	ambeo	107	mandry	159	tany
4	atsika	56	raviny	108	velogno	160	zavogno
5	anaro	57	vahatra	109	maty	161	rondro
6	iro	58	hoditry kakazo	110	mamono	162	lagnitry
7	ty	59	folera	111	miady	163	tsiko
8	zegny	60	ahitry	112	mihaza	164	lanezy
9	eto	61	tady	113	mamopoko	165	gilasy
10	ary	62	hoditry	114	manapaka	166	setroko
11	azovy	63	hena	115	mamaky	167	motro
12	ino	64	lio	116	manomboko	168	jofo
13	aia	65	taholagna	117	mikiky	169	magnoro
14	mbiagna	66	vondraka	118	mangady	170	lalagna
15	karakory	67	atody	119	milomagno	171	bongo
16	tsy	68	ampondo	120	mitiligny	172	mena
17	jiaby	69	ohiny	121	mandeha	173	maitso
18	maro	70	volomborona	122	tonga	174	manamotamo
19	tsy maro	71	fagneva	123	mandry	175	malandy
20	hely	72	loha	124	mipetraka	176	joby
21	hafa	73	sofigny	125	mitsangana	177	aligny
22	araiky	74	maso	126	magnodigny	178	andra
23	aroe	75	orogno	127	lavo	179	taogno
24	telo	76	vava	128	magnamia	180	mafana
25	efatra	77	nify	129	mitintigny	181	manintsy
26	dimy	78	lela	130	manery	182	feno
27	maventy	79	angofo	131	magnakasiky	183	vaovao
28	lava	80	vity	132	manasa	184	matoe
29	malalaka	81	kirandra	133	mamitry	185	tsara
30	matevigny	82	lohalitry	134	mitifitry	186	ratsy
31	mavesatra	83	tagnana	135	mandronjy	187	motraka
32	hely	84	elatra	136	manoraka	188	maloto
33	fohiky	85	kibo	137	mamehy	189	mahitsy
34	maletry	86	tsontsory	138	manjaitry	190	boribory
35	matify	87	vozogno	139	mikonty	191	maragnitry
36	magnangy	88	tahezagna	140	mivolagna	192	magofogno
37	lelahy	89	tratra	141	miantsa	193	malamatra
38	olo	90	fo	142	misoma	194	legny
39	tsaiky	91	haty	143	miefogno	195	maiky
40	vady	92	migiaka	144	mivalagna	196	marigny
41	vady	93	mihinagna	145	mandry	197	marikitry
42	nindry	94	magnekitry	146	mivona	198	lavitry
43	baba	95	minono	147	masoandro	199	ankavanana
44	biby	96	mihaka	148	fanjava	200	ankavia
45	laoko	97	mandoa	149	lakintagna	201	agny
46	vorogno	98	mitsoko	150	rano	202	agnatiny
47	amboa	99	miaigny	151	mahalegny	203	miaraka
48	hao	100	mitokiky	152	tegnandrano	204	ndraiky
49	bibilava	101	mizaha	153	farihy	205	raha
50	hankagna	102	mitandregny	154	ranomasigny	206	fotony
51	kakazo	103	mahay	155	sira	207	agnarana
52	atiala	104	mandiniky	156	vato		

25 - Betsileo (Ambositra)							
1	aho	53	tehina	105	mifofona	157	fasika
2	agnao	54	vokazo	106	matahotra	158	vovoka
3	ii	55	voa	107	matory	159	tany
4	sika	56	ravina	108	velogna	160	rahogna
5	agnareo	57	vahatra	109	maty	161	zavona
6	reo	58	hodikazo	110	mamono	162	lanitra
7	ty	59	voninkazo	111	miady	163	rivotra
8	mitroka	60	ahitra	112	mihaza	164	ranomandry
9	etohe	61	tady	113	mikapoka	165	ranomandry
10	ao	62	hoditra	114	mandidy	166	setroka
11	ia	63	hena	115	manilaky	167	afo
12	ina	64	ra	116	manatoka antsy	168	lavenona
13	aia	65	taolagna	117	mikiky	169	magnoro
14	viena	66	taviny	118	mangady	170	lalagna
15	akory	67	atody	119	mandagno	171	tendrobohitsa
16	tsa	68	tandroka	120	manidigna	172	mena
17	aby	69	rambo	121	mandeha	173	maitso
18	be	70	volomborogna	122	avy	174	vogny
19	kelikely	71	volo	123	mandrimandry	175	fotsy
20	kely	72	loha	124	mitoetra	176	maity
21	hafa	73	sofigna	125	mitsangagna	177	aligna
22	raika	74	maso	126	mihodigna	178	andro
23	roa	75	orogna	127	mienjika	179	taogna
24	telo	76	vava	128	manome	180	mafana
25	efatra	77	nify	129	mitantagna	181	mangitsy
26	dimy	78	lela	130	manery	182	feno
27	geda	79	hoho	131	manakasoka	183	vaovao
28	lava	80	tongotra	132	manasa	184	atitra
29	malalaka	81	ranjo	133	mamafa	185	soa
30	matevina	82	lohalika	134	mitifitra	186	ratsy
31	mavesatra	83	tagnana	135	manosika	187	lo
32	kely	84	elatra	136	mitoraka	188	maloto
33	fohy	85	kibo	137	manjaitra	189	mahitsy
34	tery	86	tsinay	138	manjaitsa	190	boribory
35	manify	87	vozogna	139	magnisa	191	maragnitra
36	vevavy	88	lamosigna	140	milaza	192	dombo
37	lelahy	89	tratra	141	mihira	193	malama
38	ona	90	fo	142	milalao	194	laigna
39	kilonga	91	aty	143	mitsikafogna	195	maigna
40	vady	92	migoka	144	mikoriagna	196	marina
41	vady	93	mihinana	145	mandry	197	mariny
42	neny	94	magnaikitra	146	mibotsigna	198	lavitra
43	baba	95	misika	147	masoandro	199	havanana
44	biby	96	mandrehoka	148	volana	200	havia
45	trondro	97	mandoa	149	kitana	201	agny
46	vorogna	98	mitsioka	150	rano	202	agnaty
47	amboa	99	miaigna	151	orana	203	amin ny
48	hao	100	mimehy	152	renirano	204	sy
49	bibilava	101	mahita	153	atara	205	reha
50	kakagna	102	miheno	154	ranomasina	206	satria
51	hazo	103	mahay	155	sira	207	agnarana
52	ala	104	mieritreritra	156	vato		

26 - Betsileo (Ambalavao)

1	iaho	53	tehy	105	mifofona	157	fasika
2	agnao	54	voakazo	106	matahotse	158	vovotany
3	i	55	voa	107	matory	159	tany
4	atsika	56	ravy	108	velo	160	raho
5	agnareo	57	faka	109	maty	161	zavo
6	reo	58	hodikazo	110	mamono	162	lagnitse
7	itito	59	vonikazo	111	miady	163	rivotse
8	zao	60	ahitsa	112	mihaza	164	ranomandry
9	ateto	61	tady	113	manjera	165	gilasy
10	atato	62	hoditse	114	mandidy	166	setroke
11	ia	63	hena	115	mamaky	167	afo
12	ino	64	ra	116	manomboka mesa	168	laveno
13	aia	65	taolagna	117	mikiky	169	magnoro
14	ovia	66	jabora	118	mihady	170	lala
15	nahoa	67	atody	119	milagno	171	tendromboitse
16	tsa	68	tsifa	120	mitsidy	172	mena
17	aby	69	rambo	121	mandeha	173	maitso
18	be	70	volomboro	122	avy	174	mavo
19	kelikely	71	volo	123	mandry	175	fotsy
20	kele	72	loha	124	mitoatse	176	mainty
21	hafa	73	sofy	125	mitsanga	177	aly
22	raika	74	maso	126	mihody	178	andro
23	roa	75	oro	127	migebo	179	tao
24	telo	76	vava	128	magnome	180	mafana
25	efatse	77	nify	129	mitagna	181	manara
26	dimy	78	lela	130	mamotsika	182	feno
27	lihibe	79	hoho	131	magnasoka	183	vaovao
28	lava	80	tongotse	132	manasa	184	atitse
29	malalake	81	kirango	133	mamafa	185	soa
30	matevy	82	lohalika	134	mitifotse	186	ratsy
31	mavesatse	83	tagna	135	manosike	187	lo
32	kele	84	elatsa	136	mitorake	188	maloto
33	fohy	85	troky	137	mamehe	189	mahitsy
34	tere	86	tsinay	138	manjaitse	190	boribory
35	manify	87	vozo	139	magnisa	191	maragnitsa
36	apela	88	lamosy	140	mitaro	192	dombo
37	lihilahy	89	tratra	141	mihira	193	malama
38	olo	90	fo	142	miregny	194	mando
39	kilonga	91	aty	143	mitsingafo	195	maigna
40	vady	92	misotro	144	mikoria	196	mety
41	vady	93	mihina	145	mandry	197	mariny
42	nene	94	magnehitsa	146	magnabotsigna	198	lavitse
43	baba	95	misike	147	masoandro	199	havana
44	biby	96	mandrehoka	148	volagna	200	havia
45	talapia	97	mandoa	149	kintana	201	agny
46	voro	98	mifioky	150	rano	202	agnaty
47	amboa	99	miaigna	151	ora	203	amin ny
48	hao	100	mihehy	152	vavarano	204	sy
49	bibilava	101	mahita	153	farihy	205	lehe
50	haka	102	mitaino	154	ranomasina	206	satria
51	hazo	103	mahay	155	sira	207	agnara
52	ala	104	mieritseritsa	156	vato		

27 - Antanalana (Itampolo)							
1	iraho	53	kobay	105	manimboke	157	faseke
2	iriha	54	voankazo	106	mavaka	158	lemboke
3	ireke	55	tabiry	107	miroro	159	tane
4	itika	56	ravene	108	velo	160	raho
5	iereo	57	fotone	109	mate	161	mika
6	iareo	58	holinketae	110	mamono	162	lanitse
7	itike	59	folera	111	mialy	163	tioke
8	izay	60	akata	112	mihaza	164	ranomandrike
9	etoa	61	taly	113	mamango	165	ranomandrike
10	ao	62	holitse	114	manampake	166	setroke
11	ia	63	hena	115	mizara	167	afo
12	ino	64	lio	116	mitomboke meso	168	lavenoke
13	aia	65	taola	117	mandraotse	169	manoro
14	ombia	66	saborane	118	mihaly	170	lala
15	akore	67	atoly	119	milano	171	vohitse
16	tsie	68	tsifa	120	mitily	172	mena
17	iaby	69	solone	121	mindeha	173	maitso
18	maro	70	volomboro	122	avy	174	vone
19	tsy ampe	71	volo	123	miroro	175	foty
20	kele	72	loha	124	mitoboke	176	mainty
21	hafa	73	sofy	125	mitsanga	177	haleke
22	raike	74	maso	126	miodike	178	andro
23	roe	75	oro	127	mitonta	179	tao
24	telo	76	vava	128	manome	180	mae
25	efatre	77	hy	129	mitanjake	181	manintsy
26	lime	78	famelake	130	mitere	182	feno
27	bey	79	hoho	131	mikasoke	183	vaovao
28	lava	80	tomboke	132	manasa	184	antitse
29	malalake	81	ranjo	133	mamafa	185	soa
30	mateve	82	ongotse	134	mitifitse	186	raty
31	tsiongake	83	tanake	135	manosike	187	taratavohe
32	malinike	84	elatse	136	atorake	188	maloto
33	boribory	85	troke	137	mandrohy	189	mahity
34	maifitse	86	tinay	138	mitsebeke	190	fohe
35	matify	87	vozo	139	manisake	191	maranitse
36	ampisafe	88	lambosy	140	mivola	192	madomoke
37	lahilahy	89	tratra	141	miantsa	193	madoso
38	ndaty	90	fo	142	mihisa	194	le
39	ajaja	91	aty	143	mihafo	195	maike
40	roakemba	92	mino	144	mijia	196	mete
41	roandria	93	mitava	145	mandreke	197	anila
42	nene	94	manehetse	146	mivonto	198	lavitse
43	baba	95	mitsintsike	147	masoandro	199	ankavana
44	biby	96	mandrehoke	148	vola	200	ankavia
45	fia	97	mandoa	149	kinta	201	aroke
46	voro	98	mitioke	150	rano	202	ampo
47	amboa	99	miay	151	ora	203	amine
48	hao	100	homehe	152	ranobey	204	noho
49	bibilava	101	mahita	153	dobo	205	laha
50	soko	102	mijanjy	154	riake	206	satria
51	hetae	103	mahay	155	sira	207	anara
52	ala	104	mandineke	156	vato		

28 - Vezo (Morombe)							
1	zaho	53	kobay	105	manimbo	157	fasy
2	iha	54	voankazo	106	matahotse	158	tsemboky
3	ie	55	voa	107	miroro	159	tokotany
4	tsika	56	ravy	108	velo	160	hiboke
5	nareo	57	fototse	109	mate	161	rahona
6	rozy	58	holinkazo	110	mamono	162	lanitse
7	toy	59	folera	111	mialy	163	tsioke
8	zay	60	akata	112	mihaza	164	ranomivonga
9	etoa	61	hosy	113	mamango	165	vongandrano
10	ao	62	holitse	114	manapake	166	setsoke
11	ia	63	hena	115	mamake	167	bolo
12	ino	64	lio	116	mitomboke meso	168	lavenoke
13	aia	65	tahola	117	mikihe	169	tsofora
14	ombia	66	vonjake	118	mihaly	170	lala
15	akory	67	atoly	119	milano	171	vohitse
16	tsy	68	tsifa	120	mitily	172	mena
17	iaby	69	hohy	121	mandehana	173	maintso
18	maro	70	volomboro	122	avy	174	mavo
19	tsiampeampe	71	volo	123	miroro	175	foty
20	tsiampe	72	loha	124	mitoboke	176	mainte
21	hafa	73	sofy	125	mitsanga	177	haly
22	raike	74	maso	126	miodike	178	anjo
23	roe	75	oro	127	latsaky	179	tao
24	telo	76	vava	128	manome	180	mafana
25	efatse	77	hy	129	mifampitanjake	181	manintsy
26	lime	78	lela	130	mitinndry	182	feno
27	bevata	79	nao	131	mampikasoke	183	vaovao
28	lava	80	tomboke	132	manasa	184	antitse
29	malalake	81	randro	133	mamafa	185	soa
30	mateve	82	hongotsy	134	mitifitse	186	raty
31	mavesatse	83	tana	135	manosike	187	mavay
32	kelikely	84	helatsy	136	manary	188	maloto
33	boriboriky	85	sarotso	137	mamehe	189	mahite
34	maifitse	86	tinay	138	mitsebike	190	boriboriky
35	matify	87	vozo	139	manisake	191	maranitse
36	ampela	88	lambosy	140	mivola	192	madomoke
37	johary	89	tsatsa	141	mihira	193	madoso
38	olo	90	fo	142	mihisa	194	loba
39	anakaja	91	aty	143	mihafo	195	maike
40	valy	92	mino	144	mitsororoke	196	mete
41	valy	93	mihina	145	manjike	197	marine
42	nene	94	manehitse	146	mivonto	198	lavitse
43	baba	95	mitsitsike	147	masoanjo	199	ankavana
44	biby	96	manjehoke	148	vola	200	ankavia
45	fia	97	mandoa	149	basia	201	ane
46	voro	98	mitsoke	150	rano	202	anaty
47	alika	99	miay	151	ora	203	ami y
48	hao	100	mihehy	152	vavarano	204	noho
49	bibilava	101	mahita	153	kitoboke	205	laha
50	soko	102	mijandry	154	riake	206	satsia
51	hazo	103	mahay	155	sira	207	anara
52	ala	104	mandinike	156	vato		

29 - Antanosy (Bezaha)							
1	zaho	53	begegy	105	magnitsy	157	fasy
2	iha	54	tengnany	106	matahosy	158	lemboky
3	izy	55	vihiny	107	mandry	159	tany
4	sika	56	raviny	108	velo	160	mandrahony
5	handreo	57	vahany	109	maty	161	zavony
6	lahireo	58	holikazo	110	namono	162	langnisy
7	itiho	59	folera	111	mialy	163	rivosy
8	zao	60	akata	112	mamita	164	ranomihandro
9	etoa	61	taly	113	manjera	165	ranomihandro
10	ao	62	holisy	114	manapaky	166	setroky
11	ija	63	hena	115	mamaky	167	afo
12	ino	64	lio	116	mitomboky mesa	168	lavenoky
13	eza	65	taola	117	magnihy	169	magnoro
14	ombia	66	vondrany	118	mihaly	170	lala
15	akory	67	atoly	119	milagno	171	vohisy
16	sizy	68	sifany	120	mitily	172	mena
17	sikaby	69	ohihy	121	mandeha	173	menso
18	kidikidy	70	volomboro	122	avy	174	mavo
19	kelikeliky	71	volo	123	mandry	175	fosy
20	siampy	72	loha	124	mitoboky	176	menty
21	hafahafa	73	sofy	125	misanga	177	hariva
22	raiky	74	maso	126	miolaky	178	andro
23	ro	75	oro	127	lasaky	179	tao
24	telo	76	vava	128	manolosy	180	mafana
25	efasy	77	nify	129	mita	181	manara
26	dimy	78	lela	130	mitery	182	feno
27	befoloay	79	vazakoho	131	mikasoky	183	vaovao
28	lava	80	tomboky	132	manasa	184	atisy
29	malalaky	81	randro	133	mamafa	185	soa
30	matevine	82	pokopoko	134	mitifisy	186	rasy
31	mavesasy	83	tagna	135	manosiky	187	lo
32	masitiky	84	elany	136	mitoraky	188	maloto
33	boribory	85	troky	137	mamehy	189	mahisy
34	tery	86	sinainy	138	mazaisy	190	boribory
35	matify	87	vozo	139	magnisaky	191	marangnisy
36	apela	88	lambosy	140	mizaka	192	dofoky
37	rangahy	89	harangna	141	mibeko	193	malama
38	olo	90	fo	142	mihisa	194	lende
39	zaza	91	atiny	143	mihafo	195	maiky
40	valy	92	mino	144	mivary	196	mety
41	valy	93	mihina	145	miandrony	197	mariniky
42	reny	94	manifasy	146	mibognaky	198	lavisy
43	baba	95	fiazo	147	masoandro	199	akavana
44	biby	96	mandrehoky	148	volana	200	akavia
45	fia	97	mandoa	149	vasia	201	agny
46	voro	98	misioky	150	rano	202	agnatiny
47	amboa	99	miay	151	ora	203	amin ny
48	hao	100	homehy	152	renirano	204	sy
49	bibilava	101	mahita	153	farihy	205	raha
50	soko	102	miteno	154	riaky	206	satria
51	hazo	103	mahay	155	sira	207	agnara
52	ala	104	mieriserisy	156	vato		

30 - Tanala (Ifanadiana)							
1	iaho	53	salohy	105	magnerana	157	fasika
2	enao	54	isinjavatra	106	vaka	158	vovoka
3	izy	55	isy	107	matory	159	tany
4	atsena	56	ravy	108	velo	160	mika
5	ianareo	57	faka	109	maty	161	zavo
6	izy ireo	58	hodikazo	110	mamono	162	lanitry
7	ity	59	voninkazo	111	miady	163	rivotra
8	izany	60	ahitra	112	miremby	164	ranomivengany
9	eto	61	tady	113	mikapoka	165	ranomivengany
10	atsy	62	hoditra	114	mandrasa	166	setroky
11	iza	63	hena	115	mamaky	167	afo
12	ino	64	ra	116	mifira antsy	168	lakevo
13	aiza	65	taolana	117	mikisiky	169	mandoro
14	vina	66	taviny	118	mangady	170	lalana
15	ahoana	67	atody	119	lomano	171	tendrombohitra
16	tsy	68	tandroka	120	magnembony	172	mena
17	aby	69	ohony	121	mandeha	173	metso
18	maro	70	volomborona	122	tonga	174	mavo
19	vitsy	71	volo	123	mandry	175	fotsy
20	kely	72	loha	124	mipetraka	176	minty
21	hafa	73	tadiny	125	mitsangana	177	aliny
22	iray	74	maso	126	mihodina	178	andro
23	roa	75	orona	127	latsaky	179	tao
24	telo	76	vava	128	matariky	180	mevoky
25	efatra	77	nify	129	mitana	181	mangatsiaka
26	dimy	78	lela	130	mipitsa	182	feno
27	lehibe	79	vazakoho	131	manakasoka	183	vao
28	lava	80	tongotra	132	manasa	184	antitry
29	malalaka	81	randro	133	mamafa	185	tsara
30	matevina	82	lohalika	134	mitifitra	186	ratsy
31	mavesatra	83	tanana	135	manosika	187	lo
32	kidiky	84	elatra	136	mitoraky	188	maloto
33	fohy	85	kibo	137	mamehy	189	mahitsy
34	tery	86	oliky	138	manjaitra	190	boribory
35	manify	87	vozona	139	manisa	191	maranitra
36	viavy	88	lamosina	140	mizaka	192	bofoky
37	lehilahy	89	tratra	141	mihira	193	malama
38	olona	90	fo	142	mihira	194	lena
39	zaza	91	aty	143	mitsingevaheva	195	maina
40	vady	92	misotro	144	mikoriana	196	mety
41	vady	93	mihomana	145	mandry	197	marivo
42	reny	94	magnekitry	146	voky	198	lavitra
43	ray	95	manery	147	masoandro	199	havanana
44	biby	96	mandrehoka	148	volany	200	avia
45	laoka	97	mandoa	149	kintana	201	any
46	vorona	98	mitsoka	150	rano	202	anaty
47	alika	99	miaina	151	orony	203	amin ny
48	hao	100	mihomehy	152	renirano	204	de
49	bibilava	101	mahita	153	dobo	205	raha
50	kankana	102	miteno	154	riaky	206	satria
51	hazo	103	mahay	155	sira	207	anara
52	ala	104	mieritreritra	156	vato		

31 - Bara (Ranohira)

1	iaho	53	kobay	105	magnembo	157	fasy
2	anao	54	voakazo	106	matahotsy	158	bo
3	i	55	vihy	107	matory	159	tany
4	tsika	56	ravy	108	velo	160	raho
5	anareo	57	vahatsy	109	maty	161	zavo
6	reo	58	holintsazo	110	mamono	162	lagnitsy
7	toy	59	vonikazo	111	mialy	163	tsioky
8	zay	60	akata	112	miremby	164	zavo
9	eto	61	taly	113	mamofoky	165	ranomandry
10	ao	62	holitsy	114	manapaky	166	setroky
11	ia	63	hena	115	manilaky	167	afo
12	ino	64	ra	116	mitomboky mesa	168	lavenoky
13	aia	65	tola	117	hihisa	169	magnodo
14	ombia	66	sabora	118	mihaly	170	lala
15	akory	67	atody	119	milagno	171	vohitsy
16	tsy	68	tsifa	120	mitily	172	mena
17	aby	69	ohiny	121	mandeha	173	metso
18	lako	70	volomboro	122	avy	174	vogny
19	kidikidy	71	volo	123	mandry	175	fotsy
20	kidy	72	loha	124	mitoboky	176	mety
21	hafa	73	sofy	125	mitsanga	177	hariva
22	raiky	74	maso	126	mitodiky	178	andro
23	roy	75	oro	127	lavo	179	tao
24	telo	76	vava	128	manolotsy	180	mafana
25	efatsy	77	hy	129	mita	181	manitsy
26	dimy	78	lela	130	mamiaky	182	feno
27	foloay	79	vazakoho	131	mampikasoky	183	vao
28	abo	80	tomboky	132	manasa	184	antitsy
29	malalaky	81	randro	133	mamafa	185	soa
30	matevy	82	pokopoko	134	mitifitsy	186	ratsy
31	mavesatsy	83	tagna	135	mandrosy	187	lo
32	kely	84	elatsy	136	manoraky	188	motaky
33	pohy	85	troky	137	mamehy	189	mahitsy
34	detsy	86	tsinay	138	mitrebiky	190	boribory
35	matify	87	vozo	139	magnisa	191	maragnitsy
36	apela	88	lambosy	140	mitsara	192	do
37	lelahy	89	tratra	141	miantsa	193	malama
38	olo	90	fo	142	mihisa	194	mando
39	zaza	91	aty	143	mihefo	195	maiky
40	vady	92	mino	144	mandeha	196	mety
41	vady	93	mihina	145	mandry	197	marikitsy
42	neny	94	magnehitsy	146	mivonto	198	lavitsy
43	aba	95	mitsetsiky	147	tanonandroky	199	havana
44	biby	96	mandora	148	vola	200	havia
45	fia	97	mandoa	149	vasia	201	agny
46	voro	98	mitsioky	150	rano	202	agnatiny
47	amboa	99	miay	151	ora	203	mindray
48	hao	100	mihehy	152	vavarano	204	la
49	bibilava	101	mahita	153	ranovory	205	laha
50	haka	102	maheno	154	riaky	206	satria
51	hazo	103	mahay	155	sira	207	agnara
52	ala	104	mieritseritsy	156	vato		

32 - Antanalana (Manorofify)							
1	iaho	53	kobay	105	manimbo	157	fase
2	riha	54	voankazo	106	matahotse	158	lemboke
3	reke	55	vihy	107	miroro	159	tane
4	tika	56	rave	108	velo	160	raho
5	nareo	57	vahatse	109	mate	161	manjavo
6	iereo	58	holitsetae	110	mamono	162	lanitse
7	toy	59	ravenkazo	111	mialy	163	tioke
8	zane	60	ahitse	112	mihaza	164	ranomandreke
9	etoa	61	taly	113	mamango	165	ranomandreke
10	ao	62	holitse	114	manampake	166	setroke
11	ia	63	hena	115	mamaky	167	afo
12	ino	64	lio	116	mitomboke meso	168	lavenoke
13	aia	65	taola	117	mandraotse	169	manoro
14	ombia	66	vondrake	118	mihaly	170	lala
15	akore	67	atoly	119	milano	171	vohitse
16	tsy	68	tsifa	120	mitily	172	mena
17	iaby	69	ohy	121	mandeha	173	maintso
18	maro	70	volomboro	122	avy	174	vone
19	vitsivitsy	71	volo	123	mandre	175	foty
20	kele	72	loha	124	mipetrake	176	mainte
21	hafa	73	sofy	125	mitsanga	177	hale
22	raike	74	maso	126	mitolike	178	andro
23	roe	75	oro	127	mitonta	179	tao
24	telo	76	vava	128	manomey	180	mae
25	efatse	77	hy	129	mitanjake	181	manintsy
26	lime	78	lela	130	mitere	182	atseke
27	bevata	79	hoho	131	mikoseke	183	vaovao
28	lava	80	tomboke	132	manasa	184	antetse
29	malalake	81	bositomboke	133	mamafa	185	soa
30	mateve	82	ongotse	134	mitifitse	186	raty
31	mavesatse	83	tana	135	manoseke	187	lo
32	kele	84	elatse	136	mitorake	188	maloto
33	boribory	85	troke	137	mandrohy	189	mahity
34	maifitse	86	tinay	138	mitrebeke	190	fohe
35	matify	87	vozo	139	manisake	191	maranitse
36	ampela	88	lambosy	140	mivola	192	madomoke
37	lahilahy	89	tratra	141	miantsa	193	madoso
38	ndaty	90	fo	142	misa	194	mando
39	aja	91	ate	143	mihafo	195	maike
40	valy	92	mino	144	milenteke	196	mare
41	valy	93	mihina	145	mandreke	197	marine
42	rene	94	manehetse	146	mivonto	198	lavitse
43	baba	95	misintsike	147	masoandro	199	havana
44	biby	96	mandrehoke	148	volane	200	ankavia
45	fia	97	mandoa	149	vasia	201	ane
46	voro	98	mitsoke	150	rano	202	ampone
47	amboa	99	miay	151	ora	203	amy
48	hao	100	mihehe	152	vavarano	204	naho
49	bibilava	101	mahisake	153	kotoboke	205	laha
50	soko	102	mijanjy	154	riake	206	fa
51	hetae	103	mahay	155	sira	207	anara
52	ala	104	mandineke	156	vato		

33 - Antandroy (Toliara)							
1	iraho	53	kobay	105	mamofontse	157	fasegne
2	irehe	54	voa	106	matahotse	158	lemboke
3	ihe	55	voa e	107	miroro	159	tane
4	itikagne	56	rave e	108	velogne	160	rahogne
5	inareo	57	vaha e	109	vilasy	161	zavogne
6	inareo io	58	holinkazo	110	mamono	162	lagnitse
7	intoy	59	felankazo	111	mialy	163	rivotse
8	izao	60	boka	112	magnaranto	164	ranomivongagne
9	eto	61	taly	113	mijera	165	ranomivongagne
10	ao	62	sandry	114	manampake	166	setroke
11	ia	63	hena	115	mamaky	167	afo
12	inogne	64	lio	116	mamiry	168	lavenoke
13	aiao	65	taolagne	117	magnofe	169	magnoro
14	ombiagne	66	saborae	118	mihaly	170	lalagne
15	akore	67	atoly	119	milaogne	171	vohitse
16	tsie	68	tsifa	120	mitiligne	172	mena
17	iaby	69	hoie	121	mandeha	173	maintso
18	tsiefa	70	volomborogne	122	avy	174	mavo
19	kidikidie	71	volo	123	mirotsy	175	foty
20	tsiampe	72	agnamboe	124	mitoboke	176	mainte
21	hafa	73	ravindia	125	mitsangagne	177	halegne
22	raike	74	fivajohoa	126	mihodigne	178	andro
23	roe	75	orogne	127	mitonta	179	taogne
24	telo	76	falie	128	magnomey	180	mafana
25	efatse	77	nife	129	minday	181	manintsy
26	lime	78	famelike	130	misere	182	feno
27	bey	79	hoho	131	mampikasike	183	vaovao
28	lava	80	tomboke	132	manasa	184	antetse
29	malalake	81	taolamaigne	133	mamafa	185	soa
30	matevegne	82	ongotse	134	mitifitse	186	raty
31	mavesatse	83	tagnagne	135	manoseke	187	lo
32	kede	84	elatse	136	mitorake	188	maloto
33	bory	85	tsoke	137	mamehe	189	mahity
34	tsiombe	86	tsinay	138	manjaitse	190	borybory
35	tsingarakarake	87	vozogne	139	magnisake	191	maragnitse
36	ampela	88	lambosigne	140	mitalily	192	bey
37	lahilahy	89	tratra	141	miantsa	193	malama
38	ndaty	90	arofo	142	mihisa	194	legne
39	ajaja	91	ate	143	mihafogne	195	maike
40	valy	92	minogne	144	mandeha	196	mete
41	valy	93	mihinagne	145	mandre	197	marine
42	inene	94	mitifatse	146	mibokinake	198	lavitse
43	baba	95	mihorogne	147	masoandro	199	havagne
44	biby	96	mikohake	148	volagne	200	havia
45	fiagne	97	mandoa	149	kintagne	201	agne
46	vorogne	98	mipoliotse	150	rano	202	agnate e
47	amboa	99	miaigne	151	oragne	203	ama e
48	hao	100	mihehe	152	loharano	204	sy
49	meregne	101	mahatsea	153	saka	205	raha
50	kakagne	102	mijajigne	154	ranomasigne	206	naho
51	hatagne	103	mahafatatse	155	sira	207	agnaragne
52	ala	104	mieritseretse	156	vato		

34 - Antanalana (Anakao)

1	iaho	53	kobay	105	manimbo	157	fase
2	riha	54	voanketa	106	matahotse	158	lemboke
3	reke	55	voa	107	miroro	159	tane
4	tsika	56	rave	108	velo	160	raho
5	nareo	57	fototsy	109	mate	161	zavo
6	iereo	58	holinkazo	110	mamono	162	lanitsy
7	intoke	59	folera	111	mialy	163	tsioke
8	zay	60	ahetsy	112	mihaza	164	riko
9	etoa	61	taly	113	mamango	165	gilasy
10	ao	62	holitsy	114	mandily	166	setroke
11	ia	63	hena	115	mizara	167	afo
12	ino	64	lio	116	mitomboke meso	168	lavenoke
13	aia	65	taola	117	mikihy	169	manoro
14	ombia	66	vondrany	118	mihaly	170	lala
15	akore	67	atoly	119	milano	171	vohitsy
16	tsy	68	tsifa	120	mitily	172	mena
17	iaby	69	ohy	121	mandeha	173	maintso
18	maro	70	volomboro	122	avy	174	mavo
19	kelikely	71	volo	123	mandre	175	foty
20	kele	72	loha	124	mitoboke	176	mainty
21	hafa	73	sofy	125	mitsanga	177	hale
22	raiky	74	maso	126	miodiky	178	andro
23	roe	75	oro	127	mitonta	179	tao
24	telo	76	vava	128	manome	180	may
25	efatse	77	hy	129	mitanjake	181	manintsy
26	lime	78	lela	130	mipiritsy	182	atsike
27	bevata	79	hoho	131	mikoseke	183	vaovao
28	lava	80	tomboke	132	manasa	184	antitsy
29	malalake	81	fiotsy	133	mamafa	185	soa
30	mateve	82	lohalike	134	mitifitsy	186	raty
31	mavesatse	83	tanake	135	manosiky	187	simba
32	kele	84	elatsy	136	manoraky	188	maloto
33	boribory	85	troke	137	mandrohy	189	mahity
34	maifitsy	86	tinay	138	mitrebeke	190	boribory
35	matify	87	vozo	139	manisake	191	maranetse
36	ampisafe	88	lambosy	140	mivola	192	domoke
37	jiahy	89	tratra	141	miantsa	193	midisa
38	ndaty	90	fo	142	mihisa	194	le
39	aja	91	aty	143	mihafo	195	maiky
40	valy	92	mino	144	migororosy	196	mete
41	valy	93	homa	145	mimpandriky	197	marine
42	nene	94	manehetse	146	mivonto	198	lavitsy
43	baba	95	minono	147	masoandro	199	longo
44	biby	96	mandrehoke	148	vola	200	havia
45	fia	97	mandoa	149	vasia	201	any
46	voro	98	mitsoke	150	rano	202	anaty
47	amboa	99	miay	151	ora	203	amine
48	hao	100	homehe	152	renerano	204	noho
49	bibilava	101	mahita	153	kotoboky	205	laha
50	tsoko	102	mijanjy	154	riake	206	satria
51	keta	103	mahaony	155	sira	207	anara
52	ala	104	mieretseretse	156	vato		

35 - Betsimisaraka (Marolambo)

1	iao	53	fioka	105	miorogna	157	fasika
2	ano	54	vonkazo	106	matahotra	158	vovoka
3	ie	55	vihy	107	mandry	159	tane
4	ansena	56	ravina	108	velona	160	raona
5	anaro	57	vahatra	109	mate	161	zavona
6	zaro	58	hoditra kakazo	110	mamono	162	lanitra
7	ty	59	voninkazo	111	mamango	163	rivotra
8	iane	60	ahitra	112	mitadia	164	ranomandry
9	aketo	61	tade	113	mamango	165	ranomandry
10	ako	62	oditra	114	manapaka	166	toesina
11	ive	63	ena	115	mizara	167	afo
12	ino	64	elatra	116	manombok anse	168	lavenina
13	akaia	65	tolagna	117	mikiky	169	magnoro
14	oviana	66	menagny	118	mangade	170	lalana
15	aona	67	atode	119	mandagno	171	tendrombohitra
16	sy	68	tandroka	120	mirigna	172	mena
17	ziaby	69	volombody	121	mandeha	173	meso
18	maro	70	volomborogna	122	tonga	174	mavo
19	visivise	71	randrana	123	mandry	175	fose
20	bitaka	72	loha	124	mipetraka	176	mintina
21	afa	73	tadigne	125	misangana	177	alina
22	reka	74	maso	126	miodigna	178	andro
23	roe	75	orogna	127	lavo	179	taona
24	telo	76	vava	128	magnome	180	mafana
25	efara	77	nife	129	mitantana	181	manara
26	dime	78	lela	130	mampipositra	182	feno
27	lebe	79	angofo	131	mampikasoka	183	vovo
28	lava	80	ongotra	132	manasa	184	antitra
29	matahitra	81	randro	133	mamafa	185	sara
30	matevina	82	lohalitra	134	mitifitra	186	rase
31	mavesara	83	tagnana	135	manosika	187	lo
32	bitaka	84	elara	136	mambalavala	188	maloto
33	fohika	85	kibo	137	mampitohy	189	mahise
34	tere	86	olika	138	manzetra	190	tabolabola
35	manife	87	vozogna	139	magnisa	191	marangitra
36	viave	88	tambokoka	140	mizaka	192	malombona
37	lilae	89	tratra	141	mihira	193	malady
38	olo	90	fo	142	milalo	194	mando
39	iaia	91	ate	143	miboagna	195	megna
40	vade	92	midroka	144	midoroka	196	mety
41	vade	93	mihinana	145	mampivaingana	197	marivo
42	mama	94	magnekitra	146	mibonsigna	198	lavitra
43	iaba	95	misestra	147	masoandro	199	havanana
44	bibe	96	mandrehoka	148	volana	200	havia
45	loka	97	mandoa	149	kintana	201	agny
46	vorogna	98	misotra	150	rano	202	agnaty
47	kiva	99	malak egna	151	orana	203	miaraka aminny
48	o	100	mimehy	152	tegnarano	204	sy
49	bibilava	101	mizaha	153	farihy	205	raha
50	ankagna	102	miteno	154	ranomasina	206	satria
51	kakazo	103	mahe	155	sira	207	agnarana
52	atiala	104	mieritreritra	156	vato		

36 - Betsimisaraka (Antsiranana)							
1	za	53	kobay	105	mifofogno	157	jia
2	anao	54	voankazo	106	mavozo	158	vovoko
3	izy	55	voany	107	mandry	159	tany
4	atsika	56	ravina	108	velogno	160	rondro
5	anaro	57	faka	109	maty	161	zavogno
6	ro	58	hodikazo	110	mamono	162	lanitra
7	ity	59	voninkazo	111	miady	163	tsiko
8	zegny	60	ahitra	112	mizaha	164	ranomanintsy
9	eto	61	tady	113	mamopoko	165	ranomanintsy
10	ao	62	hoditra	114	manapaka	166	setroko
11	azovy	63	hena	115	mamaky	167	motro
12	ino	64	lio	116	mitomboko meso	168	lavenogno
13	ahia	65	taolagna	117	mikiky	169	may
14	ombiagna	66	taviny	118	mangaty	170	lalagna
15	akory	67	atody	119	milamano	171	tendrombohitra
16	tsy	68	tandroko	120	mitiligny	172	mena
17	jiaby	69	vorimbody	121	mandeha	173	maitso
18	maro	70	volomborona	122	avy	174	mavo
19	vitsivitsy	71	fagneva	123	mandry	175	fotsy
20	hely	72	loha	124	mipetraka	176	joby
21	hafa	73	sofigny	125	mitsangana	177	aligny
22	araiky	74	maso	126	mihodigny	178	andra
23	aroe	75	orogno	127	lavo	179	taogno
24	telo	76	vava	128	magnamia	180	mafana
25	efatra	77	nify	129	mitana	181	manintsy
26	dimy	78	lela	130	manery	182	feno
27	be	79	angofy	131	manakasoka	183	vaovao
28	lava	80	vity	132	manasa	184	matoe
29	malalaka	81	fehy	133	mamafa	185	tsara
30	matevigny	82	lohalika	134	mitiritiry	186	ratsy
31	mavesatra	83	tagnana	135	magnaposy	187	lo
32	hely	84	elatra	136	mitoraka	188	maloto
33	foky	85	kibo	137	mamery	189	mahitsy
34	tery	86	tsontsory	138	manjaitry	190	boribory
35	matify	87	vozogno	139	miconty	191	marangitry
36	viavy	88	taezagna	140	mivolagna	192	magofogno
37	lela	89	tratra	141	mihira	193	malamatra
38	olo	90	fo	142	misaoma	194	matsitsiky
39	tsaiky	91	aty	143	mihempo	195	maiky
40	vady	92	migiaka	144	milentiky	196	mety
41	vady	93	mhina	145	mandry	197	marigny
42	reny	94	magnekitry	146	mibotaka	198	lavitra
43	ray	95	minono	147	masoandro	199	havananana
44	biby	96	mandoa	148	volagna	200	avia
45	loko	97	mandrora	149	lakitagna	201	havia
46	vorogno	98	mifiko	150	rano	202	agnaty
47	amboa	99	miaigny	151	malegny	203	amin ny
48	fagnofaka	100	mitokiky	152	renirano	204	sy
49	bibilava	101	mahita	153	mantsabory	205	raha
50	ankana	102	mitandregny	154	ranomasigny	206	satria
51	kakazo	103	mahay	155	sira	207	agnarana
52	atihala	104	miertitreritry	156	vato		

37 - Betsimisaraka (Brickaville)

1	zao	53	kakazo	105	marigny	157	fasika
2	ano	54	vonkazo	106	vaka	158	vovoka
3	izy	55	vihiny	107	mandry	159	rtany
4	antsena	56	ravina	108	vile	160	raona
5	andrio	57	fekany	109	maty	161	zavogna
6	zario	58	fotiny	110	mamono	162	lanitra
7	ity	59	flera	111	miady	163	rivotra
8	zany	60	ahitra	112	miaza	164	ranomivengana
9	akito	61	tady	113	mampoka	165	ranomivengana
10	ako	62	hoditra	114	manapaka	166	sitroka
11	izovy	63	ena	115	mizara	167	afo
12	ino	64	ra	116	manombok antsy	168	levogna
13	akeza	65	tolagna	117	mangaroaro	169	mandoro
14	oviana	66	taviny	118	mangady	170	lalana
15	akory	67	atody	119	mandagno	171	tamboo
16	tsia	68	tandroka	120	magnembana	172	mena
17	jiaby	69	rambo	121	mandeha	173	mitso
18	maro	70	volom borogna	122	avy	174	mavo
19	vitsy	71	randrana	123	mandry	175	fotsy
20	bitika	72	loa	124	mipetraka	176	mintina
21	hafa	73	tadigny	125	mitsangana	177	ale
22	reka	74	moso	126	miodigna	178	mandrena
23	roy	75	orogno	127	lavo	179	tona
24	telo	76	vava	128	magnamia	180	mamovoka
25	efatra	77	nify	129	mitana	181	mangatsiaka
26	dimy	78	lela	130	mitiry	182	feno
27	mavinty	79	angofo	131	magnakasoka	183	vovo
28	lava	80	tongotra	132	manasa	184	antitra
29	malalaka	81	vovitsy	133	firina	185	tsara
30	mativina	82	loalika	134	mitifitra	186	ratsy
31	mavesatra	83	tagnana	135	manosika	187	lo
32	bitika	84	elatra	136	manoratra	188	maloto
33	fohika	85	kibo	137	mamihy	189	mahitsy
34	tiry	86	tsine	138	manjetra	190	boribory
35	manify	87	tenda	139	magnisa	191	marangitra
36	viavy	88	lamosigny	140	mizaka	192	dombo
37	lilay	89	tratra	141	mihira	193	malama
38	ole	90	fo	142	midola	194	lina
39	zaza	91	aty	143	mihimpo	195	megna
40	vady	92	midroka	144	midroka	196	mety
41	vady	93	minana	145	mivengana	197	marivo
42	riny	94	mangikitra	146	mibotsogna	198	lavitra
43	ray	95	mitsitsitra	147	masoandro	199	avanana
44	biky	96	mandrioka	148	volana	200	avia
45	loka	97	mandoa	149	kintana	201	agny
46	vorogna	98	misotra	150	rano	202	agnaty
47	kiva	99	miegna	151	oragna	203	miaraka
48	ho	100	mivagnitika	152	tsiragnana	204	de
49	bibilava	101	maita	153	fariy	205	ko
50	ankagna	102	mitino	154	ranomasina	206	satria
51	kakazo	103	mae	155	sira	207	agnarana
52	ala	104	mieritreritra	156	vato		

38 - Betsimisaraka (Toamasina)

1	zaho	53	fioka	105	mifofogna	157	fasika
2	ano	54	vihy	106	matahotra	158	vovoka
3	izy	55	ravigna	107	mandry	159	tany
4	antsika	56	fototra	108	velogna	160	rahogna
5	anareo	57	hoditry	109	maty	161	zavogna
6	zare	58	folera	110	mamono	162	lanitra
7	ity	59	ahitra	111	miady	163	rivotra
8	njany	60	ahitra	112	mihaza	164	ranomandry
9	aketo	61	tady	113	mamango	165	ranomandry
10	indrogna	62	hoditra	114	manapaka	166	mifoka
11	zovy	63	hena	115	mirasa	167	afo
12	ino	64	ra	116	magnatsatoka antsy	168	lavenogno
13	akeza	65	taolagna	117	magnisika	169	mandoro
14	oviagna	66	tavy	118	mangady	170	lalana
15	ahoana	67	atody	119	mandagno	171	tendrombohitra
16	tsy	68	tandroka	120	magnembagna	172	mena
17	jiaby	69	volombody	121	mandeha	173	mentso
18	maro	70	volomborogna	122	avy	174	mavo
19	bitibitika	71	randragna	123	mandry	175	fotsy
20	bitika	72	loha	124	mantotry	176	mintigna
21	hafa	73	tadigny	125	mitsangana	177	aligna
22	areka	74	maso	126	mihodigna	178	andro
23	aro	75	orogna	127	lavo	179	taogno
24	telo	76	vava	128	magnome	180	mafana
25	efatra	77	nify	129	mitatagna	181	mangatsiaka
26	dimy	78	lela	130	manery	182	feno
27	ngeza	79	angofo	131	magnakasoka	183	vovo
28	lava	80	tongotra	132	manasa	184	antitry
29	matahitra	81	randro	133	mamafa	185	tsara
30	matevigna	82	lohalitra	134	mitifitra	186	ratsy
31	mavesatra	83	tagnana	135	manosika	187	lo
32	bitika	84	elatra	136	manipy	188	maloto
33	fohika	85	kibo	137	manohy	189	mahitsy
34	hety	86	tsine	138	manjetra	190	boribory
35	manify	87	vozogna	139	magnisa	191	marangitra
36	viavy	88	tambokoka	140	mivolagna	192	dombo
37	lilahy	89	tratra	141	mihira	193	malama
38	ologna	90	fo	142	midola	194	mando
39	zaza	91	aty	143	mitsingevagna	195	megna
40	vady	92	misotro	144	mikoriagna	196	marigna
41	vady	93	mihinagna	145	mivengagna	197	marivo
42	mama	94	magnekitra	146	mibotsigna	198	lavitra
43	papa	95	mitetsitra	147	masoandro	199	havanagna
44	biby	96	mandrehoka	148	volagna	200	havia
45	laoko	97	mandoa	149	kintagna	201	akagny
46	vorogno	98	mitsotra	150	rano	202	agnaty
47	kiva	99	miaigna	151	oragnandro	203	amin ny
48	hao	100	mimoehy	152	tegnarano	204	sy
49	bibilava	101	mahita	153	farihy	205	raha
50	ankagna	102	mitandregny	154	ranomasigna	206	satria
51	kakazo	103	mahafantatra	155	sira	207	agnaragna
52	atiala	104	mieritreritra	156	vato		

39 - Betsimisaraka (Mananara)							
1	zaho	53	koboay	105	mifofogno	157	halagnana
2	ana	54	voankazo	106	matahotra	158	vovoko
3	izy	55	vihiny	107	mandry	159	tany
4	atsika	56	ravigny	108	velogno	160	rondro
5	are	57	vahiny	109	maty	161	zavogno
6	zare	58	hoditry kakazo	110	mamono	162	lanitry
7	ito	59	folera	111	miady	163	rivotro
8	zegny	60	ahitry	112	mizora	164	glasy
9	aketo	61	tady	113	mamopoko	165	glasy
10	aka	62	hoditry	114	manapaka	166	emboko
11	zovy	63	hena	115	mamaky	167	afo
12	ino	64	ra	116	monombo kiso	168	jofo
13	aiza	65	taholagna	117	mikiky	169	magnoro
14	aforiagna	66	taviny	118	mangady	170	lalagna
15	karakory	67	antody	119	milomagno	171	tanety
16	ehe	68	tandroko	120	manidigny	172	mena
17	jiaby	69	vilimbondiny	121	mandeha	173	maitso
18	fontry	70	volovolony	122	avy	174	hasaka
19	hely	71	vorondoha	123	mandry	175	fotsy
20	hely	72	talandoha	124	mantotry	176	maintigny
21	hafa	73	tadigny	125	mitsangana	177	aligny
22	araiky	74	maso	126	mihodigny	178	andro
23	aro	75	orogno	127	lavo	179	taogno
24	telo	76	vava	128	mangame	180	mafana
25	efatra	77	nify	129	mitantagna	181	mangatsiaka
26	dimy	78	lela	130	manery	182	feno
27	maventy	79	angofo	131	magnakasiky	183	vao
28	lava	80	hongotro	132	manasa	184	antitry
29	malalaka	81	vavitsy	133	mamafa	185	tsara
30	matomboko	82	lohalitry	134	mitifitry	186	ratsy
31	mavesatra	83	tanagna	135	manosiky	187	iho
32	madiniky	84	helatra	136	manoraka	188	maloto
33	fohy	85	boko	137	mamehy	189	mahitsy
34	maety	86	tsinay	138	manjaitry	190	boribory
35	matify	87	ambozogno	139	magnisa	191	maragnitry
36	viavy	88	tahezagna	140	mivolagna	192	lentigny
37	lalahy	89	tratra	141	mihira	193	malamatra
38	olo	90	fo	142	midaola	194	mandoa
39	gamadiniky	91	aty	143	miempo	195	maigny
40	vady	92	homagna	144	mikoriagna	196	manjary
41	vady	93	homagna	145	mandry	197	mariny
42	mama	94	magnekitry	146	mivonto	198	lavitry
43	baba	95	milelatra	147	masova	199	akavanana
44	kaka	96	mandrehoko	148	davolagna	200	ankavia
45	loko	97	mandoa	149	lakitagna	201	angy
46	vorogno	98	milefa	150	rano	202	angatiny
47	kiva	99	miaigny	151	oragnandro	203	aminy
48	hao	100	mimoehy	152	tegna rano	204	sy
49	biby	101	mahita	153	farihy	205	koa
50	hankagna	102	mitandregny	154	ranomasigny	206	satria
51	kakazo	103	mahay	155	sira	207	agnaragna
52	atiala	104	mieritreritra	156	vato		

40 - Tsimihety (Mampikony)							
1	zaho	53	kobay	105	maharegny	157	alagnagna
2	anao	54	voankazo	106	matahotro	158	jofo
3	izy	55	ambeo	107	mandry	159	tany
4	atsika	56	ravigny	108	velogno	160	rondro
5	areo	57	vahany	109	maty	161	zavogno
6	zareo	58	hodikakazo	110	mamono	162	lagnitry
7	toy	59	felagna	111	miady	163	rivotro
8	zany	60	ahitry	112	mijoko	164	lanezy
9	aketo	61	tady	113	mamiko	165	gilasy
10	aroy	62	hoditry	114	manapaka	166	tsemboko
11	azovy	63	hena	115	mamaky	167	motro
12	ino	64	lio	116	manomboko	168	jofo
13	aiza	65	taholagna	117	mikiky	169	magnoro
14	afriagna	66	jabora	118	mangady	170	lalagna
15	nagnakory	67	atody	119	milomagno	171	tanety
16	aza	68	ampondo	120	magnembagna	172	mena
17	jiaby	69	rambo	121	mandeha	173	maitso
18	fontry	70	volomborogno	122	avy	174	fondragna
19	vitsy	71	volo	123	mandry	175	fotsy
20	hely	72	loha	124	mantontry	176	mahintigny
21	hafa	73	sofigny	125	mitsangana	177	aligny
22	araiky	74	maso	126	mihodigny	178	andro
23	aroa	75	horogno	127	lavo	179	taogno
24	telo	76	vava	128	magname	180	mafana
25	efatra	77	nify	129	mitagna	181	manintsy
26	dimy	78	lela	130	mitery	182	feno
27	geda	79	angofo	131	mandrokotro	183	vaovao
28	lava	80	tongotro	132	manasa	184	antitry
29	malalaka	81	bokombavitsy	133	mamafa	185	tsara
30	matevigny	82	lohalitry	134	mitifitry	186	ratsy
31	mavesatra	83	tanagna	135	manosiky	187	lo
32	hely	84	helatra	136	manopy	188	maloto
33	fohy	85	kibo	137	manohy	189	mahitsy
34	mahety	86	tsinay	138	manjaitry	190	taboribory
35	matify	87	vozogno	139	magnisa	191	marangitry
36	vaiavy	88	tahezagna	140	magnamabara	192	malomogno
37	lalahy	89	tratra	141	mihira	193	malamadamatra
38	ologno	90	fo	142	misoma	194	malamatra
39	zaza	91	aty	143	mitsilay	195	maigny
40	vady	92	migiaka	144	mivalagna	196	manjary
41	vady	93	mihinagna	145	mivaingagna	197	marikitry
42	niny	94	magnekitry	146	mibohaka	198	lavitry
43	baba	95	minono	147	masova	199	ankahery
44	biby	96	mihika	148	davolagna	200	ankavia
45	lako	97	mandoa	149	lakintagna	201	agny
46	vorogno	98	mitsitro	150	rano	202	agnatiny
47	fandroaka	99	miaigny	151	mahalegny	203	miaraka
48	ahao	100	mimoehy	152	ranomivalagna	204	ndraiky
49	bibilava	101	mahita	153	matsabory	205	izikoa
50	hankangna	102	mitandregny	154	ranomasigny	206	satria
51	kakazo	103	mahay	155	sira	207	agnaragna
52	atiala	104	mandiniky	156	vato		

41 - Tsimihety (Andapa)							
1	zaho	53	kobay	105	mifofogno	157	fasiky
2	anao	54	voandraha	106	matahotro	158	vovoko
3	izy	55	voany	107	mandry	159	tany
4	atsika	56	raviny	108	velogno	160	rondro
5	areo	57	vahiny	109	maty	161	zavogno
6	zare	58	hodinkakazo	110	mamono	162	lagnitry
7	toy	59	folera	111	miady	163	rivotro
8	zegny	60	ahitry	112	mihaza	164	lanezy
9	eto	61	tady	113	mamboboka	165	vaingandrano
10	aroy	62	hoditry	114	mandidy	166	setroko
11	azovy	63	hena	115	mamaky	167	afo
12	ino	64	lio	116	manomboko kiso	168	jofo
13	aiza	65	taholagna	117	magnisiky	169	magnoro
14	afiriagna	66	taviny	118	mangady	170	lalagna
15	akory	67	antoly	119	milomagno	171	tanety
16	aza	68	tandroko	120	magnembana	172	mena
17	jiaby	69	ohy	121	mandeha	173	mahitso
18	fontry	70	volomborogno	122	havy	174	mavo
19	vitsy	71	volo	123	mandry	175	fotsy
20	hely	72	loha	124	mantotry	176	mahintigny
21	hafa	73	sofigny	125	mitsangagna	177	haligny
22	araiky	74	maso	126	mihodigny	178	andro
23	aroa	75	orogno	127	lavo	179	taogno
24	telo	76	vava	128	magname	180	mafana
25	efatra	77	nify	129	mitagna	181	manintsy
26	dimy	78	lela	130	manery	182	feno
27	geda	79	angofo	131	mandrokotro	183	vaovao
28	lava	80	tongotro	132	manasa	184	hantitry
29	malalaka	81	vavitsy	133	mamafa	185	tsara
30	matevigny	82	lohalitry	134	mitifitry	186	ratsy
31	mavesatra	83	tagnagna	135	manosiky	187	lo
32	hely	84	helatra	136	manipy	188	maloto
33	fohy	85	boko	137	mamehy	189	mahitsy
34	mahety	86	tsinay	138	manjaitry	190	taboribory
35	matify	87	ambozogno	139	magnisa	191	maragnitry
36	vaiavy	88	tahezagna	140	mivolagna	192	malomogno
37	lalahy	89	tratra	141	mihira	193	malamadamatra
38	ologno	90	fo	142	misoma	194	legny
39	zaza	91	aty	143	mitsingevegna	195	maigny
40	vady	92	migiaka	144	mivalagna	196	mety
41	vady	93	mihinana	145	mandry	197	mariny
42	mama	94	magnekitry	146	mibohoka	198	lavitry
43	papa	95	minono	147	masova	199	ankavanagna
44	kaka	96	mihaka	148	davolagna	200	ankavia
45	laoko	97	mandoa	149	lakintagna	201	agny
46	vorogno	98	mitsitro	150	rano	202	agnatiny
47	amboa	99	miaigny	151	oragnandro	203	miaraka
48	hao	100	mimoehy	152	tegnarano	204	ndreky
49	bibilava	101	mahita	153	farihy	205	raha
50	hankagna	102	mitandregny	154	ranomasigny	206	satria
51	kakazo	103	mahay	155	sira	207	agnaragna
52	atiala	104	mieritreritra	156	vato		

42 - Nosy Boraha (Ambodifotatra)

1	zaho	53	kobay	105	mifofogno	157	fasiky
2	ana	54	voankazo	106	matahotro	158	vovoko
3	izy	55	vihy	107	mandry	159	tany
4	atsika	56	ravigny	108	velogno	160	miha
5	anare	57	vahitry	109	maty	161	zavogno
6	zare	58	oditry kakazo	110	mamono	162	langitry
7	ito	59	folaira	111	miady	163	rivotro
8	zainy	60	ahitry	112	mitadia	164	ranomandry
9	aketo	61	tady	113	mamely	165	laglasy
10	aka	62	oditry	114	manapaka	166	setroko
11	zovy	63	hena	115	mamaky	167	afo
12	ino	64	ra	116	manomboko antsy	168	lavenogno
13	aiza	65	taholagna	117	mikiky	169	magnoro
14	afiriagna	66	jabora	118	mangady	170	lalambe
15	karakory	67	antody	119	milomagno	171	tanety
16	tsy	68	tandroko	120	magnaibagna	172	mena
17	ijiaby	69	ohy	121	mandeha	173	maitso
18	fontry	70	volomborogno	122	avy	174	mavo
19	eliely	71	randragna	123	mandry	175	fotsy
20	vitsy	72	loha	124	mantotry	176	maintigny
21	afa	73	tadigny	125	mitsangana	177	aligna
22	akaiky	74	maso	126	mihodigna	178	andro
23	aroa	75	orogno	127	lavo	179	taogno
24	telo	76	vava	128	magnamia	180	mafana
25	efatra	77	nify	129	mitantagna	181	mangatsiaka
26	dimy	78	lela	130	tiregna	182	feno
27	maventy	79	angofo	131	mikosoko	183	vao
28	lava	80	ongotro	132	manasa	184	antitry
29	malalaka	81	vavitsy	133	mamafa	185	tsara
30	matevigny	82	lohalitry	134	mitifitry	186	ratsy
31	mavesatra	83	tagnana	135	magnatosika	187	lo
32	hely	84	ailatra	136	mambalabala	188	maloto
33	fohy	85	votraka	137	manohy	189	mahitsy
34	mahety	86	tsinay	138	manjetra	190	boribory
35	manify	87	tenda	139	magnisa	191	marangitry
36	viavy	88	taezagna	140	mivolagna	192	malomo
37	lalahy	89	tratra	141	mihira	193	malamatra
38	ologno	90	fo	142	midola	194	legny
39	zaza	91	aty	143	miempo	195	maigny
40	vady	92	midroko	144	mikoriagna	196	manjary
41	vady	93	mihinagna	145	mandry	197	mariny
42	reny	94	magnekitry	146	mibohaka	198	lavitry
43	ray	95	mitsentsitry	147	masova	199	havanagna
44	biby	96	mandrora	148	davolagna	200	avia
45	loko	97	mandoa	149	kintagna	201	agny
46	vorogno	98	misotro	150	rano	202	agnatiny
47	kiva	99	miaigny	151	oran andro	203	amin ny
48	ho	100	mimoehy	152	renirano	204	ndraiky
49	bibilava	101	mahita	153	dobo	205	koa
50	ankagna	102	maharegny	154	ranomasigna	206	satria
51	kakazo	103	mahay	155	sira	207	agnarana
52	atiala	104	mieritreritry	156	vato		

43 - Bara (Beroroha)

1	iaho	53	kobay	105	magnembo	157	fasy
2	anao	54	vihikazo	106	matahotsy	158	bo
3	i	55	vihy	107	matory	159	tany
4	asika	56	raviny	108	velo	160	raho
5	anareo	57	vahatsy	109	maty	161	zavo
6	reo	58	hodikazo	110	mamono	162	lagnitsy
7	toy	59	flera	111	miady	163	rivotsy
8	zay	60	akata	112	miremby	164	fanala
9	eto	61	hosy	113	mamofoky	165	ranomandry
10	ao	62	hoditsy	114	manapaky	166	setroky
11	ia	63	hena	115	mandidy	167	afo
12	ino	64	ra	116	mitomboky mesa	168	lavenoky
13	aia	65	taola	117	magnihy	169	magnodo
14	mbia	66	sabora	118	mihady	170	lala
15	hakognaia	67	atody	119	milagno	171	vohitsy
16	tsy	68	tsifa	120	mitily	172	mena
17	aby	69	ohy	121	mandeha	173	metso
18	maro	70	volomboro	122	avy	174	vogny
19	sasany	71	volo	123	matory	175	foty
20	kedikedy	72	loha	124	mitoboky	176	mety
21	hafa	73	sofy	125	mitsanga	177	hariva
22	raiky	74	maso	126	miody	178	andro
23	roy	75	oro	127	lavo	179	tao
24	telo	76	vava	128	magnome	180	mafana
25	efatsy	77	hy	129	mita	181	manitsy
26	dimy	78	lela	130	manery	182	feno
27	bevata	79	hoho	131	akasoky	183	vao
28	lava	80	tomboky	132	manasa	184	atitsy
29	malalaky	81	randro	133	mamafa	185	soa
30	matevy	82	lohaliky	134	mitifitsy	186	raty
31	mavetratsy	83	tagna	135	mandrosy	187	lo
32	madiniky	84	elatsy	136	manoraky	188	maloto
33	boribory	85	troky	137	mamehy	189	mahity
34	tery	86	tsinay	138	mitrebiky	190	boribory
35	matify	87	vozo	139	magnisaky	191	maragnitsy
36	apela	88	lambosy	140	magnambara	192	dombo
37	anahoada	89	tratra	141	miatsa	193	malama
38	olo	90	fo	142	misa	194	le
39	zaza	91	aty	143	mihefo	195	maiky
40	vady	92	mino	144	mikoria	196	mety
41	vady	93	mihina	145	mandry	197	mariny
42	reny	94	magnehitsy	146	mibotinaky	198	lavitsy
43	ray	95	mitsetsitsy	147	masoandro	199	havana
44	biby	96	mandrora	148	vola	200	havia
45	fia	97	mandoa	149	vasia	201	agny
46	voro	98	mitsoky	150	rano	202	agnaty
47	amboa	99	miay	151	ora	203	amin ny
48	hao	100	mihehy	152	vavarano	204	sy
49	bibilava	101	mahita	153	ranovory	205	laha
50	haka	102	mahare	154	ranomasy	206	satria
51	hazo	103	mahay	155	sira	207	agnara
52	ala	104	mieritseritsy	156	vato		

44 - Sakalava (Miandrivazo)

1	zaho	53	kobay	105	mahare imbo	157	fasiky
2	iha	54	vihinkazo	106	matahotsy	158	botany
3	iy	55	vihiny	107	matory	159	tany
4	tsika	56	raviny	108	velo	160	raho
5	nareo	57	vahatsy	109	maty	161	zavo
6	reo	58	holinkazo	110	mamono	162	lagnitry
7	ty	59	vonikazo	111	mialy	163	rivotry
8	zay	60	bozaky	112	mihaza	164	ranomandry
9	eto	61	tady	113	mamango	165	gilasy
10	ao	62	hoditra	114	manapaky	166	setroky
11	ia	63	hena	115	mizara	167	motro
12	ino	64	ra	116	mitomboky meso	168	lavenoky
13	aia	65	taola	117	magnihy	169	magnoro
14	ombia	66	taviny	118	mihady	170	lala
15	ino zay	67	adody	119	mandagno	171	bongo
16	tsy	68	tandroky	120	mitsidina	172	mena
17	iaby	69	rambo	121	mandeha	173	maintso
18	maro	70	volomboro	122	tonga	174	vogny
19	kelikely	71	volo	123	matory	175	fotsy
20	kely	72	loha	124	mipetraky	176	mainty
21	hafa	73	sofy	125	mitsanga	177	matognaly
22	raiky	74	maso	126	mihodiky	178	andro
23	roe	75	oro	127	mietota	179	tao
24	telo	76	vava	128	magnome	180	mafana
25	efatsy	77	nify	129	mifampita	181	manintsy
26	dimy	78	lela	130	manandaitsy	182	feno
27	bevata	79	hoho	131	magnakasoky	183	vaovao
28	lava	80	tomboky	132	manasa	184	antitsy
29	malalaky	81	ranjo	133	mamafa	185	soa
30	matevy	82	lohaliky	134	mitifitsy	186	raty
31	mavesatsy	83	tagna	135	manosiky	187	lo
32	kely	84	elatra	136	mitoraky	188	maloto
33	boribory	85	troky	137	mamatotsy	189	mahity
34	tery	86	tsinay	138	manjaitsy	190	boribory
35	manify	87	vozo	139	magnisa	191	maragnitsy
36	ampela	88	lamosy	140	magnambara	192	dombo
37	lelahy	89	tratra	141	mihira	193	malama
38	olo	90	fo	142	mihisa	194	le
39	zaza	91	aty	143	mihefo	195	maiky
40	valy	92	misotro	144	maria	196	mety
41	valy	93	homana	145	mandry	197	mariny
42	neny	94	manaikitry	146	boboky	198	lavitsy
43	baba	95	mifiky	147	masoandro	199	havana
44	biby	96	mandrehoky	148	boara	200	havia
45	fia	97	mandoa	149	basia	201	agny
46	voro	98	mitsoky	150	rano	202	agnatiny
47	alika	99	miay	151	ora	203	amin ny
48	hao	100	mihehy	152	vavarano	204	sy
49	bibilava	101	mahita	153	ranovory	205	raha
50	hanka	102	mijanjy	154	riaky	206	satria
51	hazo	103	mahay	155	sira	207	agnara
52	ala	104	mieritreritsy	156	vato		

45 - Vezo (Morondava)							
1	zaho	53	kobay	105	magnimbo	157	vasiky
2	iha	54	voakazo	106	matahotry	158	lemboky
3	i	55	vihy	107	miroro	159	tany
4	tsika	56	ravy	108	velo	160	hiboky
5	nareo	57	fototsy	109	maty	161	zavo
6	rozy	58	holinkazo	110	mamono	162	lagnitry
7	toy	59	folera	111	mialy	163	tsioky
8	zany	60	akata	112	mihaza	164	ando
9	eto	61	taly	113	mamango	165	gilasy
10	atia	62	holiny	114	manapaky	166	setroky
11	ia	63	hena	115	mizara	167	motro
12	ino	64	lio	116	mitomboky meso	168	lavenoky
13	aia	65	taola	117	mikiky	169	miketriky
14	ombia	66	vonjaky	118	mihaly	170	lala
15	akory	67	atoly	119	milagno	171	vohitry
16	tsy	68	tsifa	120	mitily	172	mena
17	iaby	69	hoy	121	madea	173	maintso
18	maro	70	volomboro	122	avy	174	vogny
19	tsiampeampe	71	volo	123	miroro	175	foty
20	tsiampe	72	loha	124	mitoboky	176	mainty
21	hafa	73	sofy	125	mitsanga	177	matognaly
22	raiky	74	maso	126	mihodiky	178	atoandro
23	roe	75	oro	127	miantonta	179	tao
24	telo	76	vava	128	magnome	180	mafana
25	efatry	77	hy	129	mitazo	181	manintsy
26	dimy	78	lela	130	mitery	182	atriky
27	bevata	79	ho	131	mikasoky	183	vao
28	lava	80	tomboky	132	manasa	184	antitry
29	malalaky	81	ranjo	133	mamafa	185	soa
30	matevy	82	ongotry	134	mitifitry	186	raty
31	mavesatry	83	tagna	135	manosiky	187	mantsy
32	maliniky	84	elatry	136	magnoroky	188	maloto
33	bory	85	sarotry	137	mamatotry	189	mahity
34	mafitsy	86	tinay	138	mitrebiky	190	boribory
35	manify	87	vozo	139	magnisaky	191	maragnitry
36	ampela	88	lambosy	140	mivola	192	madomo
37	johary	89	tratra	141	mihira	193	madoso
38	olo	90	fo	142	mihisa	194	legna
39	aja	91	aty	143	mihafo	195	maiky
40	valy	92	mihino	144	mikorisa	196	mety
41	valy	93	mihina	145	mandrike	197	mariny
42	neny	94	magnehitry	146	mivonto	198	lavitry
43	baba	95	mitsitsiky	147	masoandro	199	havana
44	mbiby	96	mandreoky	148	vola	200	havia
45	fia	97	mandoa	149	basia	201	agny
46	voro	98	mitsoky	150	rano	202	agnaty
47	alika	99	miay	151	ora	203	aminy
48	hao	100	mihehy	152	vavarano	204	sy
49	bibilava	101	mahita	153	ranovory	205	raha
50	soko	102	mijanjy	154	ranomasy	206	satria
51	hazo	103	mahay	155	sira	207	agnara
52	ala	104	mandiniky	156	vato		

46 - Bara (Ihosy)

1	iaho	53	kobay	105	magnemboky	157	fasiky
2	hanao	54	voakazo	106	matatahosy	158	bo
3	i	55	vihy	107	mandry	159	tany
4	sika	56	ravy	108	velo	160	raho
5	nareo	57	faka	109	maty	161	zavo
6	andreo	58	hoditrazo	110	mamono	162	lanisy
7	toy	59	vonkazo	111	miady	163	rivosy
8	zay	60	akata	112	mamita	164	ranomandry
9	etoa	61	hoso	113	mamofoky	165	glasy
10	ato	62	hoditsy	114	manapaky	166	setroky
11	ia	63	hena	115	manilaky	167	afo
12	ino	64	lio	116	mitomboky mesa	168	lavenoky
13	aia	65	taola	117	mikihy	169	magnoro
14	ombia	66	sabora	118	mihady	170	lala
15	akory	67	atody	119	milagno	171	vohisy
16	tsy	68	sifa	120	misidy	172	mena
17	aby	69	ohony	121	mandeha	173	meso
18	maro	70	volomboro	122	avy	174	vony
19	kidikidy	71	volo	123	mandry	175	fosy
20	kidy	72	loha	124	mitoboky	176	minty
21	hafa	73	sofy	125	mitsanga	177	aly
22	raiky	74	maso	126	mihotaky	178	andro
23	roy	75	oro	127	megebo	179	tao
24	telo	76	vava	128	manolosy	180	mafana
25	efatry	77	nihy	129	mitata	181	manisy
26	dimy	78	lela	130	mitery	182	feno
27	foloay	79	hoho	131	manatasoky	183	vao
28	abo	80	tomboky	132	manasa	184	antisy
29	malalaky	81	ranjo	133	mamafa	185	soa
30	matevy	82	pokopoky	134	mitifosy	186	raty
31	mazefasy	83	tana	135	manosiky	187	lo
32	madiniky	84	elasy	136	manoraky	188	motaky
33	pohy	85	troky	137	mamehy	189	mahisy
34	pisa	86	tinay	138	mitreboky	190	bory
35	fisaky	87	vozo	139	manisa	191	maragnisy
36	ampela	88	lambosy	140	miroho	192	dombo
37	rangahy	89	tratra	141	miatsa	193	malama
38	olo	90	fo	142	mihisa	194	le
39	zaza	91	aty	143	mihefo	195	maiky
40	vady	92	mino	144	mikoria	196	mety
41	vady	93	mihina	145	mandry	197	mariny
42	endry	94	magnehisy	146	mibosina	198	lavisy
43	aba	95	mitsetsiky	147	masoandro	199	havagna
44	biby	96	mandrehoky	148	vola	200	havia
45	fia	97	mandoha	149	kinta	201	agny
46	voro	98	mifioky	150	rano	202	agnaty
47	amboa	99	miay	151	ora	203	miray
48	hao	100	mihehy	152	vavarano	204	sy
49	bibilava	101	magnety	153	atara	205	laha
50	haka	102	mijy	154	ranomasy	206	satria
51	hazo	103	hay	155	sira	207	agnara
52	ala	104	mieriserisy	156	vato		

47 - Tsimihety (Antsohihy)							
1	zaho	53	koboay	105	matandregny	157	fasika
2	ana	54	voankazo	106	matahotro	158	jofo
3	izy	55	voaniny	107	mandry	159	tany
4	antsika	56	ravigny	108	velogno	160	rondro
5	are	57	fakany	109	maty	161	zavogno
6	zare	58	hodinkazo	110	mamono	162	langitry
7	to	59	folera	111	miady	163	rovitro
8	zany	60	ahitry	112	mijoko	164	ranomivaingana
9	aketo	61	tady	113	mamiko	165	ranomivaingana
10	aka	62	hoditry kakazo	114	manapaka	166	setroko
11	zovy	63	hena	115	mamaky	167	motro
12	ino	64	lio	116	magnatroboky	168	jofo
13	akeza	65	taholagna	117	mikiky	169	mandoro
14	aforiagna	66	jaborany	118	mangady	170	lalagna
15	magnankory	67	antody	119	milomoagno	171	tendrombohitry
16	tsy	68	tandroko	120	magnembagna	172	mena
17	jiaby	69	rambo	121	mandeha	173	mahintso
18	fontry	70	volomborogno	122	navy	174	fondragna
19	vitsy	71	volo	123	mandry	175	fotsy
20	hely	72	loha	124	mantontry	176	mahitigny
21	hafa	73	sofigny	125	mitsangana	177	haligny
22	araiky	74	maso	126	mihodigny	178	mazava
23	roa	75	orogno	127	lavo	179	taogno
24	telo	76	vava	128	magname	180	mahevoko
25	efatra	77	nify	129	mitantagna	181	manintsy
26	dimy	78	lela	130	mitery	182	feno
27	geda	79	angofo	131	mikosoko	183	vovo
28	lava	80	tongotro	132	manasa	184	hantitry
29	malalaka	81	bokombavitsy	133	mamafa	185	tsara
30	matevigny	82	lohalitry	134	mitifitry	186	ratsy
31	mavesatry	83	tanagna	135	manosiky	187	lo
32	hely	84	elatra	136	manopy	188	maloto
33	fohy	85	kibo	137	mamatotro	189	mahitsy
34	mahety	86	tsinay	138	manjaitry	190	boribory
35	matify	87	vozogno	139	magnisa	191	marangitry
36	vaiavy	88	lamosigny	140	mikoragna	192	dombo
37	lalahy	89	tratra	141	mihira	193	mahitsy
38	ologno	90	fo	142	misoma	194	malamatra
39	zaza	91	aty	143	mitsingevagna	195	maigny
40	vady	92	migiaka	144	mikoriagna	196	manjary
41	vady	93	mihinagna	145	mivaingagna	197	marikitry
42	niny	94	magnekitry	146	mibotsigny	198	lavitry
43	baba	95	minono	147	masova	199	ankavanagna
44	biby	96	mandrehoko	148	volagna	200	ankavia
45	lako	97	mandoa	149	lakintagna	201	agny
46	vorogno	98	mitsotra	150	rano	202	agnatiny
47	fandroaka	99	miaigny	151	oragna	203	amin ny
48	ahao	100	mimoehy	152	renindrano	204	ndreky
49	bibilava	101	mahita	153	mantsabory	205	iziko
50	hankagna	102	mitandregny	154	ranomasigny	206	satria
51	kakazo	103	mahay	155	sira	207	agnaragna
52	hatiala	104	mandiniky	156	vato		

48 - Merina (Maevatanana)

1	zaho	53	kibay	105	mifofona	157	fasika
2	anao	54	voankazo	106	manahy	158	vovoka
3	izy	55	voa	107	matory	159	tany
4	atsika	56	ravina	108	mivelona	160	rahona
5	ianareo	57	faka	109	maty	161	zavona
6	izireo	58	hodikazo	110	mamono	162	lanitra
7	ity	59	felana	111	miady	163	rivotra
8	izany	60	ahitra	112	mihaza	164	ranomandry
9	eto	61	tady	113	mikapoka	165	vongandrano
10	ery	62	hoditra	114	manapaka	166	setroka
11	iza	63	hena	115	mamaky	167	afo
12	ino	64	lio	116	manindrona	168	lavenona
13	aiza	65	taolana	117	mikiky	169	mandoro
14	ovina	66	tavina	118	mandavaka	170	lalana
15	ahoana	67	atody	119	milomano	171	tendrombohitra
16	aza	68	tandroka	120	manidina	172	mena
17	jiaby	69	rambo	121	mandeha	173	maitso
18	maro	70	volomborona	122	avy	174	mavo
19	vitsy	71	volo	123	mandry	175	fotsy
20	kely	72	loha	124	mipetraka	176	mainty
21	hafa	73	sofina	125	mitsangana	177	alina
22	ray	74	maso	126	mihodina	178	mantsana
23	roa	75	orona	127	mianjera	179	taona
24	telo	76	vava	128	manome	180	mafana
25	efatra	77	nify	129	mitazona	181	manintsy
26	dimy	78	lela	130	mipotsitra	182	feno
27	ngeza	79	hoho	131	manakasoka	183	vaovao
28	lava	80	tongotra	132	manasa	184	matoy
29	malalaka	81	ranjo	133	mamafa	185	tsara
30	matevina	82	lohalika	134	mitifitra	186	ratsy
31	mazefatra	83	tanana	135	manosika	187	lo
32	kely	84	elatra	136	mitoraka	188	maloto
33	fohy	85	kibo	137	mamatotra	189	mahitsy
34	tery	86	tsinay	138	manjaitra	190	boribory
35	manify	87	vozona	139	manisa	191	maranitra
36	vehivavy	88	lamosina	140	miteny	192	dombo
37	lehilahy	89	tratra	141	mihira	193	malama
38	olona	90	fo	142	milalao	194	mando
39	zaza	91	aty	143	mitsinkafona	195	maina
40	vady	92	misotro	144	mikoriana	196	mety
41	vady	93	mihinana	145	mandry	197	akaiky
42	neny	94	manaikitra	146	mibontsina	198	lavitra
43	dada	95	minono	147	masoandro	199	havanana
44	biby	96	mandrehoka	148	volana	200	havia
45	fia	97	mandoha	149	kintana	201	any
46	vorona	98	mitsoka	150	rano	202	anaty
47	alika	99	miaina	151	orana	203	miaraka
48	hao	100	mihomehy	152	renirano	204	ary
49	bibilava	101	mijery	153	mantsabory	205	raha
50	kankana	102	mihaino	154	ranomasina	206	satria
51	hazo	103	mahay	155	sira	207	anarana
52	ala	104	mieritreritra	156	vato		

49 - Sakalava (Besalampy)							
1	zao	53	kobay	105	magnimbo	157	fasiky
2	iha	54	vihinkazo	106	misalasala	158	bo
3	i	55	vihy	107	mandry	159	tany
4	tsika	56	ravy	108	mivelo	160	raho
5	nareo	57	vaha	109	maty	161	zavo
6	reo	58	holikazo	110	mamono	162	lagnitry
7	ty	59	flera	111	mialy	163	tsioky
8	zay	60	akata	112	miaza	164	lanezy
9	eto	61	taly	113	mamango	165	glasy
10	ery	62	hoditry	114	manapaky	166	setroky
11	ia	63	hena	115	mamaky	167	motro
12	ino	64	lio	116	mitroboky	168	laveno
13	aia	65	tola	117	mikiky	169	mandoro
14	ombia	66	jabora	118	mandavaky	170	lala
15	manakory	67	atoly	119	milomagno	171	vohitry
16	ka	68	tandroky	120	mitily	172	mena
17	iaby	69	rambo	121	mandeha	173	maitso
18	maro	70	volomboro	122	avy	174	mavo
19	kely	71	volo	123	matoritory	175	foty
20	kely	72	loha	124	mipetraky	176	mainty
21	hafa	73	sofy	125	mitsanga	177	erignaly
22	raiky	74	maso	126	mihodiky	178	antoandro
23	roy	75	oro	127	lavo	179	tao
24	telo	76	vava	128	magnome	180	mafana
25	efatry	77	nify	129	mita	181	manintsy
26	dimy	78	lela	130	mipotsiky	182	feno
27	maventy	79	ho	131	magnasoky	183	vao
28	lava	80	tomboky	132	manasa	184	matoy
29	malalaky	81	ranjo	133	mamafa	185	tsara
30	matevy	82	lohaliky	134	mitifitry	186	raty
31	mavesatry	83	tagna	135	manosiky	187	lo
32	kely	84	elatry	136	mitoraky	188	maloto
33	fohy	85	kibo	137	mamehy	189	mahity
34	tsihomby	86	tinay	138	manjaitry	190	boribory
35	matify	87	vozo	139	magnisaky	191	maragnitry
36	ampela	88	lamosy	140	misafa	192	madomo
37	lelahy	89	tratra	141	mihira	193	malamalama
38	olo	90	fo	142	mihisa	194	pitsaky
39	zaza	91	aty	143	miefo	195	maiky
40	vady	92	mino	144	mitobaky	196	omby
41	vady	93	mihina	145	mandry	197	mariny
42	neny	94	magnetry	146	mibontso	198	lavitry
43	baba	95	minono	147	masoandro	199	ankavana
44	biby	96	mandreoky	148	boara	200	ankavia
45	fia	97	mandoa	149	kinta	201	agny
46	voro	98	mitsoky	150	rano	202	agnaty
47	amboa	99	miaigna	151	ora	203	miaraky
48	hao	100	mihehy	152	vavarano	204	ndraiky
49	bibilava	101	mikasa	153	dobo	205	lafa
50	hanka	102	mijanjy	154	ranosira	206	satria
51	hazo	103	mahay	155	sira	207	agnara
52	ala	104	mieritreritry	156	vato		

50 - Betsimisaraka (Tanambao Manampotsy)

1	zaho	53	fioka	105	mifofogna	157	fasina
2	ano	54	vonkazo	106	vaka	158	vovoka
3	izy	55	vihiny	107	mandry	159	tany
4	antsena	56	ravina	108	velona	160	raona
5	andreo	57	vahany	109	maty	161	zavona
6	zareo	58	oditra kakazo	110	mamono	162	lagnitra
7	ity	59	folera	111	miady	163	rivotra
8	zany	60	ahitra	112	mitadia	164	ranomandry
9	akito	61	afotra	113	mamango	165	ranomandry
10	ako	62	hoditra	114	manapaka	166	toisina
11	zovy	63	ena	115	mizara	167	afo
12	ino	64	ra	116	manombok ansy	168	lavinina
13	akeza	65	tolagna	117	mikiky	169	magnoro
14	oviana	66	taviny	118	mangady	170	araby
15	ino	67	atody	119	mandagno	171	tavirana
16	sy	68	tandroka	120	mirigna	172	mena
17	jiaby	69	volombody	121	mandiaa	173	miso
18	be	70	volomborogna	122	tonga	174	mavo
19	visivisy	71	randrana	123	mandry	175	fosy
20	bitika	72	loa	124	mikomaka	176	mintina
21	hafa	73	tadigny	125	mideragna	177	alina
22	reka	74	maso	126	miodigna	178	andro
23	roy	75	orogna	127	lavo	179	taona
24	telo	76	vava	128	magnomia	180	mafana
25	efatra	77	nify	129	mitondra	181	mangasiaka
26	dimy	78	lela	130	mamia	182	feno
27	lebe	79	angofo	131	mampikasoka	183	vovo
28	lava	80	tongotra	132	manasa	184	antitra
29	malalaka	81	ladirana	133	mamafa	185	sara
30	mativina	82	loalitra	134	mitifitra	186	rasy
31	mavesatra	83	tagnana	135	manosika	187	lo
32	bitika	84	elatra	136	mambalavala	188	maloto
33	foika	85	kibo	137	mamatotra	189	mahisy
34	tery	86	olika	138	manzitra	190	boribory
35	manify	87	atoka	139	mangnisa	191	marangitra
36	viavy	88	tambokoka	140	mizaka	192	malombona
37	lilahy	89	tratra	141	miira	193	malady
38	olona	90	fo	142	milalo	194	mando
39	zaza	91	aty	143	miboagna	195	megna
40	vady	92	miinana	144	midoroka	196	mety
41	vady	93	miinana	145	mivengana	197	amarivo
42	mama	94	magnifatra	146	mamoky rivotra	198	lavitra
43	papa	95	misisitra	147	masoandro	199	avanana
44	biby	96	mandreoka	148	volana	200	avia
45	loka	97	mandoa	149	kintana	201	agny
46	vorogna	98	misotra	150	rano	202	agnatiny
47	kiva	99	miegna	151	orana	203	miarak amin ny
48	o	100	mimehy	152	tegnarano	204	sy
49	bibilava	101	magnitigna	153	farihy	205	raha
50	ankagna	102	mitino	154	ranomasina	206	satria
51	kakazo	103	mae	155	sira	207	agnarana
52	atiala	104	mieritreritra	156	vato		

51 - Sihanaka (Morarano Chrome)							
1	za	53	firitsoka	105	mifofona	157	fasika
2	enao	54	vonkazo	106	matahotra	158	jofo
3	izy	55	vony	107	matory	159	tany
4	tsikana	56	ravina	108	vielona	160	rahona
5	nareo	57	faka	109	maty	161	zavona
6	ry zareo	58	hodikazo	110	mamono	162	lanitra
7	ty	59	voninkiazo	111	miady	163	rivotra
8	zany	60	ahatra	112	mihaza	164	ranomandry
9	eto	61	tady	113	mandaroka	165	ranomandry
10	ao	62	hoditra	114	manapaka	166	setroka
11	iza	63	hena	115	mamaky	167	afo
12	inona	64	ra	116	manomboka antsy	168	lavenina
13	aiza	65	tolana	117	mikiky	169	mandoro
14	ovina	66	matavy	118	mitombana	170	lalana
15	ahona	67	atody	119	milomano	171	tendrombohitra
16	tsy	68	tandroka	120	manembana	172	mena
17	daholo	69	rambo	121	mande	173	maitso
18	bietsaka	70	volomborona	122	avy	174	mavo
19	vitsy	71	volo	123	matory	175	fotsy
20	bitika	72	loha	124	mipetraka	176	mainty
21	hafa	73	sofina	125	mijaridina	177	alina
22	ray	74	maso	126	miodina	178	andro
23	roa	75	orona	127	lavo	179	tona
24	telo	76	vava	128	manome	180	mafana
25	iefatra	77	nify	129	mitantana	181	mangatsieka
26	dimy	78	lela	130	manery	182	fieno
27	ngeda	79	angofo	131	manakasoka	183	vaovao
28	lava	80	tongotra	132	manasa	184	antitra
29	malalaka	81	ranjo	133	mamafa	185	tsara
30	matevina	82	lohalika	134	mitifitra	186	ratsy
31	mavesatra	83	tanana	135	manosika	187	lo
32	kitika	84	ielatra	136	manary	188	maloto
33	fohy	85	ventre	137	mamatotra	189	mahitsy
34	tery	86	tsinay	138	manjaitra	190	boribory
35	manify	87	atoka	139	manisa	191	matsiko
36	vaiavy	88	lamosina	140	miteny	192	dombo
37	lela	89	tratra	141	mihira	193	malama
38	olona	90	fo	142	milalao	194	mando
39	zaza	91	aty	143	mitsingievana	195	maina
40	vady	92	misotro	144	mikoriana	196	miety
41	vady	93	minana	145	mivaingana	197	akaiky
42	mama	94	manaikitra	146	mibontsina	198	lavitra
43	dada	95	mitsetsitra	147	masoandro	199	ankavanana
44	biby	96	mandriehoaka	148	volana	200	ankavia
45	trondro	97	mandoa	149	kintana	201	any
46	vorona	98	mitsoka	150	rano	202	anaty
47	amboa	99	miaina	151	orana	203	miaraka
48	hao	100	mimehy	152	renirano	204	sy
49	bibilava	101	mahita	153	farihy	205	raha
50	felika	102	mihaino	154	ranomasina	206	satry
51	kakazo	103	mahay	155	sira	207	anarana
52	atiala	104	mieritrieritra	156	vato		

52 - Betsileo (Ambohimahasoa)							
1	aho	53	kobay	105	mamofona	157	fasika
2	agnao	54	voakazo	106	matahotsa	158	vovoka
3	iy	55	voany	107	matory	159	tany
4	antsika	56	ravina	108	velogna	160	rahogna
5	agnareo	57	fakany	109	maty	161	zavona
6	zareo	58	hodikazo	110	mamono	162	lagnitsa
7	ito	59	voninkiazo	111	miady	163	rivotsa
8	izay	60	bozaka	112	miremby	164	ranomandry
9	eto	61	tady	113	manjera	165	ranomandry
10	ao	62	hoditsa	114	manapaka	166	setroka
11	ia	63	hena	115	mamaky	167	afo
12	ina	64	ra	116	manomboka mesa	168	lavenina
13	aia	65	taolagna	117	mikiky	169	magnoro
14	viana	66	vondraka	118	mihady	170	lalagna
15	manahoana	67	atody	119	mandagno	171	tendrombohitsa
16	tsa	68	tandroka	120	manidigna	172	mena
17	aby	69	rambo	121	mandeha	173	maitso
18	betsaka	70	volomborogna	122	avy	174	mavo
19	vitsy	71	volo	123	matory	175	fotsy
20	kely	72	loha	124	mitoetsa	176	mainty
21	hafa	73	sofigna	125	mitsangana	177	aligna
22	raika	74	maso	126	mihodigna	178	andro
23	roa	75	orogna	127	lavo	179	taogna
24	telo	76	vava	128	manome	180	mafana
25	efatsa	77	nify	129	mitantana	181	mmanara
26	dimy	78	lela	130	manery	182	feno
27	libe	79	hoho	131	magnasoka	183	vaovao
28	lava	80	tongotsa	132	manasa	184	antitsa
29	malalaka	81	voavitsy	133	mamafa	185	soa
30	matevina	82	lohalika	134	mitifitsa	186	ratsy
31	mavetsatsa	83	tagnana	135	manosika	187	lo
32	kitika	84	elatsa	136	magnary	188	maloto
33	fohy	85	troka	137	mandrohy	189	mahitsy
34	tery	86	tsinay	138	manjaitsa	190	boribory
35	manify	87	vozogna	139	magnisa	191	maragnitsa
36	ampela	88	voho	140	mitarogna	192	bodo
37	lela	89	tratra	141	mihira	193	malama
38	ona	90	fo	142	milaolao	194	mando
39	zaza	91	aty	143	mitsingevagna	195	maigna
40	vady	92	misotro	144	mikoriagna	196	mety
41	vady	93	mihinana	145	mivongana	197	mariny
42	neny	94	magnekitsa	146	mibotsigna	198	lavitsa
43	baba	95	mitroka	147	masoandro	199	ankavanana
44	biby	96	mandrehoka	148	volagna	200	ankavia
45	trondro	97	mandoa	149	kintana	201	agny
46	vorogna	98	mitsioka	150	rano	202	agnaty
47	amboa	99	miaigna	151	oragna	203	miaraka
48	hao	100	mihehy	152	vavarano	204	sy
49	bibilava	101	mahita	153	farihy	205	raha
50	hakana	102	mitaino	154	ranomasina	206	satria
51	hazo	103	mahay	155	sira	207	agnarana
52	ala	104	mieritseritsa	156	vato		

53 - Betsimisaraka (Maroantsetra)

1	zaha	53	kobe	105	manimbolo	157	fasika
2	ano	54	voankazo	106	matahotro	158	vovoka
3	izy	55	voa	107	mandry	159	tany
4	atsika	56	ravigny	108	velogna	160	rahona
5	andre	57	faka	109	maty	161	zavona
6	zare	58	hoditry kakazo	110	mamono	162	lanitra
7	ty	59	voninkazo	111	miady	163	rivotra
8	zegny	60	ahitra	112	mihaza	164	ranomandry
9	eto	61	tady	113	mamango	165	ranomandry
10	ako	62	hoditry	114	manapaka	166	setroko
11	zovy	63	hena	115	mamaky	167	afo
12	ino	64	ra	116	mamira antsy	168	lavenogno
13	aiza	65	taholagna	117	mirapy	169	mandoro
14	oviagna	66	taviny	118	mangady	170	lalagna
15	ahoana	67	atody	119	miomana	171	tanety
16	tsy	68	tandroka	120	magnembagna	172	mena
17	jiaby	69	rambony	121	mandeha	173	mahitso
18	maro	70	volomborogno	122	avy	174	asaka
19	vitsivitsy	71	randragna	123	mandry	175	fotsy
20	hely	72	loha	124	mipetraka	176	mainty
21	hafa	73	tadigny	125	mitsangana	177	aligny
22	araiky	74	maso	126	mihodigna	178	andro
23	aro	75	orogna	127	lavo	179	taona
24	telo	76	vava	128	magnamia	180	mafana
25	efatra	77	nify	129	mitantana	181	mangatsiaka
26	dimy	78	lela	130	tiregny	182	feno
27	maventy	79	angofo	131	akasoko	183	vaovao
28	lava	80	tongotra	132	manasa	184	antitra
29	malalaka	81	vavitsy	133	mamafa	185	tsara
30	matevina	82	lohalika	134	mitifitra	186	ratsy
31	mavesatra	83	tagnana	135	manosika	187	lo
32	hely	84	elatra	136	mambalabala	188	maloto
33	fohy	85	kibo	137	mamehy	189	mahitsy
34	tery	86	tsinay	138	manjaitra	190	boribory
35	manify	87	ambozogna	139	magnisa	191	marangitry
36	viavy	88	tahezagna	140	mivolagna	192	donto
37	lalahy	89	tratra	141	mihira	193	malamatra
38	olo	90	fo	142	midola	194	mando
39	zaza	91	aty	143	mitsingevagna	195	maina
40	vady	92	migiaka	144	mikoriagna	196	mety
41	vady	93	mihinana	145	mandry	197	marikitry
42	reny	94	magnekitra	146	mibontsigna	198	lavitra
43	baba	95	mifiaka	147	masova	199	havanana
44	biby	96	mandrehoka	148	davolagna	200	havia
45	loko	97	mandoa	149	lakitagna	201	agny
46	vorogno	98	mifiko	150	rano	202	agnatiny
47	amboa	99	miaigny	151	oragnandro	203	amin ny
48	ho	100	mimoehy	152	loharano	204	sy
49	bibilava	101	mahita	153	farihy	205	raha
50	kakagna	102	mitandregny	154	ranomasina	206	satria
51	kakazo	103	mahe	155	sira	207	agnarana
52	atiala	104	mieritreritry	156	vato		

54 - Merina (Analavory)							
1	zahakony	53	kobay	105	mamofona	157	fasika
2	enao	54	vokazo	106	matahoatra	158	vovoka
3	izy	55	vo	107	matory	159	tany
4	tsika	56	ravina	108	velona	160	rahoana
5	nareo	57	faka	109	maty	161	zavona
6	zareo	58	hodikazo	110	mamono	162	lanitra
7	ity	59	voninkazo	111	miady	163	rivotra
8	izany	60	vilona	112	miaza	164	ranomandry
9	eto	61	tady	113	mikapoka	165	ranomandry
10	ao	62	hoditra	114	mandidy	166	setroka
11	ia	63	hena	115	mamaky	167	afo
12	inona	64	ra	116	manindrona antsy	168	lavena
13	aiza	65	tolana	117	mikiky	169	mandoro
14	vina	66	taviny	118	mangady	170	lalana
15	aona	67	atody	119	milomano	171	tendrombohitra
16	tsy	68	tandroka	120	manidina	172	mena
17	rehetra	69	rambo	121	mandeha	173	maintso
18	bediabe	70	volomborona	122	avy	174	mavo
19	vitsivitsy	71	volo	123	matory	175	fotsy
20	kely	72	lo	124	mipetraka	176	menty
21	afa	73	sofina	125	mitsangana	177	alina
22	ray	74	maso	126	miodina	178	andro
23	roa	75	orona	127	mianjera	179	taona
24	telo	76	vava	128	manome	180	mafana
25	efatra	77	nify	129	mitantana	181	mangatsika
26	dimy	78	lela	130	manery	182	feno
27	lebe	79	hoho	131	manakasoka	183	vaovao
28	lava	80	tongotra	132	manasa	184	antitra
29	malalaka	81	ranjo	133	mamafa	185	tsara
30	matevina	82	lohalika	134	mitifitra	186	ratsy
31	mazevatra	83	tanana	135	manosika	187	lo
32	kely	84	elatra	136	mitoraka	188	maloto
33	fo	85	kibo	137	mame	189	mahintsy
34	tery	86	tsinay	138	manjaitra	190	boribory
35	manify	87	vozona	139	manisa	191	maranitra
36	vevavy	88	soroka	140	milaza	192	dombo
37	lela	89	tratra	141	mihira	193	malama
38	olona	90	fo	142	milalao	194	mando
39	zaza	91	aty	143	mitsingevana	195	maina
40	vady	92	misotro	144	mikorina	196	mety
41	vady	93	minana	145	mandry	197	akaiky
42	reny	94	manekitra	146	mibontsina	198	lavitra
43	ray	95	minono	147	masoandro	199	avanana
44	biby	96	mandrehoaka	148	volana	200	avia
45	trondro	97	mando	149	kintana	201	any
46	vorina	98	mitsoka	150	rano	202	anaty
47	alika	99	miaina	151	orana	203	amin ny
48	hao	100	mime	152	renirano	204	sy
49	bibilava	101	maita	153	farihy	205	ra
50	kankana	102	miaino	154	ranomasina	206	satry
51	hazo	103	mahay	155	sira	207	anarana
52	ala	104	mieritreritra	156	vato		

55 - Betsimisaraka (Sahavato)

1	zao	53	fioka	105	mifofogna	157	fasina
2	ano	54	vonkazo	106	vaka	158	vovoka
3	ize	55	vihine	107	mandre	159	tane
4	ansena	56	ravina	108	velona	160	rahogna
5	anareo	57	vahatra	109	mate	161	zavona
6	zareo	58	hoditra kakazo	110	mamono	162	lagnitra
7	ty	59	vognonkazo	111	miade	163	rivotra
8	zane	60	ahitra	112	mitadia	164	ranomandre
9	aketo	61	tade	113	mamango	165	ranomandre
10	ako	62	hoditra	114	manapaka	166	toesina
11	zovy	63	hena	115	mamake	167	afo
12	ino	64	ra	116	mampitrobaka anse	168	lavenona
13	akaza	65	tolagna	117	magnisika	169	magnoro
14	oviena	66	tavine	118	mangade	170	lalana
15	aona	67	tode	119	mandagno	171	dongona
16	tse	68	tandroka	120	miborogna	172	mena
17	rehetra	69	volombody	121	mandia	173	meso
18	maro	70	volomborogna	122	ave	174	mavo
19	visivise	71	randrana	123	mandre	175	fose
20	bitaka	72	loha	124	mipetraka	176	mintina
21	afa	73	tadigne	125	misangana	177	alina
22	reka	74	maso	126	miodigna	178	andro
23	roe	75	orogna	127	lavo	179	tona
24	telo	76	vava	128	magnomia	180	mafana
25	efatra	77	nife	129	mitana	181	mangaseka
26	dime	78	lela	130	manere	182	feno
27	lebe	79	vanzakoho	131	magnakasoka	183	vovo
28	lava	80	ongotra	132	manasa	184	antitra
29	malalaka	81	randro	133	mamafa	185	sara
30	matevina	82	loalitra	134	mitifitra	186	rase
31	mavesatra	83	tagnana	135	manosika	187	lo
32	bitaka	84	elatra	136	manambioka	188	maloto
33	foika	85	kibo	137	mamehe	189	mahise
34	tere	86	olika	138	manzetra	190	boribore
35	manife	87	vozogna	139	magnisa	191	maragnitra
36	viave	88	tambokoka	140	mizaka	192	dombo
37	lelae	89	tratra	141	mihira	193	malady
38	olona	90	fo	142	milalao	194	mando
39	zaza	91	ate	143	miempo	195	megna
40	vade	92	omana	144	mandia	196	mete
41	vade	93	omana	145	mandre	197	amarivo
42	endre	94	magnifatra	146	mivonto	198	alavitra
43	iaba	95	misesitra	147	mosoandro	199	avanana
44	bibe	96	mandrehoka	148	volana	200	avia
45	loka	97	mandoa	149	kintana	201	akagne
46	vorogna	98	misotra	150	rano	202	agnate
47	kiva	99	miegna	151	orana	203	amin ny
48	ho	100	mimehe	152	tegnarano	204	se
49	biblava	101	mahita	153	ose	205	raha
50	vike	102	miteno	154	ranomasina	206	satria
51	kakazo	103	mahe	155	sira	207	agnarana
52	atiala	104	mieritreritra	156	vato		

56 - Mahafaly (Ejeda)

1	iaho	53	kobay	105	manimbo	157	faseke
2	iriha	54	voakantae	106	mavaka	158	deboke
3	ireke	55	voa	107	miroro	159	tane
4	itika	56	ravene	108	miay	160	rahogne
5	inareo	57	fototse	109	mate	161	zavogne
6	iareo	58	holinketae	110	mandenta	162	lagnitse
7	intoy	59	vognene	111	mialy	163	tioke
8	ezao	60	ahetse	112	mipay	164	ranomandry
9	etoa	61	taly	113	mamango	165	rano mivangagne
10	ao	62	holitse	114	mandily	166	setroke
11	ia	63	hena	115	manilake	167	afo
12	ino	64	lio	116	mitomboke meso	168	lavenoke
13	aia	65	taola	117	mikiake	169	magnoro
14	nombia	66	vondrake	118	mihaly	170	lala
15	akore	67	atoly	119	milagno	171	vohitse
16	tsie	68	tsifa	120	mitily	172	mena
17	iaby	69	ohie	121	mamindra	173	maitso
18	maro	70	volomboro	122	avy	174	vogne
19	tsiampe	71	volo	123	mandre	175	foty
20	kele	72	loha	124	mitomboke	176	mainte
21	hafa	73	sofy	125	mitsanga	177	halike
22	raike	74	fijere	126	mihodike	178	andro
23	roe	75	oro	127	mitonta	179	tao
24	telo	76	vava	128	magnome	180	mae
25	efatse	77	nife	129	mitanjake	181	manitsy
26	lime	78	lela	130	mipiritsy	182	feno
27	befoloay	79	hoho	131	mampikasoke	183	vaovao
28	lava	80	tomboke	132	manasa	184	antetse
29	malalake	81	ranjo	133	mipio	185	soa
30	mateve	82	ongotse	134	mitifitse	186	raty
31	mavesatse	83	tagna	135	manoseke	187	lo
32	kele	84	elatse	136	mitorake	188	maloto
33	bory	85	troke	137	mandrohy	189	mahity
34	maifitse	86	tinay	138	mitrebeke	190	boribory
35	matify	87	vozo	139	magnisake	191	maragnitse
36	ampisafe	88	lambosy	140	mivola	192	sihanake
37	zaranjaka	89	aragna	141	miantsa	193	malama
38	ndaty	90	arofo	142	mihisa	194	lende
39	ajaja	91	ate	143	mihafo	195	maike
40	valy	92	mino	144	midorasitse	196	mete
41	valy	93	mitava	145	mandreke	197	marine
42	nene	94	mitifatse	146	mibokinake	198	lavitse
43	baba	95	mitsintsike	147	andro	199	havagna
44	biby	96	mandrehoke	148	vola	200	havia
45	fia	97	mandoa	149	vasia	201	agne
46	voro	98	mitioke	150	rano	202	agnate
47	amboa	99	miay	151	ora	203	amie
48	hao	100	mihehe	152	vavaramo	204	noho
49	bibilava	101	mahisake	153	ranovory	205	naho
50	soko	102	mijanjy	154	riake	206	satria
51	hetae	103	mahafantatse	155	sira	207	agnara
52	ala	104	mandinike	156	vato		

57 - Antanosy (Belamoty)							
1	zaho	53	kobay	105	manofo	157	fasiny
2	hanao	54	voakazo	106	mataosy	158	vovotany
3	izy	55	vihiny	107	mandry	159	tany
4	sika	56	raviny	108	velo	160	rahony
5	handreo	57	vahany	109	maty	161	mika
6	rireo	58	holikazo	110	mamono	162	lanignitsy
7	tiho	59	ravikazo	111	mialy	163	rivosy
8	izao	60	akata	112	mamita	164	ranomandriy
9	etaho	61	taly	113	manjera	165	vaingadrano
10	ao	62	amgozy	114	manapaky	166	setroky
11	ija	63	hena	115	mamaky	167	afo
12	ino	64	lio	116	mitomboky mesa	168	lavenoky
13	eza	65	taola	117	mikiky	169	manoro
14	nombia	66	vondrany	118	mihaly	170	lala
15	manoakory	67	atoly	119	milano	171	vohidry
16	sizy	68	sifa	120	mitily	172	mena
17	iziaby	69	ohihy	121	mandeha	173	maintso
18	maro	70	volomboro	122	avy	174	mavo
19	kidikidy	71	volo	123	mandry	175	fosy
20	tsiampy	72	loha	124	mitoboky	176	meity
21	hafa	73	sofy	125	misanga	177	hariva
22	raiky	74	maso	126	miodiky	178	andro
23	ro	75	oro	127	manegny	179	tao
24	telo	76	vava	128	magnome	180	mafana
25	efasy	77	nify	129	mita	181	manara
26	dimy	78	lela	130	mitery	182	feno
27	befoloay	79	vazankoho	131	mikasiky	183	vaovao
28	lava	80	tomboky	132	manasa	184	antitsy
29	malalaky	81	randro	133	mamafa	185	soa
30	mateviny	82	pokopoko	134	mitifisy	186	rasy
31	mavesasy	83	tana	135	manosiky	187	lo
32	keliky	84	elany	136	manary	188	maloto
33	fohiky	85	troky	137	mamehy	189	mahitsy
34	tery	86	sinay	138	mandraitsy	190	foiky
35	matify	87	vozo	139	magnisaky	191	maranitsy
36	ampela	88	lambosy	140	mizaka	192	dofoky
37	lelahy	89	tratra	141	mibeko	193	malama
38	olo	90	fo	142	mihisa	194	mando
39	zaza	91	aty	143	mihafo	195	maiky
40	valy	92	mino	144	mipitiky	196	mety
41	valy	93	mihina	145	mandriky	197	marikitsy
42	neny	94	manifatry	146	mironto	198	lavisy
43	papa	95	minono	147	masoandro	199	akavana
44	biby	96	mandrehoky	148	vola	200	akavia
45	fia	97	mandoa	149	kitany	201	agny
46	voro	98	mifioky	150	rano	202	anaty
47	amboa	99	maiky	151	ora	203	amin ny
48	hao	100	homehy	152	renirano	204	sy
49	bibilava	101	mahita	153	farihy	205	nohiha
50	soko	102	miteno	154	riaky	206	satria
51	hazo	103	mahay	155	sira	207	agnara
52	ala	104	mieriseritry	156	vato		

58 - Sihanaka (Andilamena)							
1	za	53	anjera	105	manimbolo	157	fasika
2	ianao	54	voankazo	106	matahotra	158	jofo
3	izy	55	voniny	107	matory	159	tany
4	antsika	56	ravina	108	velona	160	rondro
5	anareo	57	fakany	109	maty	161	zavina
6	zareo	58	vatrankazo	110	mamono	162	langitra
7	ity	59	vonikazo	111	miady	163	rivotra
8	zany	60	ahitra	112	mitazana	164	ranomandry
9	eto	61	tady	113	mitaroka	165	ranomandry
10	ao	62	hoditra	114	manaobale	166	hetona
11	iza	63	babaty	115	mamaky	167	afo
12	inona	64	ra	116	manatsatoka antsy	168	lavenona
13	aiza	65	taolana	117	mikiky	169	magnoro
14	ovina	66	taviny	118	mangady	170	lalana
15	ahoana	67	atody	119	miseka	171	tanety
16	tsy	68	tandroka	120	manidina	172	mena
17	daholo	69	rambo	121	mandeha	173	maitso
18	maro	70	volomborona	122	avy	174	hasaka
19	vitsivitsy	71	volo	123	mandry	175	fotsy
20	kely	72	loha	124	mitoetra	176	mainty
21	hafa	73	sofina	125	mitsangana	177	alina
22	iray	74	maso	126	mihodina	178	andro
23	roa	75	orona	127	lavo	179	taona
24	telo	76	vava	128	manome	180	mafana
25	efatra	77	nify	129	mitantana	181	mangatseka
26	dimy	78	lela	130	manery	182	feno
27	ngeda	79	angofo	131	mampikasoka	183	vaovao
28	lava	80	tongotra	132	manasa	184	antitra
29	malalaka	81	vovintsy	133	mamafa	185	tsara
30	matevina	82	lohalika	134	mitifitra	186	ratsy
31	mavesatra	83	tanana	135	manosika	187	lo
32	kely	84	elatra	136	manoraka	188	maloto
33	pota	85	kibo	137	mamatotra	189	mahitsy
34	tery	86	tsinay	138	manjaitra	190	boribory
35	matify	87	vozona	139	magnisa	191	maragnitra
36	vahiavy	88	lamosina	140	milaza	192	malomina
37	lehilahy	89	tratra	141	mihira	193	malama
38	olona	90	fo	142	midalao	194	mando
39	zaza	91	kobony	143	mitsikembo	195	maigna
40	vady	92	misotro	144	mijiregna	196	mety
41	vady	93	mihinana	145	mandry	197	akaiky
42	niny	94	manaikitra	146	mivonto	198	avitra
43	baba	95	mifiaka	147	masova	199	ankavanana
44	biby	96	mandreraka	148	volana	200	ankavia
45	trondro	97	mando	149	kintana	201	any
46	vorona	98	mitsoka	150	rano	202	anaty
47	amboa	99	miaina	151	orana	203	amin ny
48	hao	100	mihomehy	152	renirano	204	sy
49	bibilava	101	mahita	153	farihy	205	raha
50	vika	102	mihaino	154	ranomasina	206	satria
51	kakazo	103	manampahaizana	155	sira	207	anarana
52	ala	104	mieritreritra	156	vato		

59 - Antandroy (Tsihombe)

1	iraho	53	kobay	105	mihantsogne	157	faseke
2	irehe	54	voankazo	106	matahotse	158	deboke
3	ireke	55	voa	107	miroro	159	tane
4	ntika	56	ravigne	108	velogne	160	rahogne
5	nareo	57	vahae	109	mate	161	zono
6	iareo	58	holinkata	110	mamono	162	lagnitse
7	toy	59	folera	111	mialy	163	tioke
8	zane	60	ahitse	112	mihaza	164	ranomandre
9	etoa	61	taly	113	mijera	165	ranomandre
10	ao	62	holitse	114	mandily	166	setroke
11	ia	63	hena	115	mamaky	167	afo
12	ino	64	lio	116	mitomboke	168	lavenoke
13	aia	65	taolagne	117	mikiky	169	magnoro
14	mbia	66	vondrae	118	magnaly	170	lalagne
15	akore	67	atoly	119	milomagno	171	vohitse
16	tsie	68	tsifa	120	mitiligne	172	mena
17	iaby	69	ramboe	121	magnavelo	173	maintso
18	tsiefa	70	volomborogne	122	avy	174	mavo
19	tsiampe	71	maroy	123	mandre	175	foty
20	kede	72	loha	124	toboke	176	mainte
21	hafa	73	sofigne	125	mitsangagne	177	halegne
22	raike	74	maso	126	miodigne	178	andro
23	roe	75	orogne	127	tota	179	taogne
24	telo	76	vava	128	magnome	180	mafana
25	efatse	77	nife	129	mitagne	181	manintsy
26	dime	78	lela	130	manere	182	feno
27	bey	79	hoho	131	mampikasoke	183	vaovao
28	lava	80	tomboke	132	manasa	184	antetse
29	malalake	81	ranjogne	133	mamafa	185	fanjaka
30	matevegne	82	lohaleke	134	mitifitse	186	raty
31	mavesatse	83	tagnagne	135	manoseke	187	lo
32	kede	84	elatse	136	mitorake	188	maloto
33	foheke	85	troke	137	mamefe	189	mahitsy
34	tere	86	tsinay	138	manjaitse	190	bontolitoly
35	manify	87	vozogne	139	magnisa	191	maragnitse
36	ampela	88	lambosigne	140	mitalily	192	malomogne
37	lahilahy	89	tratra	141	mihira	193	malama
38	ndaty	90	arofo	142	mihisa	194	legne
39	ajaja	91	ate	143	misintogne	195	maike
40	valy	92	minogne	144	mijiririagne	196	mete
41	valy	93	mihinagne	145	mandre	197	marine
42	rene	94	manifatse	146	mivonto	198	lavitse
43	rae	95	mamiake	147	masoandro	199	havanagne
44	biby	96	mandrehoke	148	volagne	200	havia
45	fiagne	97	mandoa	149	vasiagne	201	agne
46	vorogne	98	mifioke	150	rano	202	agnatee
47	amboa	99	miaigne	151	oragne	203	amigne
48	hao	100	mihehe	152	renerano	204	naho
49	bibilava	101	mahatrea	153	sihanake	205	naho
50	soko	102	mitranogne	154	riake	206	satria
51	hatae	103	mahay	155	sira	207	agnaragne
52	ala	104	mandineke	156	vato		

60 - Sakalava (Belon'i Tsiribihina)

1	zaho	53	kobay	105	magnimbo	157	fasiky
2	iha	54	voankazo	106	mavozo	158	vovoky
3	izy	55	vininy	107	miroro	159	tany
4	tsika	56	raviny	108	velo	160	rahona
5	nareo	57	vahany	109	mate	161	zavo
6	rozy	58	holin kazo	110	mamono	162	lanitse
7	ity	59	folera	111	mialy	163	rivotry
8	izany	60	ahitsy	112	mamandriky	164	ranomandry
9	eto	61	osy	113	mamango	165	ranomandry
10	ao	62	nofotsy	114	manapake	166	setroky
11	ia	63	hena	115	mamaky	167	motro
12	ino	64	lio	116	tsofoko meso	168	lavenike
13	aia	65	taola	117	mikiky	169	magnoro
14	ombia	66	tsabora	118	mangaly	170	lala
15	manahoa	67	atody	119	milomagno	171	dongo
16	tsy	68	tsifa	120	mitily	172	mena
17	rehetra	69	rambo	121	mandeha	173	maitso
18	maro	70	volomboro	122	avy	174	mavo
19	kelikely	71	volo	123	miroro	175	fotsy
20	madiniky	72	loha	124	midoboka	176	mainty
21	hafa	73	sofy	125	mitsanga	177	hariva
22	raiky	74	maso	126	miholiky	178	andro
23	roe	75	oro	127	lavo	179	tao
24	telo	76	tsihovy	128	manome	180	mafana
25	efatra	77	nify	129	mitanta	181	magnitsy
26	dimy	78	lela	130	manery	182	feno
27	bevata	79	hoho	131	mikosiky	183	vaovao
28	haba	80	tomboky	132	manasa	184	antitry
29	malalaky	81	ranjo	133	mamafa	185	soa
30	matevy	82	tsipoko	134	mitififtsy	186	raty
31	mavesatra	83	tagna	135	manosika	187	lo
32	kely	84	elatsy	136	mitoraky	188	maloto
33	boribory	85	kibo	137	mamehy	189	mahaty
34	maifitsy	86	tinay	138	manjaitry	190	boribory
35	matify	87	vozo	139	magnisaky	191	maranitry
36	ampela	88	lambosy	140	miteny	192	dombo
37	nahoda	89	tratra	141	mihira	193	malama
38	olo	90	fo	142	miisa	194	le
39	aja	91	aty	143	miafo	195	maiky
40	valy	92	mino	144	mikoria	196	mety
41	valy	93	mihina	145	mandry	197	mariniky
42	njaray	94	magnekitry	146	boboka	198	lavitsy
43	aba	95	minono	147	masoandro	199	havana
44	biby	96	mandrehoky	148	volana	200	havia
45	fia	97	mandoa	149	vasia	201	agny
46	voro	98	mifioky	150	rano	202	agnaty
47	amboa	99	miay	151	ora	203	amin ny
48	hao	100	mihehy	152	renirano	204	sy
49	bibilava	101	manenty	153	dobo	205	raha
50	kanka	102	mijanjy	154	ranomasy	206	satria
51	hazo	103	mahay	155	sira	207	agnara
52	ala	104	mametsivetsy	156	vato		
