# Supplementary material for: Dialects of Madagascar
Source: PLoS One. 2020 Oct 2;15(10):e0240170. doi: 10.1371/journal.pone.0240170 (PMC7531839; doi:10.1371/journal.pone.0240170)
Supplement: S1 Table — The name of the dialects (ethnicities), the name of the Towns/Villages where dialects were collected together with their coordinates. Latitude and longitude are given in degrees complete of decimals, therefore, the two digits after point do not represent seconds but degree’s decimals. (PDF) [file pone.0240170.s002.pdf]

Table S1: Ethnicities, towns and coordinates

#	Ethnicity	Town	Coordinates	
1	Sakalava	Ambanja	-13.67	48.33
2	Sihanaka	Ambatondrazaka	-17.87	48.42
3	Antankarana	Ambilobe	-13.20	49.05
4	Antandroy	Ambovombe	-25.17	46.08
5	Mahafaly	Ampanihy	-24.68	44.73
6	Mikea	Ampoakafo	-22.67	43.75
7	Betsimisaraka	Antalaha	-14.88	50.27
8	Merina	Antananarivo	-18.90	47.52
9	Bara	Betroka	-23.28	46.08
10	Zafisoro	Farafangana	-22.80	47.82
11	Betsimisaraka	Fenoarivo-Est	-17.38	49.40
12	Betsileo	Fianarantsoa	-21.43	47.08
13	Betsimisaraka	Mahanoro	-19.88	48.80
14	Sakalava	Maintirano	-18.07	44.02
15	Sakalava	Mahajanga	-15.72	46.32
16	Antaimoro	Manakara	-22.10	48.00
17	Antambohoaka	Mananjary	-21.22	48.33
18	Tsimihety	Mandritsara	-15.83	48.82
19	Masikoro	Miary	-23.30	43.72
20	Sakalava	Morondava	-20.28	44.32
21	Antanosy	Tolagnaro	-25.03	46.98
22	Vezo	Toliara	-23.34	43.67
23	Antaisaka	Vangaindrano	-23.32	47.60
24	Antankarana	Vohemar	-13.37	50.00
25	Betsileo	Ambositra	-20.52	47.25
26	Betsileo	Ambalavao	-21.83	46.92
27	Antanalana	Itampolo	-24.68	43.95
28	Vezo	Morombe	-21.74	43.36
29	Antanosy	Bezaha	-23.50	44.50
30	Tanala	Ifanadiana	-21.28	47.63
31	Bara	Ranohira	-22.43	45.35
32	Antanalana	Manorofify	-23.52	43.97
33	Antandroy	Toliara	-23.34	43.67
34	Antanalana	Anakao	-23.67	43.65
35	Betsimisaraka	Marolambo	-20.05	48.12
36	Betsimisaraka	Antsiranana	-12.27	49.28
37	Betsimisaraka	Brickaville	-18.82	49.07
38	Betsimisaraka	Toamasina	-18.15	49.42
39	Betsimisaraka	Mananara	-16.17	49.77
40	Tsimihety	Mampikony	-16.09	47.64
41	Tsimihety	Andapa	-14.67	49.65
42	Nosy Boraha	Ambodifotatra	-17.00	49.85
43	Bara	Beroroha	-21.67	45.17
44	Sakalava	Miandrivazo	-19.56	45.45
45	Vezo	Morondava	-20.28	44.32
46	Bara	Ihosy	-22.40	46.12
47	Tsimihety	Antsohihy	-14.88	47.98
48	Merina	Maevatanana	-16.95	46.83
49	Sakalava	Besalampy	-16.75	44.47
50	Betsimisaraka	Tanambao Manampotsy	-19.48	48.57
51	Sihanaka	Morarano Chrome	-17.75	48.17
52	Betsileo	Ambohimahasoa	-21.10	47.22
53	Betsimisaraka	Maroantsetra	-15.43	49.75
54	Merina	Analavory	-18.58	46.43
55	Betsimisaraka	Sahavato	-20.60	48.33
56	Mahafaly	Ejeda	-24.35	44.52
57	Antanosy	Belamoty	-23.55	44.80
58	Sihanaka	Andilamena	-17.02	48.58
59	Antandroy	Tsihombe	-25.32	45.48
60	Sakalava	Belon'i Tsiribihina	-19.70	44.55

The name of the dialects (ethnicities), the name of the Towns/Villages where dialects were collected toghether with their coordinates. Latitude and longitude are given in degrees complete of decimals, therefore, the two digits after point do not represent seconds but degree's decimals.
