# Supplementary material for: Dialects of Madagascar
Source: PLoS One. 2020 Oct 2;15(10):e0240170. doi: 10.1371/journal.pone.0240170 (PMC7531839; doi:10.1371/journal.pone.0240170)
Supplement: S2 Table — This table contains the N × N upper triangular matrix whose entries are the N(N − 1)/2 = 1770 lexical distances D(α, β) between all pairs of languages. (PDF) [file pone.0240170.s003.pdf]

	Sakalava (Ambanja)	Sihanaka (Ambatondrazaka)	Antankarana (Ambilobe)	Antandroy (Ambovombe)	Mahafaly (Ampanihy)	Mikea (Ampoakafo)	Betsimisaraka (Antalaha)	Merina (Antananarivo)	Bara (Betroka)	Zafisoro (Farafangana)	Betsimisaraka (Fenoarivo-Est)	Betsileo (Fianarantsoa)	Betsimisaraka (Mahanoro)	Sakalava (Maintirano)	Sakalava (Mahajanga)	Antaimoro (Manakara)	Antambohoaka (Mananjary)	Tsimihety (Mandritsara)	Masikoro (Miary)	Sakalava (Morondava)	Antanosy (Tolagnaro)	Vezo (Toliara)	Antaisaka (Vangaindrano)	Antankarana (Vohemar)	Betsileo (Ambositra)	Betsileo (Ambalavao)	Antanalana (Itampolo)	Vezo (Morombe)	Antanosy (Bezaha)	Tanala (Ifanadiana)	Bara (Ranohira)	Antanalana (Manorofify)	Antandroy (Toliara)	Antanalana (Anakao)	Betsimisaraka (Marolambo)	Betsimisaraka (Antsiranana)	Betsimisaraka (Brickaville)	Betsimisaraka (Toamasina)	Betsimisaraka (Mananara)	Tsimihety (Mampikony)	Tsimihety (Andapa)	Nosy Boraha (Ambodifotatra)	Bara (Beroroha)	Sakalava (Miandrivazo)	Vezo (Morondava)	Bara (Ihosy)	Tsimihety (Antsohihy)	Merina (Maevatanana)	Sakalava (Besalampy)	Betsimisaraka (Tanambao M.)	Sihanaka (Morarano Chrome)	Betsileo (Ambohimahasoa)	Betsimisaraka (Maroantsetra)	Merina (Analavory)	Betsimisaraka (Sahavato)	Mahafaly (Ejeda)	Antanosy (Belamoty)	Sihanaka (Andilamena)	Antandroy (Tsihombe)	Sakalava (Belon'i Tsiribihina)
**Sakalava (Ambanja)**	0.000	0.412	0.130	0.510	0.493	0.481	0.225	0.385	0.407	0.393	0.295	0.397	0.375	0.394	0.324	0.368	0.365	0.224	0.423	0.419	0.422	0.448	0.383	0.112	0.369	0.417	0.498	0.460	0.458	0.393	0.416	0.465	0.497	0.460	0.422	0.229	0.392	0.369	0.310	0.208	0.250	0.298	0.420	0.395	0.421	0.443	0.267	0.355	0.346	0.421	0.406	0.389	0.302	0.400	0.423	0.497	0.435	0.387	0.437	0.401
**Sihanaka (Ambatondrazaka)**		0.000	0.415	0.457	0.461	0.496	0.357	0.181	0.377	0.361	0.341	0.263	0.327	0.383	0.322	0.294	0.253	0.373	0.421	0.421	0.369	0.398	0.368	0.399	0.257	0.308	0.452	0.426	0.405	0.290	0.406	0.451	0.463	0.434	0.329	0.333	0.308	0.290	0.361	0.366	0.337	0.339	0.387	0.356	0.405	0.406	0.332	0.201	0.368	0.318	0.144	0.257	0.264	0.213	0.333	0.468	0.370	0.208	0.410	0.366
**Antankarana (Ambilobe)**			0.000	0.501	0.476	0.470	0.250	0.380	0.404	0.403	0.324	0.388	0.398	0.379	0.340	0.386	0.365	0.243	0.409	0.408	0.412	0.436	0.400	0.146	0.388	0.416	0.487	0.450	0.451	0.403	0.413	0.460	0.494	0.445	0.430	0.244	0.397	0.378	0.305	0.260	0.258	0.325	0.412	0.399	0.408	0.443	0.293	0.365	0.367	0.427	0.420	0.384	0.314	0.398	0.423	0.484	0.424	0.386	0.441	0.409
**Antandroy (Ambovombe)**				0.000	0.332	0.440	0.480	0.423	0.402	0.464	0.503	0.395	0.522	0.414	0.460	0.441	0.450	0.485	0.387	0.408	0.386	0.335	0.461	0.506	0.406	0.394	0.342	0.375	0.399	0.460	0.413	0.338	0.263	0.340	0.501	0.461	0.504	0.483	0.514	0.509	0.476	0.481	0.401	0.407	0.397	0.418	0.495	0.441	0.444	0.515	0.470	0.402	0.459	0.441	0.497	0.297	0.396	0.466	0.231	0.405
**Mahafaly (Ampanihy)**					0.000	0.344	0.470	0.420	0.337	0.439	0.500	0.412	0.498	0.334	0.447	0.437	0.437	0.483	0.298	0.309	0.343	0.269	0.405	0.478	0.416	0.362	0.204	0.297	0.347	0.459	0.344	0.221	0.342	0.247	0.502	0.479	0.504	0.499	0.496	0.486	0.473	0.498	0.337	0.347	0.328	0.366	0.481	0.421	0.365	0.517	0.467	0.414	0.463	0.452	0.481	0.170	0.349	0.451	0.279	0.342
**Mikea (Ampoakafo)**						0.000	0.472	0.459	0.367	0.463	0.502	0.440	0.523	0.323	0.473	0.472	0.475	0.465	0.221	0.274	0.384	0.248	0.433	0.467	0.463	0.396	0.320	0.255	0.390	0.481	0.360	0.284	0.418	0.298	0.525	0.475	0.526	0.526	0.499	0.488	0.468	0.507	0.324	0.346	0.289	0.384	0.488	0.463	0.371	0.534	0.488	0.432	0.485	0.467	0.506	0.337	0.395	0.484	0.379	0.343
**Betsimisaraka (Antalaha)**							0.000	0.320	0.391	0.373	0.215	0.327	0.348	0.401	0.334	0.324	0.321	0.151	0.422	0.417	0.394	0.448	0.367	0.236	0.323	0.364	0.479	0.462	0.407	0.348	0.404	0.457	0.456	0.435	0.376	0.255	0.325	0.303	0.222	0.156	0.154	0.228	0.394	0.378	0.427	0.421	0.208	0.305	0.342	0.355	0.347	0.306	0.223	0.341	0.383	0.466	0.389	0.309	0.400	0.382
**Merina (Antananarivo)**								0.000	0.333	0.339	0.300	0.159	0.305	0.347	0.300	0.247	0.224	0.345	0.391	0.391	0.322	0.372	0.314	0.367	0.157	0.238	0.423	0.404	0.366	0.182	0.352	0.405	0.416	0.385	0.302	0.276	0.287	0.254	0.323	0.335	0.297	0.299	0.327	0.285	0.375	0.358	0.306	0.116	0.325	0.293	0.156	0.191	0.197	0.080	0.291	0.420	0.325	0.140	0.350	0.304
**Bara (Betroka)**									0.000	0.294	0.413	0.322	0.425	0.264	0.380	0.304	0.333	0.390	0.253	0.261	0.191	0.287	0.230	0.405	0.341	0.266	0.343	0.309	0.250	0.334	0.102	0.308	0.406	0.277	0.440	0.371	0.428	0.411	0.412	0.402	0.380	0.389	0.166	0.246	0.259	0.187	0.396	0.351	0.269	0.431	0.367	0.317	0.364	0.359	0.428	0.317	0.239	0.357	0.368	0.283
**Zafisoro (Farafangana)**										0.000	0.372	0.340	0.388	0.345	0.399	0.252	0.305	0.383	0.357	0.361	0.326	0.383	0.189	0.405	0.335	0.334	0.436	0.408	0.348	0.291	0.304	0.424	0.450	0.411	0.404	0.379	0.411	0.369	0.377	0.389	0.368	0.351	0.308	0.328	0.345	0.341	0.366	0.357	0.334	0.402	0.351	0.339	0.356	0.345	0.390	0.428	0.341	0.345	0.424	0.336
**Betsimisaraka (Fenoarivo-Est)**											0.000	0.327	0.287	0.399	0.374	0.310	0.292	0.228	0.440	0.430	0.413	0.447	0.359	0.292	0.317	0.365	0.491	0.463	0.419	0.328	0.413	0.477	0.460	0.446	0.342	0.291	0.302	0.247	0.200	0.225	0.190	0.179	0.402	0.376	0.434	0.433	0.235	0.322	0.357	0.301	0.323	0.310	0.212	0.323	0.336	0.496	0.407	0.302	0.425	0.381
**Betsileo (Fianarantsoa)**												0.000	0.340	0.351	0.331	0.265	0.266	0.348	0.373	0.361	0.314	0.367	0.330	0.382	0.147	0.167	0.424	0.398	0.346	0.273	0.329	0.395	0.376	0.377	0.334	0.299	0.330	0.290	0.338	0.351	0.309	0.319	0.302	0.284	0.371	0.342	0.327	0.209	0.329	0.332	0.247	0.163	0.254	0.188	0.342	0.403	0.321	0.232	0.318	0.311
**Betsimisaraka (Mahanoro)**													0.000	0.436	0.354	0.296	0.226	0.367	0.471	0.465	0.416	0.472	0.387	0.375	0.314	0.382	0.499	0.489	0.437	0.323	0.431	0.484	0.495	0.477	0.194	0.353	0.230	0.219	0.312	0.364	0.327	0.276	0.428	0.403	0.457	0.461	0.348	0.324	0.406	0.168	0.300	0.314	0.257	0.317	0.217	0.514	0.417	0.307	0.450	0.429
**Sakalava (Maintirano)**														0.000	0.377	0.366	0.379	0.390	0.211	0.205	0.287	0.256	0.318	0.382	0.339	0.320	0.320	0.277	0.310	0.379	0.253	0.289	0.428	0.273	0.454	0.383	0.453	0.426	0.422	0.408	0.391	0.399	0.244	0.219	0.211	0.294	0.392	0.352	0.236	0.444	0.392	0.360	0.379	0.371	0.461	0.332	0.308	0.370	0.380	0.234
**Sakalava (Mahajanga)**															0.000	0.338	0.308	0.363	0.395	0.383	0.366	0.387	0.389	0.316	0.330	0.360	0.437	0.406	0.403	0.341	0.390	0.438	0.479	0.402	0.370	0.304	0.358	0.357	0.378	0.351	0.338	0.355	0.371	0.357	0.372	0.391	0.336	0.272	0.329	0.366	0.322	0.340	0.313	0.326	0.395	0.448	0.379	0.337	0.425	0.338
**Antaimoro (Manakara)**																0.000	0.212	0.340	0.402	0.393	0.327	0.399	0.261	0.372	0.247	0.306	0.451	0.425	0.368	0.243	0.332	0.432	0.423	0.419	0.311	0.314	0.300	0.267	0.329	0.330	0.317	0.299	0.342	0.344	0.382	0.360	0.312	0.272	0.345	0.301	0.272	0.245	0.249	0.274	0.309	0.437	0.337	0.275	0.379	0.341
**Antambohoaka (Mananjary)**																	0.000	0.340	0.408	0.405	0.340	0.407	0.314	0.361	0.248	0.316	0.454	0.429	0.381	0.245	0.356	0.448	0.449	0.420	0.244	0.307	0.264	0.229	0.326	0.349	0.310	0.292	0.350	0.356	0.392	0.388	0.323	0.247	0.357	0.234	0.241	0.258	0.246	0.250	0.222	0.451	0.347	0.240	0.383	0.357
**Tsimihety (Mandritsara)**																		0.000	0.415	0.409	0.400	0.439	0.375	0.249	0.336	0.381	0.484	0.451	0.414	0.366	0.402	0.455	0.466	0.432	0.401	0.281	0.367	0.307	0.233	0.113	0.143	0.241	0.388	0.376	0.417	0.427	0.194	0.337	0.357	0.377	0.361	0.332	0.256	0.365	0.383	0.477	0.394	0.322	0.405	0.395
**Masikoro (Miary)**																			0.000	0.162	0.256	0.152	0.336	0.418	0.399	0.337	0.265	0.176	0.265	0.405	0.244	0.223	0.398	0.226	0.476	0.416	0.485	0.460	0.438	0.431	0.411	0.426	0.206	0.249	0.144	0.274	0.415	0.402	0.273	0.482	0.426	0.369	0.415	0.407	0.480	0.286	0.284	0.419	0.355	0.244
**Sakalava (Morondava)**																				0.000	0.283	0.195	0.324	0.409	0.381	0.337	0.276	0.209	0.286	0.404	0.254	0.257	0.407	0.215	0.479	0.406	0.466	0.458	0.446	0.420	0.405	0.426	0.208	0.213	0.143	0.263	0.408	0.386	0.248	0.476	0.435	0.380	0.418	0.414	0.476	0.297	0.286	0.426	0.366	0.205
**Antanosy (Tolagnaro)**																					0.000	0.301	0.268	0.407	0.334	0.275	0.349	0.332	0.208	0.352	0.207	0.322	0.416	0.306	0.416	0.394	0.427	0.411	0.418	0.406	0.389	0.391	0.224	0.249	0.277	0.220	0.403	0.344	0.294	0.417	0.363	0.320	0.370	0.354	0.421	0.333	0.189	0.356	0.351	0.273
**Vezo (Toliara)**																						0.000	0.349	0.438	0.390	0.315	0.205	0.060	0.289	0.403	0.276	0.185	0.351	0.178	0.467	0.421	0.475	0.450	0.451	0.445	0.420	0.439	0.259	0.266	0.157	0.288	0.431	0.383	0.291	0.478	0.408	0.369	0.417	0.400	0.460	0.235	0.287	0.411	0.293	0.259
**Antaisaka (Vangaindrano)**																							0.000	0.381	0.316	0.292	0.395	0.378	0.292	0.296	0.237	0.384	0.434	0.376	0.395	0.370	0.384	0.366	0.364	0.370	0.350	0.340	0.258	0.282	0.310	0.255	0.368	0.341	0.286	0.390	0.338	0.330	0.339	0.332	0.376	0.393	0.281	0.325	0.388	0.293
**Antankarana (Vohemar)**																								0.000	0.364	0.419	0.484	0.450	0.440	0.388	0.412	0.456	0.482	0.455	0.400	0.222	0.394	0.363	0.309	0.252	0.258	0.318	0.410	0.389	0.415	0.436	0.293	0.352	0.346	0.399	0.393	0.373	0.293	0.389	0.416	0.484	0.416	0.375	0.417	0.391
**Betsileo (Ambositra)**																									0.000	0.225	0.435	0.422	0.381	0.275	0.343	0.415	0.396	0.398	0.316	0.303	0.322	0.276	0.334	0.328	0.298	0.312	0.329	0.281	0.372	0.353	0.308	0.203	0.336	0.317	0.236	0.186	0.242	0.185	0.318	0.407	0.359	0.224	0.342	0.334
**Betsileo (Ambalavao)**																										0.000	0.377	0.349	0.315	0.304	0.271	0.342	0.377	0.331	0.378	0.357	0.381	0.352	0.379	0.376	0.354	0.361	0.257	0.249	0.331	0.270	0.366	0.270	0.286	0.376	0.301	0.207	0.309	0.269	0.384	0.351	0.287	0.301	0.323	0.289
**Antanalana (Itampolo)**																											0.000	0.238	0.354	0.448	0.340	0.170	0.360	0.194	0.510	0.480	0.498	0.499	0.496	0.481	0.470	0.488	0.326	0.345	0.278	0.335	0.472	0.425	0.355	0.517	0.463	0.418	0.469	0.450	0.494	0.206	0.340	0.445	0.304	0.331
**Vezo (Morombe)**																												0.000	0.315	0.425	0.298	0.212	0.385	0.211	0.486	0.439	0.488	0.471	0.472	0.452	0.434	0.458	0.286	0.305	0.185	0.320	0.440	0.410	0.312	0.497	0.432	0.397	0.439	0.431	0.477	0.264	0.309	0.433	0.319	0.284
**Antanosy (Bezaha)**																													0.000	0.372	0.254	0.336	0.399	0.309	0.446	0.410	0.441	0.434	0.426	0.425	0.389	0.417	0.248	0.279	0.291	0.263	0.417	0.382	0.311	0.426	0.399	0.347	0.400	0.389	0.442	0.344	0.145	0.388	0.362	0.275
**Tanala (Ifanadiana)**																														0.000	0.354	0.438	0.469	0.411	0.332	0.320	0.298	0.298	0.360	0.369	0.330	0.314	0.346	0.319	0.395	0.375	0.339	0.216	0.342	0.321	0.271	0.283	0.274	0.218	0.316	0.453	0.359	0.266	0.412	0.359
**Bara (Ranohira)**																															0.000	0.291	0.410	0.291	0.447	0.394	0.443	0.428	0.421	0.405	0.391	0.391	0.141	0.237	0.249	0.173	0.412	0.371	0.258	0.442	0.386	0.322	0.377	0.377	0.433	0.316	0.242	0.368	0.375	0.279
**Antanalana (Manorofify)**																																0.000	0.344	0.161	0.484	0.448	0.499	0.486	0.485	0.459	0.447	0.460	0.299	0.311	0.261	0.322	0.452	0.411	0.333	0.511	0.449	0.401	0.435	0.433	0.474	0.193	0.325	0.428	0.271	0.312
**Antandroy (Toliara)**																																	0.000	0.367	0.496	0.459	0.505	0.475	0.484	0.468	0.436	0.468	0.399	0.400	0.406	0.409	0.476	0.424	0.424	0.502	0.460	0.371	0.444	0.432	0.479	0.315	0.397	0.453	0.244	0.390
**Antanalana (Anakao)**																																		0.000	0.489	0.434	0.480	0.466	0.461	0.453	0.415	0.440	0.282	0.285	0.242	0.316	0.433	0.390	0.321	0.495	0.439	0.389	0.430	0.415	0.491	0.207	0.309	0.416	0.291	0.284
**Betsimisaraka (Marolambo)**																																			0.000	0.358	0.272	0.234	0.367	0.394	0.367	0.331	0.432	0.407	0.459	0.445	0.379	0.325	0.429	0.164	0.302	0.311	0.281	0.317	0.165	0.501	0.418	0.325	0.441	0.427
**Betsimisaraka (Antsiranana)**																																				0.000	0.335	0.314	0.295	0.285	0.258	0.283	0.373	0.359	0.402	0.411	0.285	0.286	0.339	0.352	0.329	0.319	0.232	0.293	0.353	0.470	0.379	0.307	0.394	0.352
**Betsimisaraka (Brickaville)**																																					0.000	0.245	0.336	0.350	0.330	0.287	0.416	0.400	0.460	0.447	0.325	0.300	0.394	0.217	0.296	0.309	0.259	0.303	0.267	0.499	0.425	0.310	0.449	0.426
**Betsimisaraka (Toamasina)**																																						0.000	0.277	0.315	0.268	0.250	0.399	0.383	0.434	0.438	0.281	0.286	0.409	0.224	0.260	0.275	0.213	0.284	0.245	0.491	0.408	0.286	0.433	0.405
**Betsimisaraka (Mananara)**																																							0.000	0.243	0.192	0.200	0.407	0.393	0.428	0.437	0.245	0.355	0.387	0.330	0.345	0.329	0.225	0.345	0.352	0.508	0.411	0.309	0.437	0.407
**Tsimihety (Mampikony)**																																								0.000	0.151	0.235	0.400	0.377	0.415	0.423	0.125	0.308	0.339	0.371	0.339	0.318	0.261	0.354	0.401	0.484	0.404	0.316	0.411	0.382
**Tsimihety (Andapa)**																																									0.000	0.206	0.377	0.365	0.405	0.404	0.192	0.291	0.341	0.334	0.303	0.291	0.218	0.320	0.370	0.462	0.376	0.296	0.378	0.371
**Nosy Boraha (Ambodifotatra)**																																										0.000	0.368	0.378	0.410	0.415	0.232	0.326	0.359	0.301	0.330	0.316	0.200	0.321	0.329	0.484	0.399	0.315	0.409	0.378
**Bara (Beroroha)**																																											0.000	0.202	0.227	0.181	0.400	0.356	0.256	0.423	0.368	0.304	0.367	0.349	0.422	0.325	0.262	0.366	0.362	0.237
**Sakalava (Miandrivazo)**																																												0.000	0.228	0.264	0.368	0.304	0.231	0.397	0.344	0.288	0.333	0.307	0.402	0.331	0.279	0.324	0.355	0.220
**Vezo (Morondava)**																																													0.000	0.256	0.392	0.375	0.246	0.460	0.409	0.380	0.406	0.402	0.470	0.292	0.287	0.408	0.358	0.242
**Bara (Ihosy)**																																														0.000	0.421	0.359	0.271	0.452	0.395	0.341	0.392	0.385	0.450	0.338	0.260	0.399	0.394	0.267
**Tsimihety (Antsohihy)**																																															0.000	0.295	0.345	0.355	0.318	0.309	0.246	0.328	0.388	0.479	0.394	0.297	0.409	0.367
**Merina (Maevatanana)**																																																0.000	0.277	0.315	0.191	0.230	0.250	0.155	0.329	0.420	0.337	0.207	0.365	0.313
**Sakalava (Besalampy)**																																																	0.000	0.426	0.359	0.330	0.352	0.338	0.428	0.365	0.290	0.355	0.371	0.245
**Betsimisaraka (Tanambao Manampotsy)**																																																		0.000	0.283	0.306	0.267	0.304	0.194	0.517	0.409	0.302	0.455	0.416
**Sihanaka (Morarano Chrome)**																																																			0.000	0.207	0.255	0.171	0.305	0.474	0.366	0.183	0.406	0.358
**Betsileo (Ambohimahasoa)**																																																				0.000	0.249	0.204	0.332	0.404	0.315	0.235	0.342	0.332
**Betsimisaraka (Maroantsetra)**																																																					0.000	0.239	0.283	0.456	0.368	0.220	0.391	0.359
**Merina (Analavory)**																																																						0.000	0.298	0.450	0.347	0.187	0.379	0.327
**Betsimisaraka (Sahavato)**																																																							0.000	0.486	0.420	0.310	0.426	0.415
**Mahafaly (Ejeda)**																																																								0.000	0.350	0.454	0.257	0.331
**Antanosy (Belamoty)**																																																									0.000	0.359	0.359	0.270
**Sihanaka (Andilamena)**																																																										0.000	0.399	0.350
**Antandroy (Tsihombe)**																																																											0.000	0.354
**Sakalava (Belon'i Tsiribihina)**																																																												0.000
