# Supplementary figures and images for: Dialects of Madagascar
Source: PLoS One. 2020 Oct 2;15(10):e0240170. doi: 10.1371/journal.pone.0240170 (PMC7531839; doi:10.1371/journal.pone.0240170)

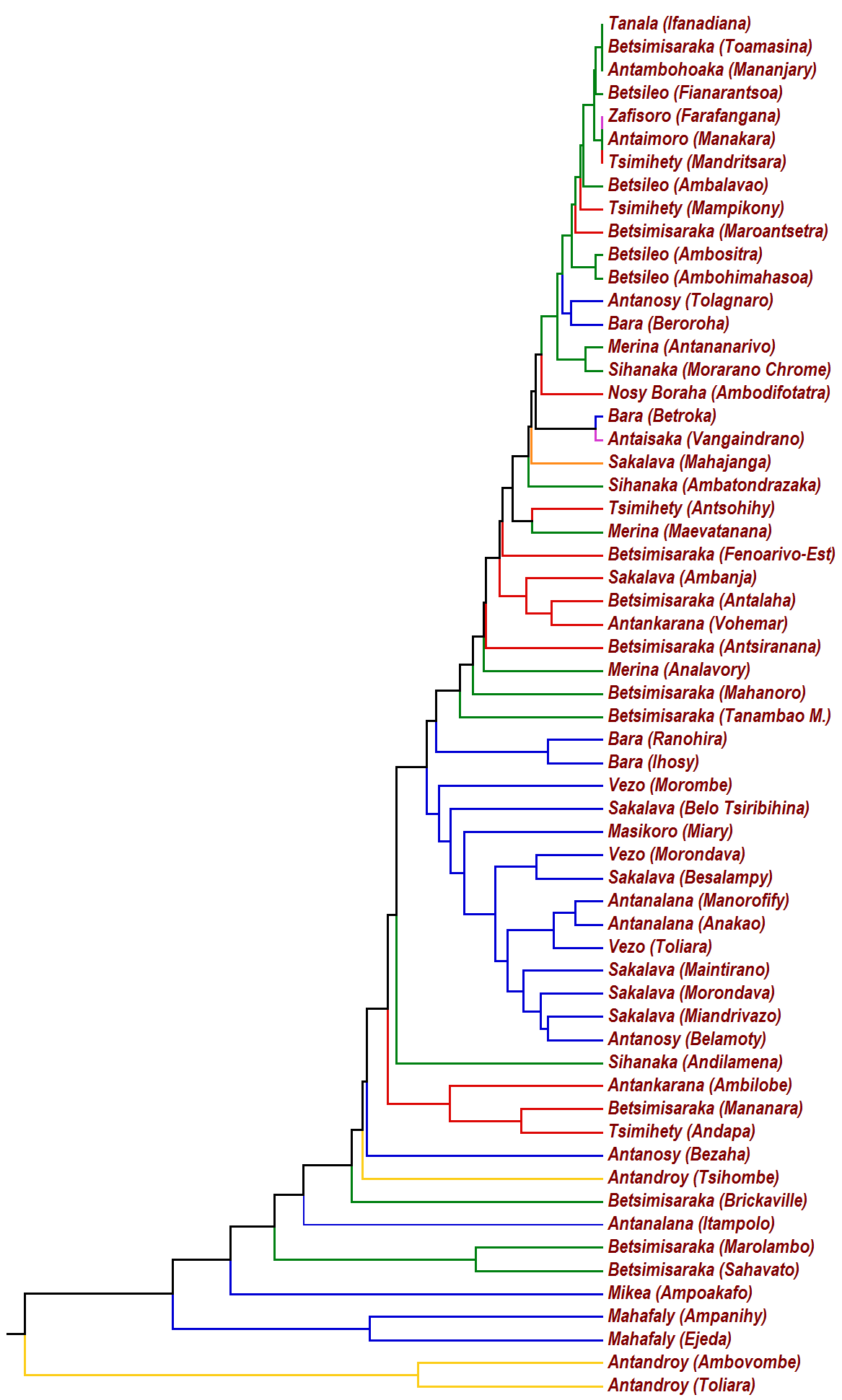

Supplement: S1 Fig — The UPGMA tree generated by the Swadesh lists which only contain the 35 most stable items for the family of Malagasy variants. (PNG) [file pone.0240170.s004.png]

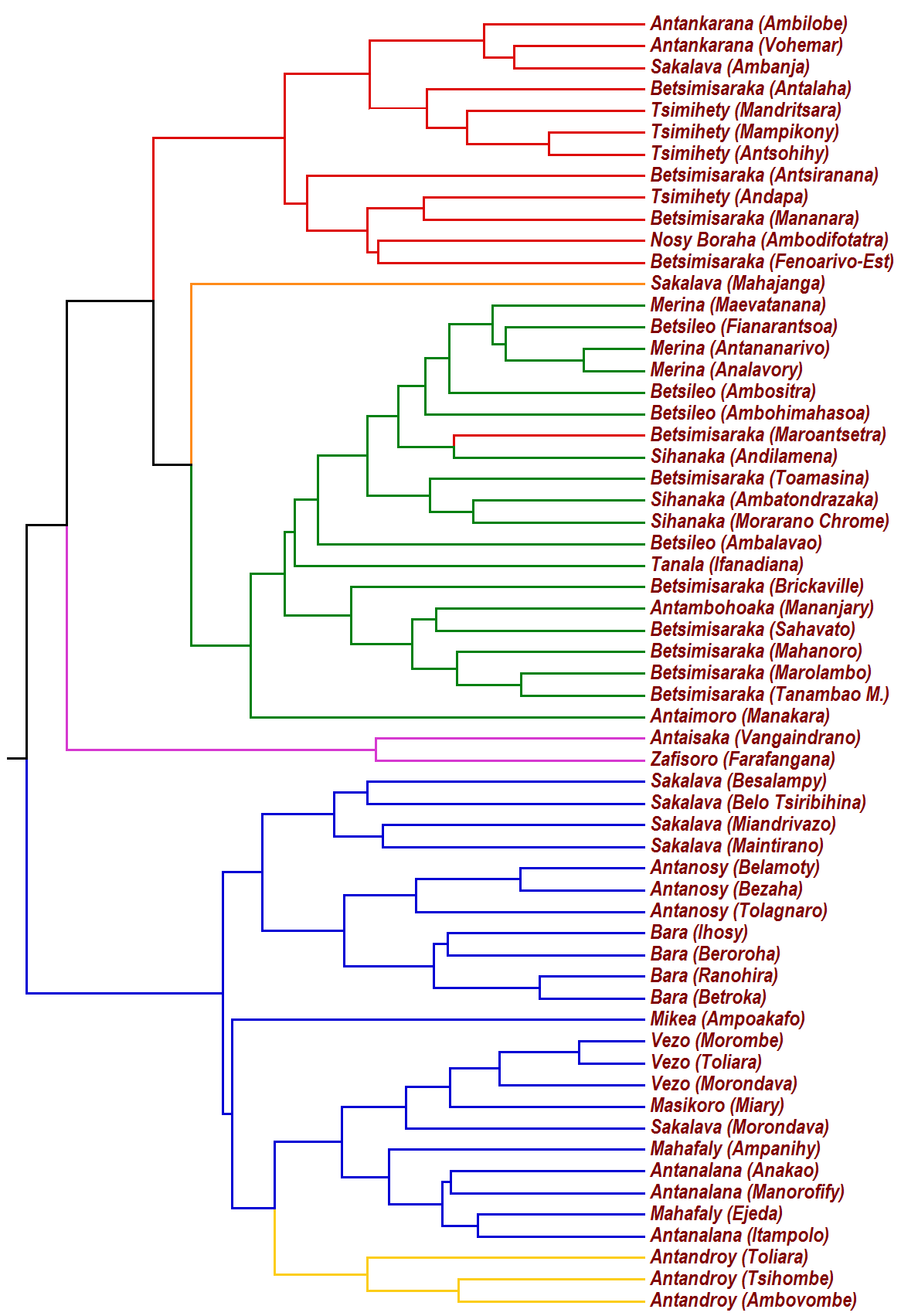

Supplement: S2 Fig — The UPGMA tree generated by the Swadesh lists which only contain the 35 most stable items for the family of Malagasy variants. (PNG) [file pone.0240170.s005.png]

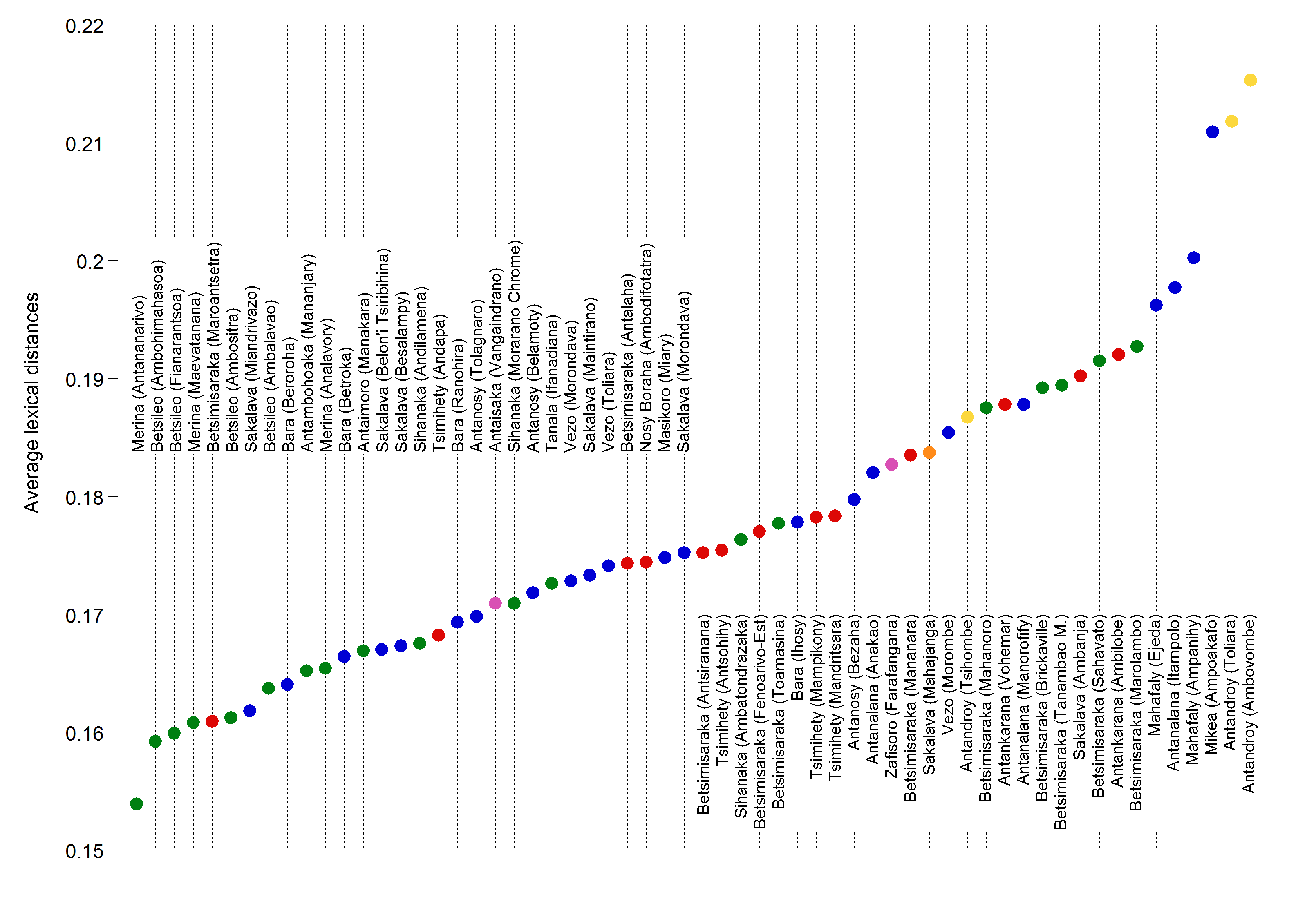

Supplement: S3 Fig — The average lexical distance of each dialect from the others. The 60 varieties are ranked according to the value of the average. (PNG) [file pone.0240170.s006.png]
